# Piezoelectric Amino Acid Salts with Halogen‐Based Coformers Produce Exceptional Force‐Generated Voltage Output

**DOI:** 10.1002/advs.78588

**Published:** 2026-11-05

**Authors:** Krishna Hari, Shubham Vishnoi, Sarah Guerin

**Affiliations:** ^1^ Department of Chemical Sciences Bernal Institute University of Limerick Limerick Ireland; ^2^ SSPC The Research Ireland Research Centre for Pharmaceuticals University of Limerick Limerick Ireland

**Keywords:** basic amino acids, crystal engineering, density functional theory, materials science, piezoelectric coefficient, piezoelectric energy harvesting, piezoelectricity, piezoresponse force microscopy, rational design, sustainable solutions

## Abstract

Hierarchical self‐assemblies of bio‐inspired materials combine structural tunability with biocompatibility and environmental sustainability for multifunctional applications. Within this area of applied research, minimalistic building units such as amino acids and their derivatives have evolved as next‐generation piezoelectric materials. Their tendency towards chiral nature, polar molecular structure, asymmetric crystal packing, and designability enable efficient linear voltage generation under mechanical stress, yet their technological translation is at formative stages. Amino acid‐based multicomponent crystals (**MCC**s) are an emerging class of sustainable piezoelectrics, which benefit from an integrated experimental‐theoretical approach for commercial advancement. In this work, the piezoelectric properties of nine non‐centrosymmetric amino acid salt crystals were confirmed experimentally using both piezoresponse force microscopy (PFM) and *quasi*‐static piezoelectric measurements (*d_33_* = 7.8 pC/N) and calculated using density functional theory (DFT), with a maximum predicted longitudinal piezoelectric coefficient of 7.5 pC/N. A large open‐circuit voltage of 12.9 V was obtained for polycrystalline L‐histidinium trifluoroacetate under direct ambient mechanical stimuli, exceeding the open‐circuit voltage of ~6 V of a commercial ceramic disc. The close agreement between measured and computed electromechanical properties allows for correlations to be drawn between the **MCC** molecular structures and their macroscopic properties, facilitating the rational design of sustainable organic piezoelectrics.

## Graphical abstract

Amino acid‐based multicomponent crystals have emerged as high‐performance, environmentally sustainable piezoelectrics. In this work, the correlation of crystal packing, molecular polarization, and electromechanical response is uncovered through integrated experimental and computational approaches. Notably, the high output voltage from L‐histidinium trifluoroacetate highlights the potential of these molecularly engineered organic crystals for next‐generation energy harvesting technologies.

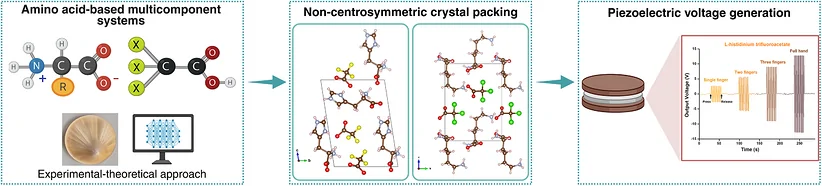

## 1 Introduction

Piezoelectric materials, due to their ability to linearly interconvert between mechanical energy and electrical energy in non‐centrosymmetric crystalline structures (Figure 1A), possess significant potential for multifunctional applications [1, 2, 3, 4, 5]. Lead‐based ceramic materials that dominate commercial piezoelectric electronics are not only detrimental to health and the environment but also face issues of brittleness, lack of tunability, and often require complex processing and multi‐layer structures [6]. Therefore, as the implementation of lead‐free materials is an imminent requirement for industrial regulations and performance, organic materials are a promising replacement for toxic heavy metal‐based piezoelectric materials [7, 8, 9, 10, 11], but currently have reduced commercial visibility.

**FIGURE 1:**
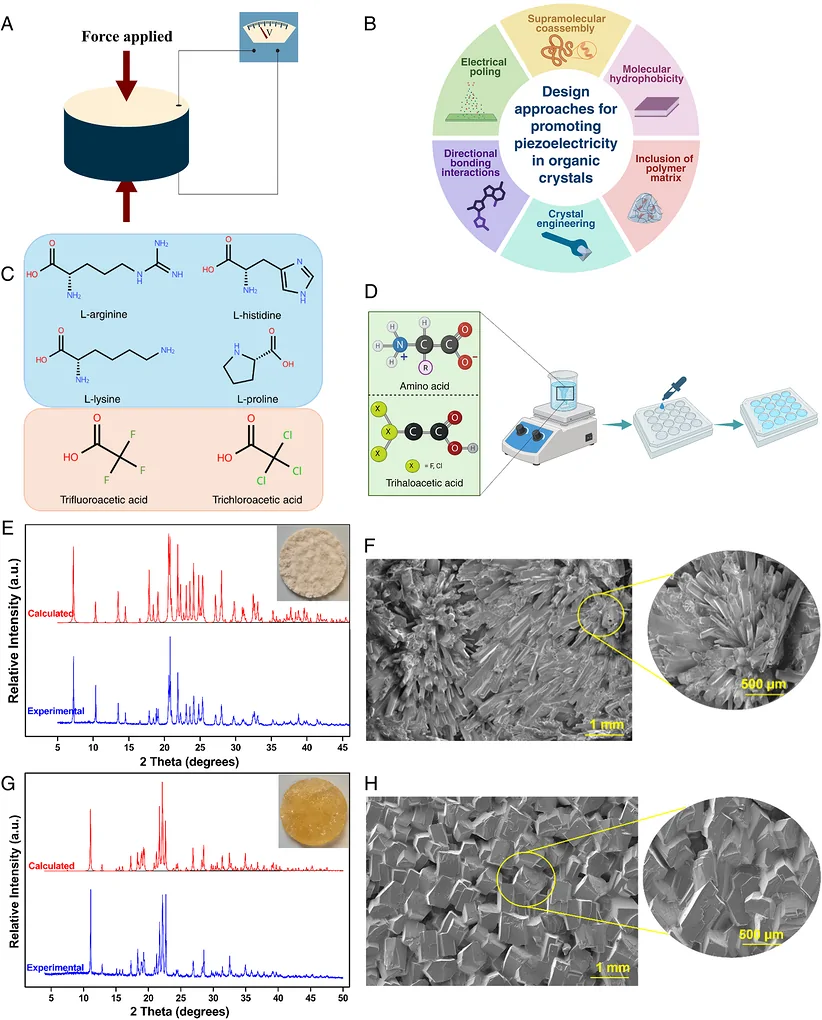
(A) Scheme showing the piezoelectric effect where linear mechanical force is converted into an electrical signal in non‐centrosymmetric materials. (B) Different design approaches adopted for enhancing the piezoelectricity in organic materials. (C) Scheme showing the coformers of the multicomponent salts based on amino acids along with their chemical structures. (D) Scheme of the molding methodology employed for the fabrication of polycrystalline stand‐alone elements in this work. (E) PXRD patterns and photograph (inset) of the formed stand‐alone discs of L‐histidinium trifluoroacetate. (F) Scanning electron micrographs of L‐ histidinium trifluoroacetate (Scale: 1 and 500 µm). (G) PXRD patterns and photograph (inset) of the formed stand‐alone discs of L‐lysinium trifluoroacetate. (H) Scanning electron micrographs of L‐lysinium trifluoroacetate (Scale: 1 mm and 500 µm).

Numerous design approaches for enhancing piezoelectric properties and technological viability in organic materials include supramolecular coassemblies [12], improving molecular hydrophobicity [13, 14, 15, 16], inclusion of a polymer matrix [17, 18], electrical poling [19, 20, 21, 22, 23], promoting directional bonding interactions [24], and crystal engineering [25, 26] (Figure 1B). From this perspective, crystal engineering to form multicomponent systems, by virtue of its tunability, scalability, and versatility holds huge potential to unlock piezoelectric properties by fine‐tuning diverse chemistries, thereby, enhancing self‐assembly of the building blocks and altering multiscale structural, chemical, electronic, and mechanical properties [26, 27, 28]. Halogenated materials and halogen functionalization, for instance, supramolecular assemblies of organofluorine peptides exhibit diverse biofunctionalities—with applications in ion transport, drug delivery, and tissue engineering [29, 30, 31, 32, 33]. Fluorination or hydrogenation of single‐layer hexagonal boron nitride demonstrates in‐plane and out‐of‐plane piezoelectricity [34, 35]. Recently, mono and pentafluorination of L‐phenylalanine sidechains promoted their self‐assembly and thereby enabled these materials to be applied for a tactile self‐sensor for sorting nuts [36].

Although crystal engineering‐based multicomponent systems of bio‐organic materials have been extensively studied for pharmaceutical applications, these materials are only beginning to be explored for technologically relevant piezoelectric applications [37, 38, 39, 40]. Thus, crystal engineering represents a favorable but sparsely used area for the investigation of biopiezoelectrics—hence the need to leverage both computational and experimental strategies to discover and fabricate high‐performing piezoelectric bio‐organic materials. In this work, we utilised crystal engineering, structural characterization, and density functional theory calculations to develop trihaloacetate salts of amino acids such as L‐arginine, L‐lysine, L‐histidine, and L‐proline. This methodology allows us as crystal engineers to embed and modulate piezoelectric performance in standardized stand‐alone device components for multifunctional applications.

## 2 Results and Discussion

### 2.1 Trifluoroacetic acid‐Amino Acid Multi‐Component Systems

The functional properties of biomolecular assemblies are influenced by alterations in their molecular architectures, which allows for modulation of structure‐property relationships [41]. Figure 1C shows the chemical structures of the amino acids and coformers used in this study. A rational design strategy was used to favor asymmetric charge distribution, with the highly electronegative coformers enhancing the charge anisotropy and polarization in the crystal lattice [42]. L‐histidinium trifluoroacetate crystallizes in the triclinic non‐centrosymmetric *P*_1_ space group [43], whereas L‐lysinium trifluoroacetate crystallises in the non‐centrosymmetric monoclinic *P*2_1_ space group [44]. Both were crystallized herein according to the literature. The multicomponent salt crystal structures formed by the component molecules is sustained by three‐dimensional hydrogen bonding networks, with the amino **N** atom of the amino acid cation forming **N‐H···O** hydrogen bonds with **O** atoms of the trifluoroacetate anion, giving rise to the supramolecular packing. The fabrication of stand‐alone polycrystalline discs of the multicomponent salt systems was then carried out using the previously reported molding methodology [45], as given in Figure 1D, to evaluate the suitability of the molding workflow in diverse chemistries (Note S1). Consequently, L‐histidinium trifluoroacetate was fabricated into circular discs of diameter = 3.3 cm using silicone molds and the bulk purity of the salt crystals agreed with the calculated PXRD pattern, and SEM revealed an oriented needle‐shaped 3D cohesive assembly (Figure 1E,F). In the case of L‐lysinium trifluoroacetate, when fabricated into circular discs (diameter = 3.3 cm), satisfactory bulk purity was also confirmed using PXRD. Surface morphology presented as a block‐like arrangement for the polycrystalline assemblies, as shown in Figure 1G,H. From the thermal analysis, it can be seen that L‐histidinium trifluoroacetate and L‐lysinium trifluoroacetate are stable up to 200°C; therefore, they can be safely employed for piezoelectric applications within this window, and DSC showed the absence of an endothermic peak, indicating no signs of phase transitions (Figure S1). The discs of L‐argininium trifluoroacetate and L‐argininium bis(trifluoroacetate) salts were also crystallized according to previous reports [46, 47]. Although they belong to the non‐centrosymmetric monoclinic *P*2_1_ space group, their polycrystalline forms were fragile and prone to breakage and were not utilized for any electromechanical tests (Tables S1–S4).

### 2.2 Trichloroacetic acid‐Amino Acids Multi‐Component Systems

Following the previous reports, trichloroacetic acid salts of L‐histidine [48, 49], L‐lysine [50, 51], and L‐proline [52, 53] were crystallized into their non‐centrosymmetric space groups, characterized, and made into circular free‐standing discs with a diameter = 3.3 cm for piezoelectric studies. The multicomponent crystal structures of these trichloroacetates were mainly driven by **N‐H···O** intermolecular interactions between the chemical moieties. Further, Figure 2A shows that the bulk purity of the L‐histidinium trichloroacetate, crystallising in the monoclinic *P*2_1_ space group, was confirmed by PXRD, showing good agreement with the calculated PXRD pattern and SEM images revealed a rod‐like surface morphology with aligned crystallites, resulting in a three‐dimensional cohesive polycrystalline assembly for L‐histidinium trichloroacetate as in Figure 2B. For L‐lysinium trichloroacetate (monoclinic *P*2_1_ space group), the experimental PXRD patterns agreed with the simulated PXRD pattern with plate‐like crystallites revealed by SEM (Figure 2C,D). Moreover, the devices assembled using L‐histidinium trichloroacetate and L‐lysinium trichloroacetate showed thermal stability up to 150 and 120°C, respectively, as per TGA and DSC analyses (Figure S1). L‐prolinium trichloroacetate crystals belong to the non‐centrosymmetric space group *P*3_1_ *_,_* which is rare for complexes based on amino acids and carboxylic acids [52, 53]. Once the bulk purity was confirmed, L‐prolinium trichloroacetate was made into stand‐alone elements where various parameters such as concentration, temperature of the solution, volume added to the silicone molds, and multiple layering of the solution were optimized, and after slow evaporation, prismatic crystals were formed rather than continuous discs, whereas SEM images showed block‐like surface morphology and these single‐crystal type samples were used for piezoelectric measurements (Figure 2E,F). Similar to L‐prolinium trichloroacetate crystals, glycinium trichloroacetate and guanidinium trichloroacetate, both crystallising in the tetragonal *P*4_1_ space group [54, 56], formed as sparse polycrystalline aggregates rather than uniform free‐standing elements; they were not employed for further electrical or mechanical tests.

**FIGURE 2:**
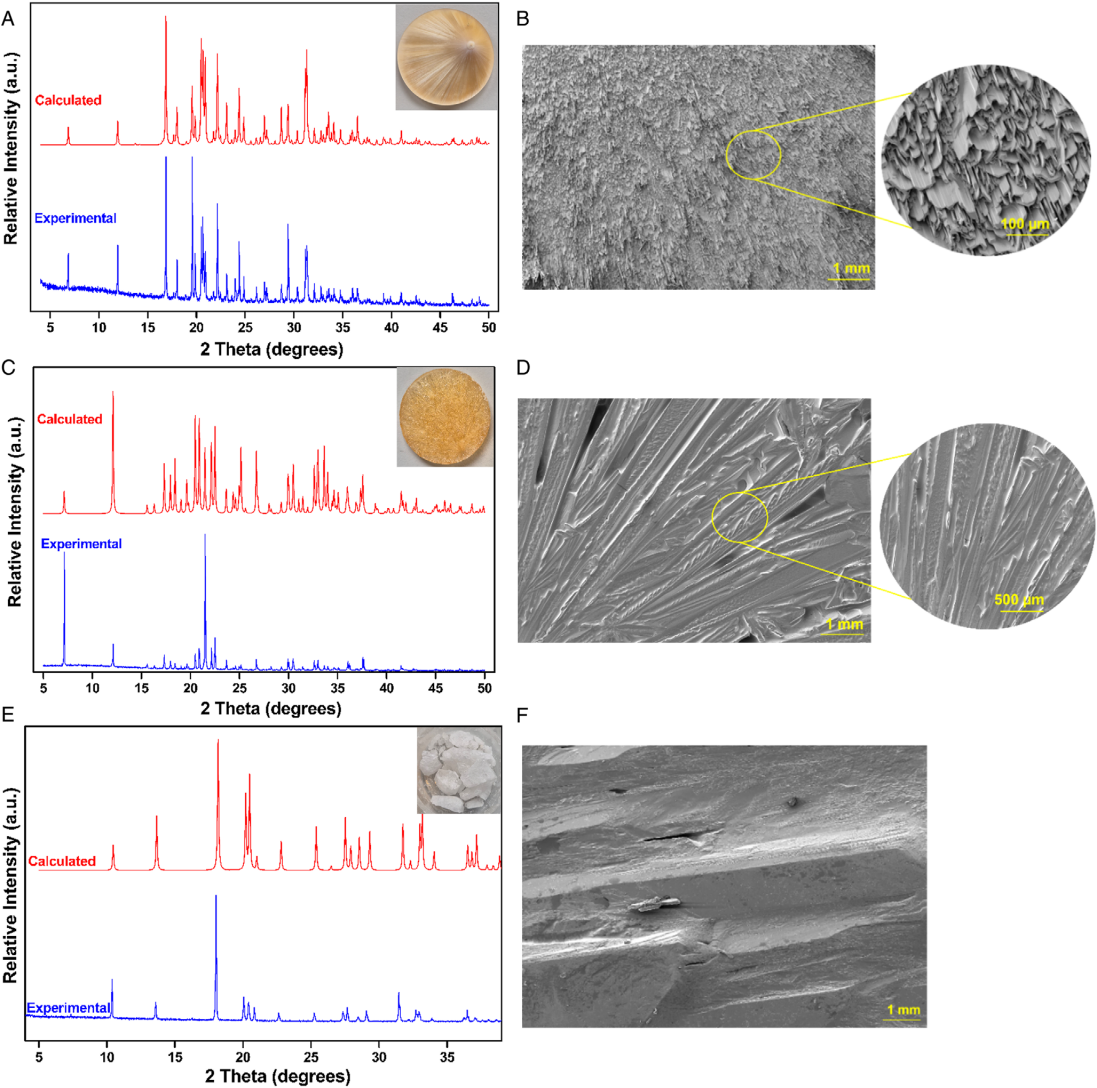
(A) PXRD patterns and photograph (inset) of the formed stand‐alone discs of L‐histidinium trichloroacetate. (B) Scanning electron micrographs of L‐histidinium trichloroacetate (Scale: 1 mm and 100 µm). (C) PXRD patterns and photograph (inset) of the formed stand‐alone discs of L‐lysinium trichloroacetate. (D) Scanning electron micrographs of L‐lysinium trichloroacetate (Scale: 1 mm and 500 µm). (E) PXRD patterns and photograph of the formed stand‐alone discs of L‐prolinium trichloroacetate. (F) Scanning electron micrograph of L‐prolinium trichloroacetate (Scale: 1 mm).

### 2.3 First‐Principles Investigation of Piezoelectric Response in Trihaloacetates of Amino Acids

We carried out a systematic theoretical study of amino acid‐based multicomponent salts formed with trihaloacetic acid, crystallizing in non‐centrosymmetric space groups using our *in‐house* CrystalDFT workflow [57]. Table 1 summarizes the crystallized systems, their space groups, and permitted piezoelectric response, focusing on triclinic, monoclinic, trigonal, and tetragonal lattices that allow both longitudinal and shear piezoelectric constants (Scheme S1). The complete computational methodology and details are provided in Note S2, with additional crystal system information listed in Table S5. Using Density Functional Theory (DFT), we investigated their piezoelectric behavior to elucidate the role of hydrogen and halogen‐driven interactions in bioorganic piezoelectric crystal design. Several amino acid crystals are known to exhibit intrinsic piezoelectricity due to non‐centrosymmetric packing, with previous studies highlighting significant longitudinal responses in simple amino acid systems [57, 58, 59]. The distinctive bonding arrangements present in these salts, combined with relatively small unit cells, make them ideal for high‐accuracy computational analysis and comparison with established organic piezoelectric crystals. Although trihaloacetate units have been used in crystal engineering and molecular self‐assembly [60, 61, 62], their piezoelectric performance as molecular crystals remains underexplored. The predicted single‐crystal DFT values are compared to their experimental longitudinal piezoelectric response at both the nanoscale and macroscale via piezoresponse force microscopy (PFM) and *quasi*‐static piezometry, respectively.

**TABLE 1:** Amino acid‐based multicomponent salts investigated in this study with their CSD identifiers, corresponding crystallographic space groups, piezoelectric symmetry allowance, macroscopic longitudinal piezoelectric strain constants (pC/N), DFT‐predicted longitudinal strain constants (pC/N), and their predictive mechanical stability. The full piezoelectric tensor data are provided in Table S17.

Amino Acid Trihaloacetates	CSD ID	Space Group	Piezoelectricity Permitted	Macroscopic Longitudinal Piezoelectric Strain Constants (pC/N)	DFT‐Predicted Longitudinal Piezoelectric Strain Constants (*d_33_^eff^*, pC/N)	Predicted Mechanical Stability
*L‐histidinium trifluoroacetate*	QAYTEQ	*P*_1_	Longitudinal, Shear	*d_33_ = 3.8*	*d_11_ = 4, d_22_* = 3.4*, d_33_* = 3.9	*Stable*
*L‐lysinium trifluoroacetate*	KIWKIL	*P*2_1_	Longitudinal, Shear	*d_33_ = 1.3*	*d_22_* = 1.2	*Stable*
*L‐argininium trifluoroacetate*	ADIHOL	*P*2_1_	Longitudinal, Shear	*d_33_ = 3.5*	*d_22_* = 4.3	*Stable*
*L‐argininium bis(trifluoroacetate)*	INEFIQ INEFIQ01	*P*2_1_	Longitudinal, Shear	*d_33_ = 4.3*	*d_22_* = 5.9 *d_22_* = 6.4	*Stable*
*L‐histidinium trichloroacetate*	IDAZET	*P*2_1_	Longitudinal, Shear	*d_33_ = 4.4*	*d_22_* = 7.7	*Stable*
*L‐lysinium trichloroacetate*	TEZTIC	*P*2_1_	Longitudinal, Shear	*d_33_ = 3.5*	*d_22_* = 2.1	*Stable*
*L‐prolinium trichloroacetate*	XUYVAO	*P*3_1_	Longitudinal, Shear	*d_33_ = 7.8*	*d_33_* = 7.5	*Stable*
*Glycinium trichloroacetate*	HEGFEF	*P*4_1_	Longitudinal, Shear, Transverse	—	*d_33_* = 1.4	*Stable*
*Guanidinium trichloroacetate*	EYIQIO EYIQIO01	*P*4_3_ *P*4_1_	Longitudinal, Shear, Transverse	—	*d_33_* = 0.6 *d_33_* = 0.3	*Stable*

Table 1 summarizes the crystallographic and piezoelectric properties of the amino acid‐based multicomponent salts formed with trihaloacetic acids investigated in this study. All multicomponent salts crystallize in space groups that permit both longitudinal and shear piezoelectric responses, while two of the salts, glycinium trichloroacetate, and guanidinium trichloroacetate and its corresponding enantiomorph, adopt tetragonal symmetry (*P*4_1_ and *P*4_3_), and are confirmed to be mechanically stable following the crystallographic estimation of their shear elastic constant (*C*_66_), as discussed in Note S2 and Table S5. The DFT‐predicted longitudinal strain constants (*d_33_^eff^*) show substantial variation across the series, reflecting the influence of amino acid identity and the nature of coformers on the electromechanical response.

These trends are further illustrated in Figure 3 and Figure S2, which present the unit‐cell structures alongside three‐dimensional visualizations of the piezoelectric strain tensors. The unit‐cell representations highlight the structural diversity and molecular packing motifs of the non‐centrosymmetric salts, while the tensor visualizations reveal the directional dependence and magnitude of the predicted electromechanical responses. Surface contours illustrate the anisotropic behavior arising from crystal symmetry and atomic arrangement, showing a clear link between mechanical deformation and induced polarization along distinct crystallographic directions (Figure S2).

**FIGURE 3:**
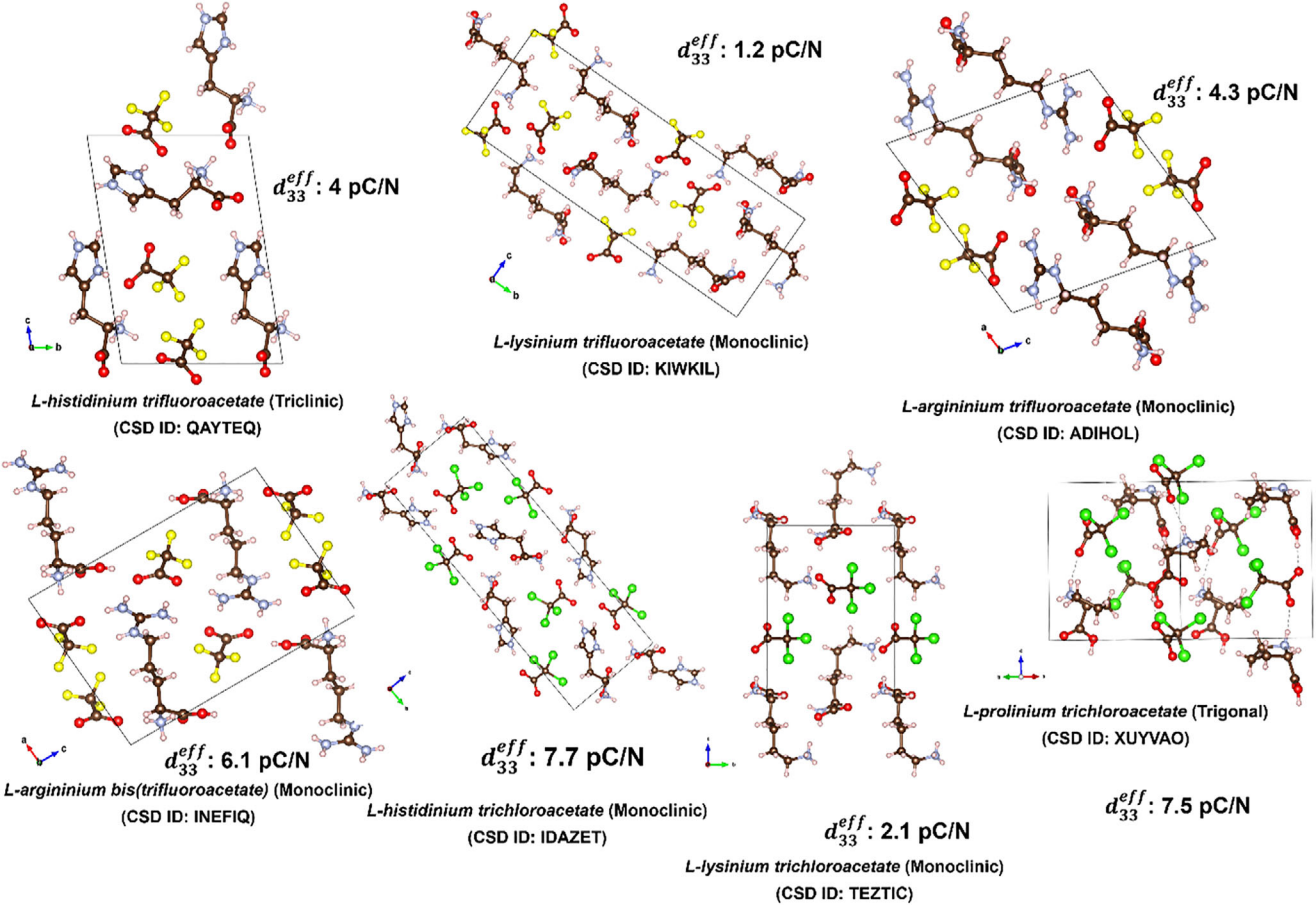
Unit‐cell structures of the amino acid‐based multicomponent crystal systems, illustrating the structural diversity and molecular packing motifs of the non‐centrosymmetric salts. The corresponding DFT‐predicted longitudinal piezoelectric strain constants (*d_33_^eff^*, pC/N) are indicated for each structure, highlighting variations in the calculated electromechanical response.

Figure 4A shows that five circular discs (diameter = 3.3 cm) of L‐histidinium trifluoroacetate provided an average macroscale *d_33_* in the range of 1.93 ± 0.80 to 3.81 ± 1.15 pC/N, with average thickness ranging between 1.96 ± 0.08 and 2.67 ± 0.04 mm (Tables S6 and S7). Also, L‐histidinium trifluoroacetate (QAYTEQ) exhibits a maximum nanoscale longitudinal piezoelectric response *d_33_* = 3.8 pC/N and an average nanoscale longitudinal piezoelectric response (*d_33_*) of 3.06 ± 0.58 pm/V (Figure 4B and Figure S3A), while the corresponding DFT‐predicted effective longitudinal piezoelectric constant is *d_33_^eff^* = 3.9 pC/N. In contrast, the circular discs of L‐lysinium trifluoroacetate (KIWKIL) with an average thickness varying from 2.04 ± 0.22 to 5.03 ± 0.12 mm demonstrate an average macroscale longitudinal piezoelectric response (*d_33_*) varying from 0.30 ± 0.09 to 1.30 ± 0.20 pC/N (Tables S8 and S9), in agreement with the DFT‐predicted longitudinal response of *d_22_* = 1.2 pC/N. Overall, these comparisons highlight that while DFT calculations reliably reproduce the relative trends among different compounds, absolute values can differ from experimental measurements, emphazising the combined role of trihaloacetate salt morphology andpolycrstalline self‐assembly in determining both nanoscale and macroscale piezoelectric performance.

**FIGURE 4:**
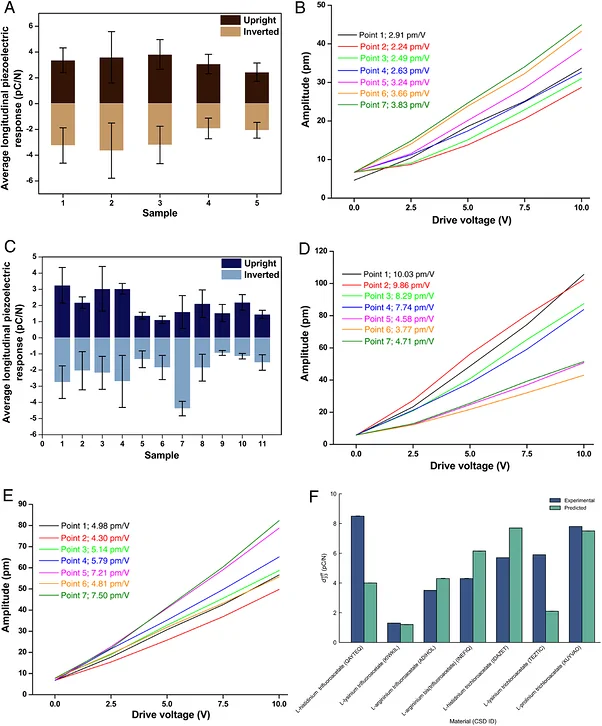
(A) Macroscale average longitudinal piezoelectric response (*d_33_*) of L‐histidinium trifluoroacetate. (B) Quantified longitudinal piezoelectric response (*d_33_*) obtained in PFM measurements of L‐histidinium trifluoroacetate. (C) Macroscale average longitudinal piezoelectric response (*d_33_*) of L‐histidinium trichloroacetate. Quantified longitudinal piezoelectric response (*d_33_*) obtained in PFM measurements of (D) L‐histidinium trifluoroacetate. (E) L‐prolinium trichloroacetate. (F) Experimental (macroscale) and DFT‐predicted longitudinal piezoelectric strain constants (*d_33_^eff^*, pC/N) for amino acid salts formed with trihalogenated acetic acids. The comparison highlights the agreement between experimental measurements and theoretical predictions for the materials investigated in this study.

For the trichloroacetate salts, L‐histidinium trichloroacetate (IDAZET) shows an average macroscale longitudinal piezoelectric response varying from 0.94 ± 0.15 to 4.38 ± 0.45 pC/N (Figure 4C) with average thickness ranging from 2.50 ± 0.32 to 3.46 ± 0.06 mm over 11 circular discs of 3.3 cm diameter (Tables S10 and S11). A maximum nanoscale longitudinal piezoelectric response of *d_33_* = 10 pm/V and an average nanoscale longitudinal piezoelectric response of *d_33_* = 6.99 ± 2.42 pm/V were obtained for L‐histidinium trichloroacetate (see Figure 4D and Figure S3B), whereas the DFT predicts a directional piezoelectric component *d_22_* = 7.7 pC/N. These differences are similar to the extent of prior reports in some organic piezoelectric systems, where idealized periodic DFT calculations often differ from macroscopic responses due to the absence of defects, grain boundaries, and domain contributions present in experimental polycrystalline samples [63, 64]. L‐prolinium trichloroacetate (XUYVAO) displays a maximum nanoscale longitudinal piezoelectric response *d_33_* = 7.5 pm/V, an average nanoscale longitudinal piezoelectric response *d_33_* = 5.68 ± 1.14 pm/V, and a maximum macroscale longitudinal piezoelectric response *d_33_* = 7.8 pC/N (see Figure 4E, Figure S3C, and Note S3). These are in close agreement with the DFT‐predicted effective longitudinal constant (*d_33_^eff^* = 7.5 pC/N), indicating that the computational model accurately captures the polarization along the primary axis. In contrast, L‐lysinium trichloroacetate (TEZTIC) circular discs demonstrate an average thickness varying from 2.17 ± 0.29 to 3.42 ± 0.14 mm and an average macroscale *d_33_* response between 1.11 ± 0.80 to 3.53 ± 1.09 pC/N (Tables S12 and S13). The values are close to the DFT‐predicted longitudinal component *d_22_* = 2.1 pC/N, indicating good agreement between experiment and theory and suggesting a high degree of dipole orientation within the polycrystalline discs. Figure 4F compares the experimental (macroscale) and DFT‐predicted longitudinal piezoelectric constants (*d_33_*) for all amino acid‐based multicomponent salts. These results demonstrate that strategic incorporation of halogen‐mediated interactions can modulate polarization and enhance piezoelectric properties, emphasizing the potential of these bioorganic crystals for piezoelectric sensor and energy‐harvesting applications. This clearly shows that both experimental measurements and theoretical calculations consistently reproduce the relative trends across the series, highlighting the key roles of amino acid identity, coformers, and crystal packing in determining the piezoelectric performance of these bioorganic salts.

The quantification of orientation distribution within the polycrystalline assemblies of amino acid trihaloacetates proved challenging using wide‐angle X‐ray scattering (WAXS) (Note S4 and Figure S4), therefore, the local crystallite orientation in the fabricated discs was determined from the SEM micrographs using the degree of coherency in the OrientationJ Measure plugin available in ImageJ software [65, 66]. The degree of coherency ranges from 0 to 1, where 0 corresponds to randomly oriented crystallites, and 1 represents a fully aligned crystallite assembly with a well‐defined preferred orientation [67]. As given in Figure 5, the calculated degree of coherency across the fabricated discs of L‐histidinium trifluoroacetate, L‐lysinium trifluoroacetate, L‐histidinium trichloroacetate, and L‐lysinium trichloroacetate were 0.62 ± 0.12, 0.57 ± 0.05, 0.71 ± 0.07, and 0.61 ± 0.15. Therefore, the obtained piezoelectric response of the fabricated discs of the multicomponent salts of amino acids can be attributed to the local orientation of the crystallites within the polycrystalline assemblies, as revealed by the calculated degree of coherency (Table S14). It should be noted that OrientationJ Measure reflects only the local crystallite alignment and not any bulk crystallographic orientation (Note S4).

**FIGURE 5:**
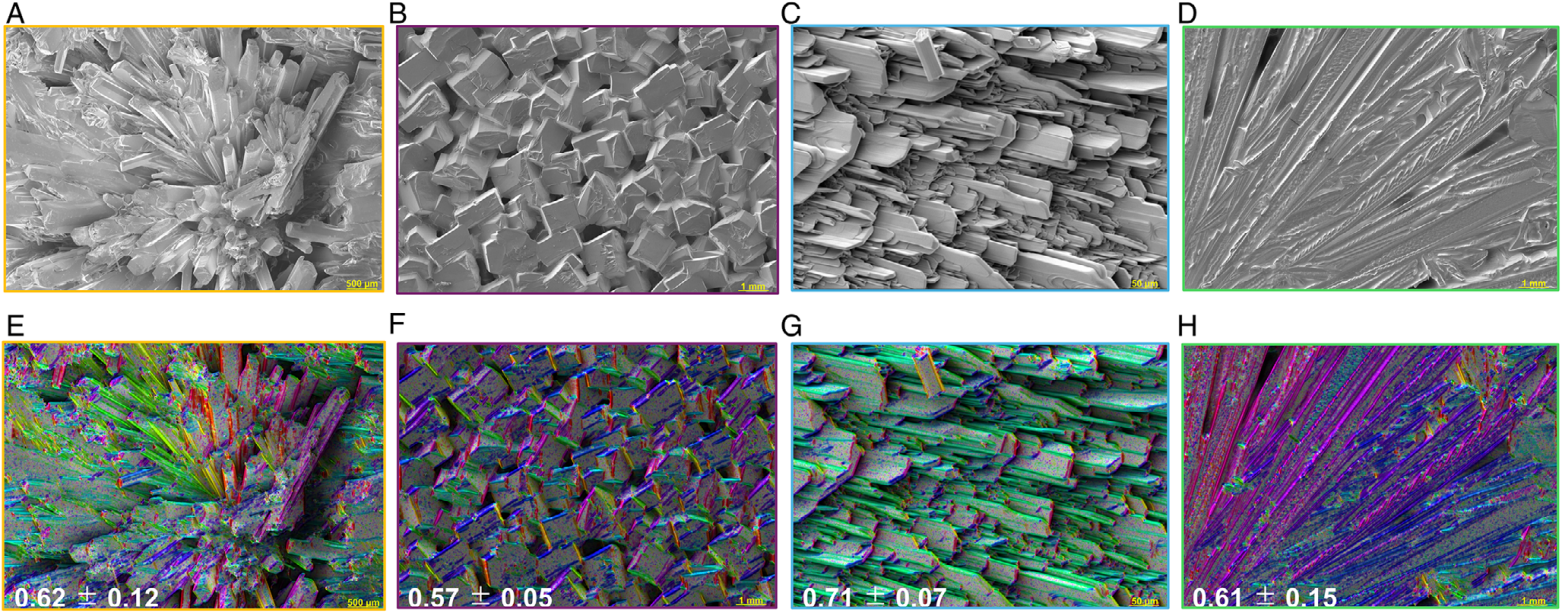
Quantitative analysis of local crystallite orientation performed on SEM micrographs of amino acid trihaloacetates using the degree of coherency in OrientationJ Measure plugin available in ImageJ. SEM micrographs of (A) L‐histidinium trifluoroacetate (Scale: 500 µm). (B) L‐lysinium trifluoroacetate (Scale: 1 mm). (C) L‐histidinium trichloroacetate (Scale: 50 µm). (D) L‐lysinium trichloroacetate (Scale: 1 mm). Orientational colour analysis and calculated degree of coherency of (E) L‐histidinium trifluoroacetate (Scale: 500 µm). (F) L‐lysinium trifluoroacetate (Scale: 1 mm). (G) L‐histidinium trichloroacetate (Scale: 50 µm). (H) L‐lysinium trichloroacetate (Scale: 1 mm).

To evaluate the halogen substitution effect, comparative Hirshfeld surface and 2D fingerprint plot analyses were performed and mapped with a standardized *d_norm_* scale from −0.5 to 1.5 au (Figure S5 and Table S15). Highly electronegative fluorine atoms restrict their interaction areas primarily to localized F—H contacts, reaching 31.8% in KIWKIL. In contrast, trichloroacetate structures exhibit a redistribution of surface interactions toward heavy‐atom pathways. In TEZTIC, the Cl—H contact area contracts to 27.5%, while Cl—Cl and Cl—O interactions expand to 2.6% and 6.9%, respectively. For the *Z’* = 2 systems (QAYTEQ vs. IDAZET), independent evaluation of each ion pair confirms that the trichloroacetate lattice maintains dense heavy‐atom contacts across both components, with Cl—Cl and Cl—O interactions reaching 6.5% and 4.3%, respectively. This transition from rigid Halogen—H restrictions to multi‐channel heavy‐atom interactions provides support of the softer, more polarizable electron cloud of the chlorine atom. This electronic flexibility yields a more compliant intermolecular framework, driving the increased longitudinal piezoelectric response (*d*_33_ *^eff^*) from 4 pC/N in QAYTEQ to 7.7 pC/N in IDAZET.

Across the full piezoelectric coefficient matrix, the dominant signal is not confined to the longitudinal components. While the largest diagonal magnitudes occur at longitudinal *d_33_^eff^*, the most substantial deviation across the matrix occurs in the predicted off‐diagonal component *d_23_*, which reaches an absolute magnitude of 23 pC/N in L‐histidinium trichloroacetate (IDAZET). This value substantially exceeds all other entries in the series and represents a clear outlier within the distribution of *d_ij_* coefficients. The remaining transverse and shear components are at least threefold smaller in magnitude, indicating that the *d_23_* interaction represents the principal contribution in the dataset (Table S16). Within the monoclinic space group *P2_1_*, this anisotropy represents a structural mechanism where longitudinal strain along the *c*‐axis (*z*‐direction) yields a significant polarization response along the unique polar *b*‐axis (*y*‐direction). This coupling is rooted in the low longitudinal elastic stiffness component *C*_33_ (21.23 GPa), which is highly compliant relative to *C*_11_ (40.83 GPa) and *C*_22_ (42.89 GPa), indicating that the packing along the *c*‐axis is held by dominant dispersion forces (E_dis_ = −17.6 kJ/mol) that outweigh the localized electrostatic contributions (E_ele_ = −10.8 kJ/mol) (Figure S6). Under *z*‐axial strain, this lattice softness facilitates a cooperative hinging distortion of the flexible, halogen and hydrogen‐bonded molecular columns. This structural displacement systematically projects the ionic and electronic shifts cooperatively along the *b*‐axis. This collective transformation triggers a dominant polarization response (*e_23_* = 0.27 C/m^2^), driving the large *d_23_* coefficient. This directional coupling yields a transverse piezoelectric voltage coefficient of *g_23_* = 724.19 mV∙m/N. Consequently, orienting single‐crystal elements of L‐histidinium trichloroacetate perpendicular to the polar *b*‐axis while applying mechanical deformation along the compliant *c*‐axis represents an optimal design strategy for high‐sensitivity, self‐powered tactile, and acoustic energy harvesters.

Overall, the trichloroacetates display larger longitudinal piezoelectric constants compared with their trifluoroacetate counterparts, reflecting the influence of heavier halogen substitution and modified crystal packing on dipole alignment and electromechanical coupling. Notably, for glycinium trichloroacetate and guanidinium trichloroacetate, which crystallize in the piezoelectric‐permitting tetragonal *P4_1_* space group, mechanical stability is fully preserved across the entire stiffness matrix following the crystallographic correction for their shear elastic constant (*C*_66_) as discussed in Note S2 (Table S17). This predictive mechanical stability was verified using the generalized Born stability criteria requiring all six principal diagonal elements of the 6 × 6 stiffness matrix (*C*_ii_) to meet a stable threshold of ≥ 1 GPa [68, 69]. Numerical artifacts are strictly excluded because the finite‐difference stress derivatives were calculated using an optimized supercell model under fully converged conditions [37]. Convergence validation tests tracking total energies against plane‐wave cutoffs, relative density grids, and electronic thresholds were executed (Figures S7–S10). The energy profiles completely flatten at our chosen production thresholds (900 Ry cutoff, 60 Ry relative cutoff, and 10^−6^ Hartree threshold), confirming the calculations are fully converged. These observations support prior studies showing that a combination of molecular composition and supramolecular arrangement govern the electromechanical properties of organic piezoelectric crystals [70, 71].

Additional non‐longitudinal piezoelectric responses were identified in a few crystals where the predicted maximum strain coefficients (*d_ij_^max^*) exceed the reported longitudinal *d_33_^eff^*. Specifically, XUYVAO (*d_15_* = 5 pC/N), QAYTEQ (*d_15_* = 8.5 pC/N), IDAZET (*d_23_* = 23 pC/N), and TEZTIC (*d_16_* = 5.9 pC/N)) display shear or off‐axis contributions (Table S16). Predicted hydrogen‐bonding connectivity varies substantially across the series (3‐22 H‐bonds per unit cell; Table S16) but does not show a clear correlation with the predicted piezoelectric coefficients. Although KIWKIL and INEFIQ01 contain dense hydrogen‐bonding networks (22 and 18 H‐bonds, respectively), their piezoelectric responses differ, indicating that hydrogen‐bond count alone does not govern the electromechanical behavior. The predicted elastic behavior of the modelled molecular crystals is shown in Figure 6. The two‐dimensional projections of the elastic tensor show the directional dependence of the mechanical response, with polar plots showing variations in Young's modulus and Poisson's ratio as a function of crystallographic direction. The calculated surfaces reveal anisotropy across the modelled crystals (Figure 3), reflecting the influence of molecular packing on mechanical stiffness. Cases where the modulus varies significantly with orientation confirms that mechanical rigidity is strongly governed by the alignment of intermolecular interactions within the crystal lattice. The full 6 × 6 elastic constant tensors derived from the finite difference calculations are provided in Table S17. The calculated Hill averages indicate moderate mechanical stiffness across the series, with bulk moduli spanning 12–28 GPa and Young's moduli between ∼13.5 to 37 GPa. Among the trifluoroacetate salts, L‐argininium bis(trifluoroacetate) displays the highest predicted stiffness (E = 37.4 GPa), while L‐histidinium trifluoroacetate and L‐lysinium trifluoroacetate exhibit comparable elastic responses (E = 33–35 GPa). The trichloroacetate systems generally show slightly lower bulk and shear moduli with the guanidinium trichloroacetate representing the most compliant crystal in the set (E_Hill_ = 13.5 GPa). Across all materials, Poisson ratios cluster narrowly around 0.22–0.50, consistent with typical molecular crystals dominated by directional intermolecular interactions [72, 73]. The derived Voigt, Reuss and Hill averages for each crystal are provided in Table S17 for each crystal. Further, the crystal dipole moments span a wide range (5.1–43.9 D), with larger dipoles coinciding with higher piezoelectric responses, highlighting the role of dipolar alignment and polar packing within the lattice (Table S18).

**FIGURE 6:**
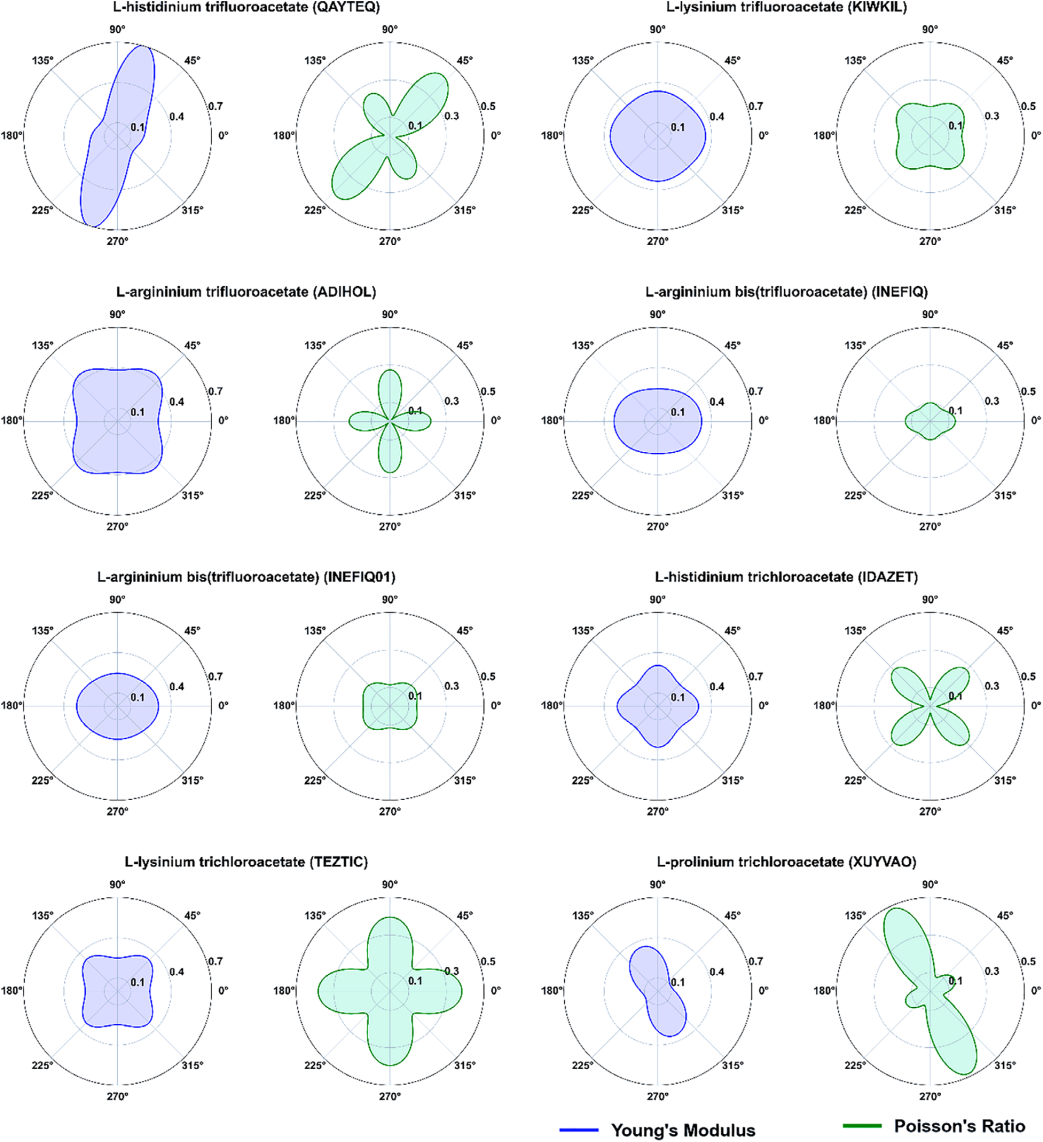
Predicted mechanical properties of modelled amino acid‐based multicomponent molecular crystals. Two‐dimensional projections of the elastic tensor, *C*, for the materials, highlighting the directional dependence of Young's modulus (GPa) and Poisson's ratio. First‐principles quantum calculations were used to systematically investigate the electronic structures and elastic properties, emphasising the anisotropic character across the simulated dataset.

Mechanical properties of the fabricated discs (diameter = 3.3 cm) of the trihaloacetates of L‐histidine and L‐lysine were evaluated using uniaxial compression loading. Figure 7 presents the linear stress‐strain curves where the slope of the linear region yielded average Young's moduli of the salt crystals in the range of 52.40 ± 0.45 GPa for L‐histidinium trifluoroacetate (Figure 7A), 29.98 ± 6.01 GPa for L‐lysinium trifluoroacetate (Figure 7B), 29.77 ± 1.79 GPa for L‐histidinium trichloroacetate (Figure 7C), and 33.31 ± 7.47 GPa for L‐lysinium trichloroacetate (Figure 7D). The microstructural heterogeneity of the polycrystalline salt crystals contributes to the observed variations in the Young's modulus of each disc under the applied uniaxial compression loading. Also, the obtained Young's moduli are consistent with literature reports of similar class of materials such as amino acids and peptides and comparable with commercial piezoelectric materials characterized under various mechanical loading conditions (Table S19). Further, the complete stress‐strain curves for the fabricated discs of amino acid trihaloacetates are provided in Figure S11. The absolute magnitude of the Young's moduli match well with individual predicted elastic stiffness components, which is consistent with the dominent crystallographic orientations in Figure 5. This integrated experimental‐computational approach offers a comprehensive understanding of the mechanical behavior of these materials, wherein the computational approach yields mechanistic insights into structure‐property relationships at the atomic/molecular level, while experimental uniaxial compression loading provides the bulk polycrystalline and realistic mechanical response of these polycrystalline elements.

**FIGURE 7:**
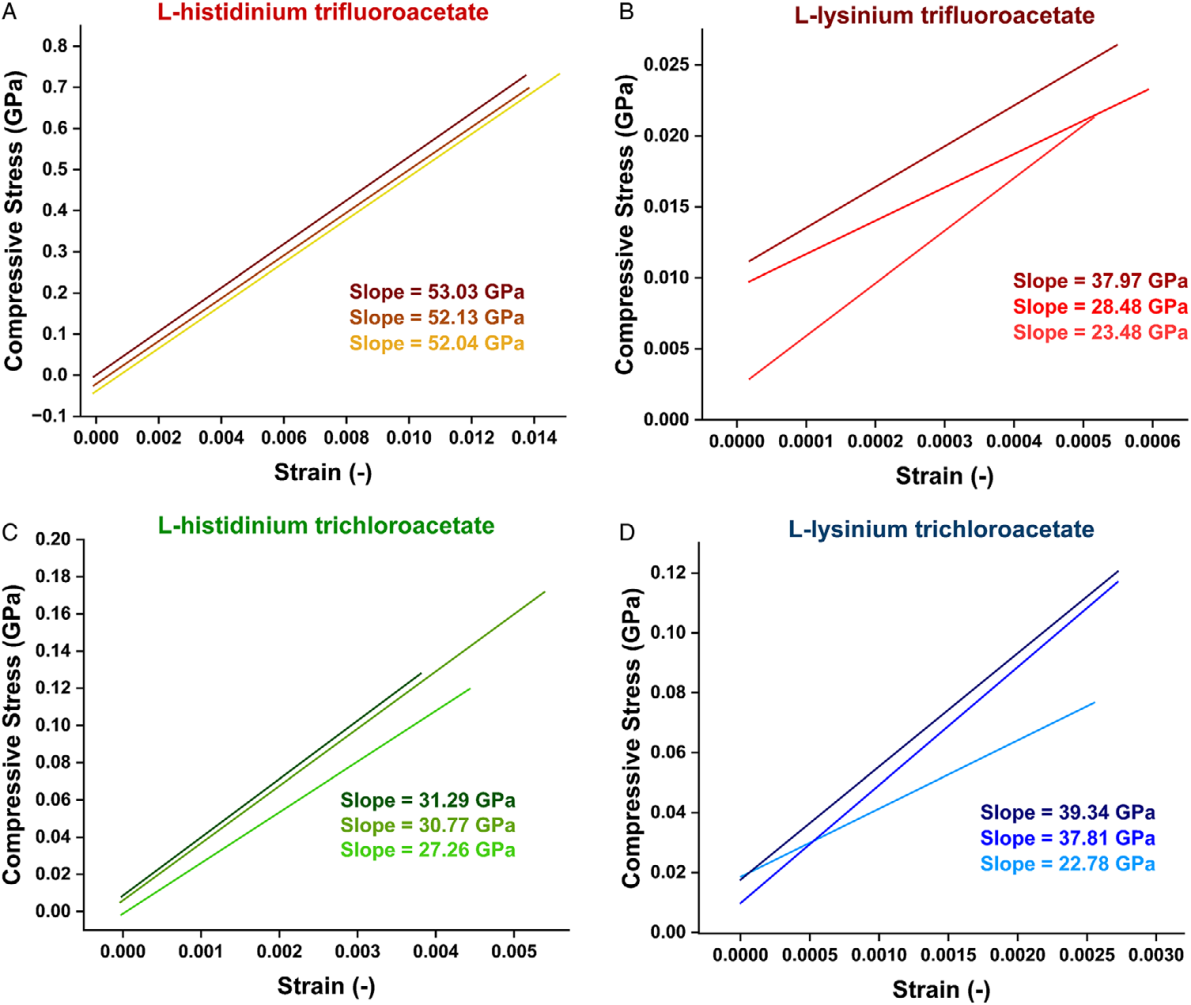
Linear regions of the stress‐strain curves with the extracted range of Young's moduli (slope of the linear line) for three discs with diameter = 3.3 cm of (A) L‐histidinium trifluoroacetate. (B) L‐lysinium trifluoroacetate. (C) L‐histidinium trichloroacetate. (D) L‐lysinium trichloroacetate.

### 2.4 Structure‐Property Relationships of Amino Acid‐Based Multicomponent Systems

The space group of the crystal determines which piezoelectric strain constants are allowed [74], and the magnitude of the piezoelectric response depends on several factors. As L‐histidinium trifluoroacetate [49] (space group: triclinic *P*_1_) and L‐histidinium trichloroacetate [75] (space group: monoclinic *P*2_1_) crystallize in different space groups, it is difficult to correlate the piezoelectric response to the supramolecular packing, as the presence and absence of the piezoelectric strain components depend on the space groups. In this context, to understand whether there is any relationship between the piezoelectric response of trifluoroacetates and trichloroacetates of the same amino acid crystallizing in the same space group, we selected L‐lysinium trifluoroacetate [76] and L‐lysinium trichloroacetate [51] crystallizing in the non‐centrosymmetric monoclinic space group *P*2_1_. As per the DFT calculations carried out, the predicted longitudinal piezoelectric strain constant (*d_22_*) of L‐lysinium trifluoroacetate and L‐lysinium trichloroacetate were 1.2 and 2.1 pC/N, respectively (Tables S15 and S16), which were further confirmed by *quasi*‐static piezoelectric measurements at the macroscale. The slight increase in piezoelectric response of these salt crystals can be attributed to the crystal packing arrangement, nature of the coformers, and packing density *Z’*. The number of crystallographically independent molecules in the asymmetric unit (*Z’*) is 2 in L‐lysinium trifluoroacetate and *Z’* = 1 in L‐lysinium trichloroacetate. This difference in *Z’* aids in the change of intermolecular networks and thereby crystal packing as molecules crystallising in *Z’* > 1 tend to be less flexible and have fewer rotatable bonds [77]. Also, the enhanced longitudinal piezoelectric response of L‐lysinium trichloroacetate may partially arise from the weaker electronegative and highly polarizable nature of the heavier chlorine atoms, allowing more flexible intermolecular interactions than the fluorine counterpart. This is consistent with recent work in our group demonstrating that chlorinated compounds have higher piezoelectric polarization compared to other halogens [32]. Further, the smaller cell volume of L‐lysinium trichloroacetate (V = 642.18 Å^3^) leads to a higher polarization density than L‐lysinium trifluoroacetate (V = 1139.99 Å^3^), contributing to a comparatively higher piezoelectric response once sufficient mechanical stimuli are provided.

### 2.5 Energy Harvesting

In order to understand the sensitivity, voltage characteristics, and energy harvesting potential of the formed piezoelectric stand‐alone elements of amino acid‐based multicomponent salts, mechanical stimuli with sporadic force intensities were applied manually to simulate real‐world mechanical impacts. The stand‐alone elements of the amino acid‐based multicomponent salt crystals were attached with flexible copper tapes as both top and bottom electrodes, forming an electrode‐piezoelectric layer‐electrode sandwich element, as shown in Figure 8A,B. As a proof‐of‐concept demonstration of the fabricated piezoelectric discs under realistic conditions, mechanical excitation was imparted by a single operator using four mechanical conditions: single finger (index), two fingers, three fingers, and full hand tapping stimuli. For each condition, the piezoelectric elements based on trihaloacetates of basic amino acids such as L‐histidine and L‐lysine were subjected to 20 independent trials, with each trial consisting of 10 consecutive press‐release cycles where the generated open circuit‐voltage (V_oc_) was recorded using a digital oscilloscope.

**FIGURE 8:**
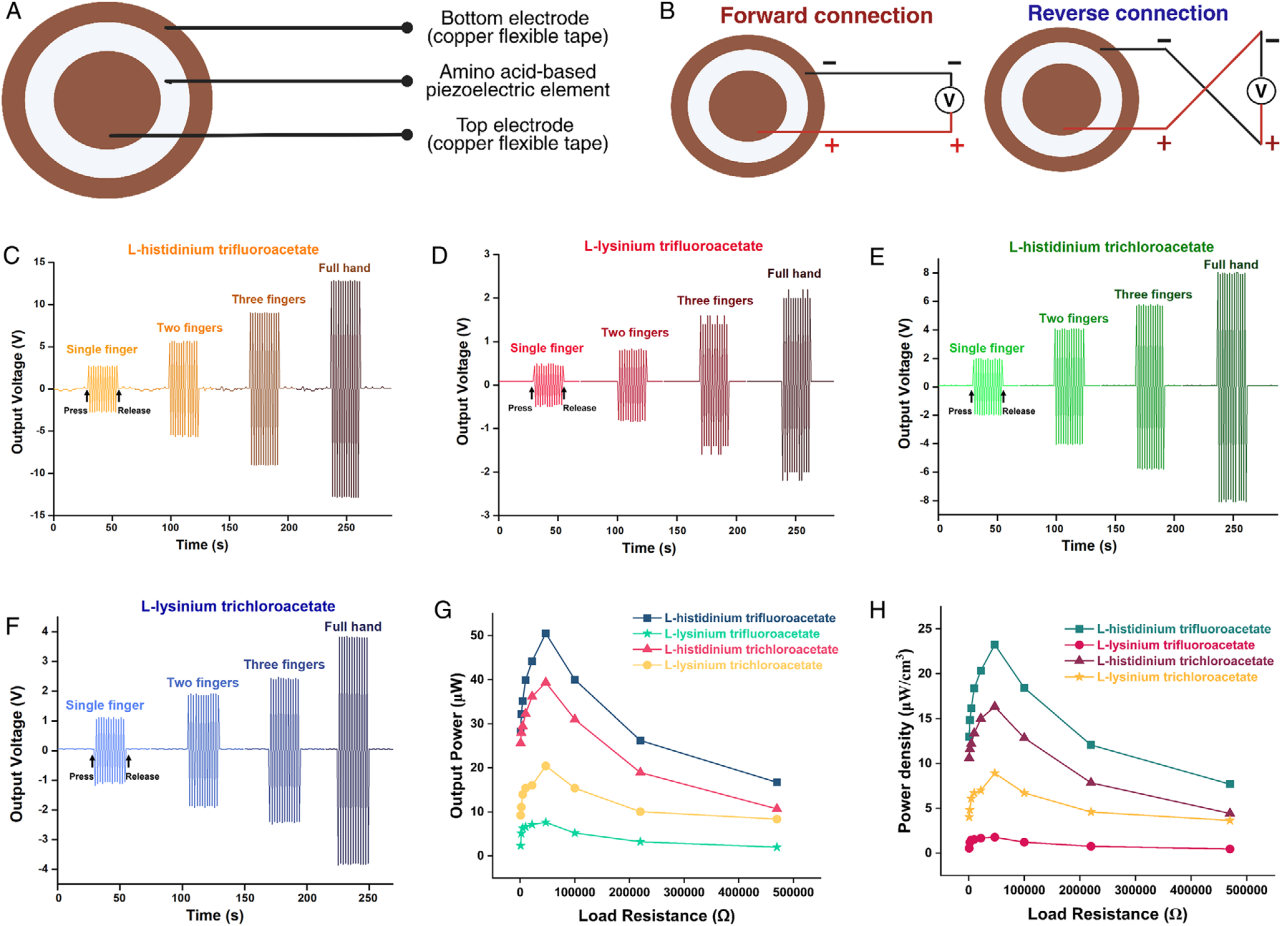
Energy harvesting studies of the amino acid trihaloacetates (A) Schematic of top‐down view of an electroded amino acid‐based piezoelectric element using flexible copper tapes. (B) Schematic of forward and reverse connection. Open circuit voltage (OCV) of (C) L‐histidinium trifluoroacetate. (D) L‐lysinium trifluoroacetate. (E) L‐histidinium trichloroacetate. (F) L‐lysinium trichloroacetate circular discs with a diameter = 3.3 cm under manual mechanical stimuli. (G) Output power obtained for the amino acid trihaloacetates. (H) Output power density for the amino acid trihaloacetates.

Figure 8C shows that a maximum open circuit voltage (OCV) of 12.86 V was obtained for L‐histidinium trifluoroacetate in forward connection under manual mechanical stimuli. To further confirm that the output performance originated from the inherent piezoelectricity of L‐histidinium trifluoroacetate, and not from other mechanisms such as electrostatic induction, parasitic capacitance, or triboelectric behavior, the reverse connection measurements were performed [78, 79]. In the reverse connection, a maximum open circuit voltage of −6.24 V was attained for L‐histidinium trifluoroacetate, as revealed in Figure S12A. Figure 8D and Figure S12B presents the voltage characteristics of the presoldered sandwiched disc of L‐lysinium trifluoroacetate delivering a maximum open circuit voltage of 2.20 and −2.00 V in forward and reverse connection, respectively. For the trichloroacetate salts, a maximum OCV of 8.08 V and was obtained when the L‐histidinium trichloroacetate sandwiched piezoelectric device with flexible copper electrodes was connected in forward connection and under reverse connection, a maximum open circuit voltage of ‐3.80 V was gained, as depicted in Figure 8E and Figure S12C. A maximum open circuit voltage of 3.86 and −1.80 V was obtained when the L‐lysinium trichloroacetate piezoelectric sandwich disc was connected in forward and reverse connection respectively, as given in Figure 8F and Figure S12D. In all the above cases, it is evident that the fabricated piezoelectric discs of amino acid‐based multicomponent salts are sensitive to even “gentle” tapping and exhibited stable electrical output under manual mechanical stimuli, demonstrating exceptional mechanical to electrical energy conversion. The output performance of the amino acid‐based multicomponent salts was compared with a commercial piezo buzzer of diameter = 3.5 cm from RS Pro (Stock No. 837–7840). The commercial piezoelectric element showed a maximum open circuit voltage of 5.96 and −6.48 V in forward and reverse connection, respectively, under manual mechanical stimuli (Figure S12E–G). A comparison of the piezoelectric coefficients and output performance of various organic piezoelectric materials is summarized in Table S20, which validates the piezoelectric performance of the amino acid‐based multicomponent salts, thus expanding new prospects for the advancement of organic piezoelectrics.

To understand the applicability of the fabricated discs of the multicomponent salts of amino acids, the short‐circuit current, output power, and output power density were measured by varying external load resistances (*R_L_*) from 1 kΩ to 10 MΩ (Figure S13). A maximum instantaneous power of 50 µW was obtained for L‐histidinium trifluoroacetate disc at an optimum load resistance of 47 kΩ (Figure 8G). Considering the disc dimensions with diameter of 3.3 cm and average thickness of 2.54 mm, the calculated maximum volumetric peak power density and maximum instantaneous peak power density of L‐histidinium trifluoroacetate disc were 23.24 µW/cm^3^ (Figure 8H) and 5.90 µW/cm^2^, respectively at an external load resistance of 47 kΩ. In the case of L‐lysinium trifluoroacetate disc of average thickness = 5.01 mm, the maximum volumetric peak power density and maximum instantaneous peak power density were calculated to be 1.77 µW/cm^3^ and 0.89 µW/cm^2^, respectively at an external load resistance of 47 kΩ. Regarding the trichloroacetates of L‐histidine (average thickness = 2.82 mm) and L‐lysine (average thickness = 2.68 mm) at an external load resistance of 47 kΩ, the obtained maximum volumetric peak power density were 16.31 and 8.91 µW/cm^3^, respectively and maximum instantaneous peak power density were calculated as 4.60 and 2.39 µW/cm^2^, respectively. We measured the average capacitance (in pF) and calculated the piezoelectric voltage constants (*g*_33_ in mV∙m/N) of some of the multicomponent salts of amino acids, as shown in Table 2. L‐histidinium trifluoroacetate discs have an average capacitance (C) of 0.53 pF, whereas the L‐lysinium trifluoroacetate discs provided an average capacitance of 0.59 pF. In the trichloroacetate salt stand‐alone elements, the average capacitance (C) of the discs was found to be 0.35 pF for L‐histidinium trichloroacetate, and for L‐lysinium trichloroacetate discs, we calculated an average capacitance of 2.21 pF. The observed low capacitance (0.35–2.21 pF) is expected for biomolecular crystals, due to their low relative permittivity revealed by both experimental measurements and computational calculations, thus providing high sensitivity for potential vibration‐based applications such as structural health monitoring, wearable, implantable medical devices, and self/low‐powered electronics [80, 81, 82]. The low capacitance of the fabricated piezoelectric elements of amino acid‐based multicomponent salts can result in high impedance bottleneck. This can be resolved by various circuit design strategies including charge amplifiers, active rectification, and synchronous electric charge extraction (SECE) rather than direct resistive or inductive matching (Note S6). Among the salt crystals, the higher relative permittivity obtained for L‐lysinium trichloroacetate experimentally is owing to the higher capacitance, even though the average thickness of all the samples was analogous (Table S21). From the average capacitance, average thickness, and average longitudinal piezoelectric response of the circular discs (Table S21), we calculated their longitudinal piezoelectric voltage constant, *g*_33_, in mV∙m/N of the multicomponent salt crystals (Table 2), using Equation (1).

(1) $$g_{33}=\frac{d_{33}}{\varepsilon_{r}\varepsilon_{0}}$$

**TABLE 2:** Experimental and predicted relative permittivity (*ε_r_*), experimental and predicted piezoelectric voltage constants (*g*_33_ in mV∙m/N), and figure of merit (*d_33_* *g_33_ in 10^−12^ m^2^/N) of the stand‐alone elements of amino acid‐based multicomponent salts.

Amino Acid‐Based Multicomponent Salts	Average Capacitance [pF]	Relative Permittivity (*ε_r_*), Experimental–Polycrystalline Circular Discs of Diameter = 3.3 cm	Relative Permittivity (*ε_r_*), Predicted–Single Crystal	*g_33_*, Experimental–Polycrystalline Circular Discs of Diameter = 3.3 cm (mV∙m/N)	*g_33_^eff^*, Predicted–Single Crystal (mV∙m/N)
*L‐histidinium trifluoroacetate*	0.53	5.76	3.26	60	163.70
*L‐lysinium trifluoroacetate*	0.59	9.19	2.4	7	58
*L‐histidinium trichloroacetate*	0.35	4.68	2.61	50	243.20
*L‐lysinium trichloroacetate*	2.21	28.10	2.61	8	94.70

The obtained piezoelectric voltage constants of L‐histidinium trifluoroacetate and L‐histidinium trichloroacetate (50 to 60 mV∙m/N) given in Table 2 are significantly higher than the commercial grade piezoelectric soft ceramics such as lead zirconate titanate (PZT‐5A) with *g_33_* ranging from 20 to 30 mV∙m/N [83] through powder processing, composite fabrication with polymers, and texturing processes, and further comparable with other lead‐free ceramics like potassium sodium niobate (KNN) with *g_33_* = 47 mV∙m/N [84, 85, 86, 87] Besides this, for practical applications, the temperature dependent effective longitudinal piezoelectric response (*d_33_^eff^*) [88] of the trihaloacetates of basic amino acids such as L‐histidine and L‐lysine offered minimal compromise in the obtained piezoelectric response for films annealed from 50 to 100°C, especially for L‐histidinium trifluoroacetate and L‐lysinium trichloroacetate discs (Note S5, Figure S14, and Tables S22–S25). Thus, these multicomponent salts based on amino acids present themselves as promising eco‐friendly alternatives in piezoelectric energy harvesting applications.

## 3 Conclusions

In this work, the successful fabrication of amino acid‐based multicomponent salts into stand‐alone piezoelectric device components was achieved by combining low energy‐intensity crystallization with computational predictions through DFT analysis. These multicomponent salt crystals of L‐histidine, L‐lysine, L‐arginine, and L‐proline offer enhanced piezoelectric performance, leading to measurable macroscopic electromechanical coupling with a maximum longitudinal piezoelectric response (*d_33_*) of 7.8 pC/N, which was confirmed using computational calculations. An exceptionally large maximum open circuit voltage of 12.9 V was obtained for L‐histidinium trifluoroacetate systems under manual tapping, highlighting their potential ease‐of‐use in consumer electronics using ambient and direct mechanical force harvesting. Other notable candidates, such as L‐lysinium trifluoroacetate, L‐histidinium trichloroacetate, and L‐lysinium trichloroacetate, exhibited reliable longitudinal piezoelectric responses and a maximum open circuit voltage of 2.2, 3.9, and 8.1 V, respectively, thus highlighting customizable properties of amino acid‐based piezoelectric materials. A maximum instantaneous power, maximum volumetric peak power density, and maximum instantaneous peak power density of L‐histidinium trifluoroacetate disc were determined to be 50 µW, 23.24 µW/cm^3^, and 5.90 µW/cm^2^, respectively at an external load resistance of 47 kΩ. By correlating the experimental outcomes with computational calculations, we have discussed the structure‐property relationships in these salt crystals in terms of the nature of coformers, intermolecular interactions, polarization, and elastic properties. Future work will include high‐throughput screenings of small‐molecule coformers with amino acids, thus extending the scope of organic piezoelectrics with a library of computationally and experimentally validated materials tailored for commercial applications in piezoelectric sensing, energy harvesting, and actuation.

## Author Contributions

**Krishna Hari**: conceptualization, investigation, writing – original draft, methodology, validation, visualization, writing – review and editing, software, formal analysis, data curation. **Shubham Vishnoi**: validation, conceptualization, investigation, writing – original draft, writing – review and editing, visualization, software, formal analysis, data curation, methodology. **Sarah Guerin**: project administration, resources, supervision, writing – review and editing, writing – original draft, funding acquisition, conceptualization.

## Conflicts of Interest

The authors declare no conflicts of interest.

## Supporting information




**Supporting File**: advs78588‐sup‐0001‐SuppMat.pdf.

## Data Availability

Experimental data, methodology, and computational data are available in the Supplementary Information (SI) document. SI includes Supplementary Notes S1–S6, Scheme S1, Figures S1–S14, and Tables S1–S25.
