# Supplementary material for: Piezoelectric Amino Acid Salts with Halogen‐Based Coformers Produce Exceptional Force‐Generated Voltage Output
Source: Adv Sci (Weinh). 2026 Nov 5:e78588. Online ahead of print. doi: 10.1002/advs.78588 (PMC13735429; doi:10.1002/advs.78588)
Supplement: Supplementary file 1 [file ADVS-9999-e78588-s001.pdf]

Supporting Information

Piezoelectric amino acid salts with halogen-based coformers produce exceptional force-generated voltage output

Krishna Hari[1], Shubham Vishnoi[1], Sarah Guerin[1, 2]*

[1]*Department of Chemical Sciences, Bernal Institute, University of Limerick, V94 T9PX, Ireland.*

[2]*SSPC, The Research Ireland Research Centre for Pharmaceuticals, University of Limerick, V94 T9PX, Ireland.*

*Correspondence to Sarah Guerin. e-mail: sarah.guerin@ul.ie

Supplementary Notes

Note S1. Experimental Methodology

Note S2. Computational Methodology and Simulation Details

Note S3. Longitudinal piezoelectric response of L-argininium trifluoroacetate, L-argininium bis(trifluoroacetate), and L-prolinium trichloroacetate

Note S4. Wide angle X-ray scattering (WAXS) measurements of L-histidinium trichloroacetate

Note S5. Temperature dependant effective longitudinal piezoelectric studies of trihaloacetates of amino acids

Note S6. Strategies to resolve high impedance of the fabricated piezoelectric amino acid trihaloacetate elements

Supplementary Scheme

Scheme S1. Piezoelectric strain matrix showing matrix components: normal, longitudinal, transverse, and shear coefficients.

Supplementary Figures

Figure S1. TGA and DSC plots of (A) L-histidinium trifluoroacetate. (B) L-lysinium trifluoroacetate. (C) L-argininium trifluoroacetate. (D) L-argininium bis(trifluoroacetate). (E) L-histidinium trichloroacetate. (F) L-lysinium trichloroacetate. (G) L-prolinium trichloroacetate.

Figure S2. Three-dimensional representations of the piezoelectric strain tensor (d_{ij}, pC/N) depict the directional dependence and magnitude of the predicted electromechanical responses (Scheme S1). The surface contours illustrate anisotropic behaviour arising from crystal symmetry and atomic arrangement, revealing how mechanical deformation induces electric polarisation along distinct crystallographic directions.

Figure S3. PFM scan showing a staircase effect of response amplitude with increase in the driving voltage of (A) L-histidinium trifluoroacetate. (B) L-histidinium trichloroacetate. (C) L-prolinium trichloroacetate.

Figure S4. Debye rings using Wide angle X-ray scattering (WAXS) measurements on (A) powdered sample with equal (albeit low intensity) intensity regardless of azimuthal angle, indicating random orientation. (B) stand-alone disc displaying bright spots of high intensity at specific azimuthal angles which signify the presence of localised region of preferred crystallite orientation of L-histidinium trichloroacetate.

Figure S5. Comparative Hirshfeld surfaces and 2D fingerprint plots of the modelled crystal series mapped onto a standardised d_{norm} scale (-0.5 to 1.5 au). (A) The spatial contact distributions and localised F---H and Cl---H interaction modes across the crystal dataset. (B) The deconstructed 2D fingerprint profiles highlight the structural transition from localised, rigid F---H paths to multi-channel, polarisable heavy-atom networks (Cl---Cl and Cl---O), alongside corresponding longitudinal piezoelectric strain values (d_{33}^{eff}).

Figure S6. Anisotropic structural coupling, energy frameworks and Hirshfeld surface analysis of L-histidinium trichloroacetate (IDAZET) in the *bc*-plane. (A) Crystallographic packing illustrating mechanical compliance along the stacking *c*-axis (C_{33}) drives column distortion under compression, projecting a transverse polarisation response along the polar *b*-axis (e_{23} = 0.27 C/m^2, d_{23} = 23.07 pC/N). (B) Predicted dispersion energy frameworks showing dominant non-covalent interactions (E_{disp} = -17.6 kJ/mol) outweighing electrostatic contributions (E_{ele} = -10.8 kJ/mol) along the soft stacking direction. Lower panels show the mapped Hirshfeld surface alongside resolved 2D fingerprint profiles quantifying close Cl---H and Cl---O contacts.

Figure S7. Plane-wave grid cutoff convergence profiles showing total raw energy across the studied crystal systems.

Figure S8. Relative density grid density convergence profiles tracking total raw energy with reference to the production baseline.

Figure S9. Electronic wavefunction optimisation convergence profiles showing the stability of total energy against tighter electronic thresholds.

Figure S10. Relative energy convergence profiles across the studied crystal systems as a function of (A) plane-wave grid cutoff. (B) relative grid density. (C) electronic wavefunction optimisation threshold. Panels show the relative energy variations (ΔE) where dashed lines indicate the selected calculation parameters.

Figure S11. Full stress-strain curves for three discs with diameter = 3.3 cm of (A) L-histidinium trifluoroacetate. (B) L-lysinium trifluoroacetate. (C) L-histidinium trichloroacetate. (D) L-lysinium trichloroacetate.

Figure S12. Energy harvesting studies of the amino acid trihaloacetates in reverse connection (A) L-histidinium trifluoroacetate. (B) L-lysinium trifluoroacetate. (C) L-histidinium trichloroacetate. (D) L-lysinium trichloroacetate (E) Photograph of the commercial piezo buzzer of diameter = 3.5 cm from RS Pro (Stock No. 837-7840). Open circuit voltage (OCV) of the commercial piezo buzzer in (F) Forward connection. (G) Reverse connection.

Figure S13. Voltage and current plots of trihaloacetates of amino acids (A) L-histidinium trifluoroacetate. (B) L-lysinium trifluoroacetate. (C) L-histidinium trichloroacetate. (D) L-histidinium trichloroacetate.

Figure S14. Temperature dependant d_{33}^{eff} studies of the multicomponent salts based on amino acids (A) L-histidinium trifluoroacetate. (B) L-lysinium trifluoroacetate. (C) L-histidinium trichloroacetate. (D) L-lysinium trichloroacetate.

Supplementary Tables

Table S1. Thickness measurements of L-argininium trifluoroacetate discs with a diameter = 3.3 cm using digital vernier callipers. Units were in mm.

Table S2. Local piezoelectric measurements of L-argininium trifluoroacetate discs with a diameter = 3.3 cm. Units were pC/N.

Table S3. Thickness measurements of L-argininium bis(trifluoroacetate) discs with a diameter = 3.3 cm using digital vernier callipers. Units were in mm.

Table S4. Local piezoelectric measurements of L-argininium bis(trifluoroacetate) discs with a diameter = 3.3 cm. Units were pC/N.

Table S5. Structural information of the studied multicomponent systems, including crystal system, unit cell content, and space group information.

Table S6. Thickness measurements of L-histidinium trifluoroacetate discs with a diameter = 3.3 cm using digital vernier callipers. Units were in mm.

Table S7. Local piezoelectric measurements of L-histidinium trifluoroacetate discs with a diameter = 3.3 cm. Units were pC/N.

Table S8. Thickness measurements of L-lysinium trifluoroacetate discs with a diameter = 3.3 cm using digital vernier callipers. Units were in mm.

Table S9. Local piezoelectric measurements of L-lysinium trifluoroacetate discs with a diameter = 3.3 cm. Units were pC/N.

Table S10. Thickness measurements of L-histidinium trichloroacetate discs with a diameter = 3.3 cm using digital vernier callipers. Units were in mm.

Table S11. Local piezoelectric measurements of L-histidinium trichloroacetate discs with a diameter = 3.3 cm. Units were pC/N.

Table S12. Thickness measurements of L-lysinium trichloroacetate discs with a diameter = 3.3 cm using digital vernier callipers. Units were in mm.

Table S13. Local piezoelectric measurements of L-lysinium trichloroacetate discs with a diameter = 3.3 cm. Units were pC/N.

Table S14. Quantitative analysis (degree of coherency) of local crystallite orientation performed on SEM micrographs of amino acid trihaloacetates using the OrientationJ Measure plugin available in ImageJ.

Table S15. Intermolecular contact percentages contributions derived from 2D Hirshfeld fingerprint plots alongside predicted longitudinal piezoelectric coefficients (d_{33}^{eff}) for the modelled crystal series.

Table S16. Computed piezoelectric coefficients (d_{ij}) and predicted hydrogen-bond counts for the multicomponent systems.

Table S17. Calculated piezoelectric strain tensor components (d_{ij}, pC/N) for studied amino acid-based multicomponent salts, alongside their corresponding space groups, the full elastic tensor components (C_{ij}, GPa), Piezoelectric Charge Constants (C/m^2), Dielectric Constants, and Piezoelectric Voltage Constants (g_{ij}, mV·m/N). This table provides a complete dataset linking directional mechanical properties to electromechanical performance, enabling detailed analysis of structure-property relationships and anisotropic piezoelectric response in these systems.

Table S18. Dipole moments of relaxed crystal structures calculated using CP2K. Each component (X, Y, Z) and the total dipole are obtained from the Berry phase formalism, which captures the collective electronic polarisation of the unit cell. The values reflect the macroscopic dipole of the crystal and generally differ from a simple sum of individual molecular dipoles due to periodicity, electronic delocalisation, and molecular orientation within the lattice.

Table S19. Comparison of the Young's modulus (GPa) of various organic piezoelectric and commercial materials.

Table S20. Comparison of the piezoelectric coefficients (pC/N or pm/V) and output performance (V) of various organic piezoelectric materials. Output performance is given as either Voc is the output circuit voltage or Vp-p is the peak-to-peak voltage.

Table S21. Calculations of longitudinal piezoelectric voltage constant g_{33} (mV·m/N) from average capacitance, average thickness, and average piezoelectric response of the circular discs of diameter = 3.3 cm of the multicomponent systems.

Table S22. Temperature dependant longitudinal piezoelectric studies of L-histidinium trifluoroacetate discs annealed for 24 hrs at different temperature ranging from 50 °C to 100 °C.

Table S23. Temperature dependant longitudinal piezoelectric studies of L-lysinium trifluoroacetate discs annealed for 24 hrs at different temperature ranging from 50 °C to 100 °C.

Table S24. Temperature dependant longitudinal piezoelectric studies of L-histidinium trichloroacetate discs annealed for 24 hrs at different temperature ranging from 50 °C to 100 °C.

Table S25. Temperature dependant longitudinal piezoelectric studies of L-lysinium trichloroacetate discs annealed for 24 hrs at different temperature ranging from 50 °C to 100 °C.

Supplementary Notes

S1. Experimental Methodology

Materials

L-histidine (98%, Fischer Scientific Ltd.), L-lysine (98%, Fischer Scientific Ltd.), L-arginine (98%, Fischer Scientific Ltd.), L-proline (99%, Thermo Scientific Chemicals), Trifluoroacetic acid (99%, Thermo Scientific Chemicals), and Trichloroacetic acid (99%, Thermo Scientific Chemicals) were purchased and used without any purification.

Methods

Crystal Growth: For 1:1 and 1:2 ratios of amino acid: trihaloacetic acids, trihaloacetic acids were dissolved in a minimum amount of deionised (DI) water, and the desired amino acids were slowly added to this solution. In all cases, the solution was stirred for about 30 minutes until it became clear and homogeneous. The solution was then poured into the silicone molds (dimensions: circular - 3.3 cm; volume = 4 mL) and was left to evaporate at ambient conditions for 5 to 7 days. After air drying, the discs were taken out by gently pushing the silicone molds from underneath.

Powder X-ray Diffraction (PXRD): Powder X-ray patterns were recorded using an Anton Paar XRDynamic 500 diffractometer with Cu Kα radiation (1.54 Å, 40 kV, 40 mV). Diffraction patterns were collected in θ-2θ configuration at room temperature in the range 4-60° using a step size of 0.002°, and the obtained patterns were compared with the reported structures in the Cambridge Structural Database (CSD).

Scanning Electron Microscopy (SEM): SEM images were taken on a Hitachi SU-70. Crystals were coated in a 5 nm gold film before imaging, using a K550 Emitech Sputter Coater, with a coating current of 20 mA to prevent high-energy electron beam damage of the multicomponent polycrystalline assemblies. To characterise the crystallite orientation and degree of coherency thereby correlating them with the obtained longitudinal piezoelectric response (d_{33}^{eff}) of trihaloacetates of amino acids, SEM micrographs were analysed using ImageJ plugin "OrientationJ Measure" [1, 2]. OrientationJ Measure, an open source plugin, is used to quantitatively determine the orientation and isotropy properties of a region of interest in an image and evaluate for every pixel of the image based on the structure tensor using a specified Gaussian shaped window. [3]

Macroscale Piezoelectric Measurements: Piezoelectric measurements were carried out using a commercial d_{33} piezometer from PiezoTest with a numerical accuracy of 0.01 pC/N. This measurement setup is based on the Berlincourt method, with the polycrystalline assemblies placed between two electrodes, securely clamped and subjected to a dynamic force of 0.25 N and a static force of 2 N at 110 Hz. Further, the sample was inverted along the chosen axis to measure the opposite polarity and confirm a genuine longitudinal response. Lead Zirconium Titanate references were used for calibration.

Piezoresponse Force Microscopy (PFM): Piezoresponse force microscopy (PFM) was carried out on an NX10 atomic force microscope (AFM) from Park Systems (Suwon, South Korea) equipped with an ElectriMulti75-G probe from Budget Sensors (Sofia, Bulgaria). PFM measurements were carried out with the conductive probe in contact with the surface whilst an alternating current (AC) was applied to the probe. The amplitude of the probe in response to the piezoelectric effect of the sample was monitored. To determine the piezoelectric coefficient, the driving amplitude of the AC signal was increased in steps: 0 V, 2.5 V, 5 V, 7.5 V, and 10 V whilst scanning an area of 2x2 µm. Scans were performed at a scan rate of 0.05 Hz line rate and at a pixel resolution of 256x256. A staircase effect can be seen in the amplitude signal, representing an increase in the response amplitude with respect to an increase in the driving amplitude. An average response amplitude was then determined for each step and then plotted against the driving voltage. The relationship between response amplitude and driving amplitude was linear, and the piezoelectric response coefficient was taken to be the slope of the fitted line.

Thermogravimetric Analysis (TGA): Thermogravimetric analyses (TGA) were carried out in Perkin Elmer TGA 4000, between 30 °C and 500 °C, with a constant heating rate of 10 ºC/min.

Differential Scanning Calorimetry (DSC): Differential Scanning Calorimetry (DSC) was carried out in a Netzsch Polyma 214 between 30 °C and 300 °C, with a constant heating rate of 10 ºC/min.

Open circuit voltage (OCV) measurements: For the open circuit voltage measurements of the amino acid polycrystalline assemblies, the top and bottom electrodes were chosen to be flexible copper substrates (adhesive tape, RS electronics) with a diameter of 3.3 cm. Isopropyl alcohol (IPA) was used for cleaning purposes, and a smooth layer of rosin flux was used to ensure smooth soldering and wettability. For the electrical connections, AWG 28 copper wires were attached to the crystals carefully using a lead-free solder. The RS Pro Digital Storage

Oscilloscope IDS-1072B (10:1 attenuation) was connected in parallel with respect to multicomponent salts of amino acid piezoelectric elements via soldered wires and copper foils were used as top and bottom electrodes and connected to the oscilloscope via crocodile clips. Before electroding, both top and bottom electrodes were short-circuited to ground to drain any accumulated/stray charges to avoid any spurious signal contributions. This was maintained for a few seconds before taking any OCV readings. This was to assure the repeatability of the obtained results for all the samples and further voltages readings were depended upon the manual hand tapping with a frequency range of measurement of about 2-6 Hz. The polycrystalline assembly was then adhered between these pre-soldered flexible copper substrates. The oscilloscope probe provides an input resistance of 10 MΩ in parallel with an input capacitance of approximately 12 pF, which is well within the acceptable range for characterizing high impedance piezoelectric elements that typically exhibit only a few pF of intrinsic capacitance. To avoid the contribution of triboelectric effects, all the samples were reversed afterwards, and OCV measurements were taken again to check for the triboelectric contributions. During reverse connection, a clear reversal polarity was observed with consistent voltage readings similar ranges to forward connection readings.

Compression testing: A Mecmesin Multi-test 2.5 dv (Mecmesin, West Sussex, UK) device was used for compression testing to compare the strength of the fabricated discs (diameter = 3.3 cm) at the macroscale. A sample presser of diameter = 2 cm was used, and the sample height was set to an average of 4.72 mm. The strain was varied from 0 – 25% during the tests. The discs were placed rough side facing upwards in a plastic petri dish and the loader was applied uniaxially downwards at a steady rate of 1 mm/min. The apparent Young's modulus was calculated from the slope of the linear elastic region of the stress-strain curve.

S2. Computational Methodology and Simulation Details

To explore the piezoelectric properties of crystallised amino acid-based multicomponent molecular salt crystals, we used Density Functional Theory (DFT) calculations. These calculations determine polarisation within the crystal unit cell, which gives rise to dipole moments and piezoelectric effects[4]. All modelling was conducted using the CP2K package for periodic DFT calculations[5]. The Orbital Transformation (OT) SCF algorithm, combined with the Iterative Refinement of the Approximated Coupled Kohn-Sham (IRAC) method, was employed[6]. Goedecker-Teter-Hutter (GTH) pseudopotentials and a molecularly optimised double-zeta Gaussian basis set, comparable to 6-31G**, were utilised[7]. The cut-offs for plane

waves and Gaussians were 900 Ry and 60 Ry, respectively, and the energy was converged to 10^{-6} Hartree. Exchange-correlation effects were treated using the Perdew, Burke, and Ernzerhof (PBE) implementation of the Generalised Gradient Approximation (GGA) [8, 9]. Grimme D3 van der Waals corrections were also used[10, 11]. All crystal structures were optimised using conjugate gradient minimisation with a supercell model[12]. The piezoelectric and elastic tensors were calculated using a finite-difference method, with the supercell strained in each Voigt direction by ±0.015. The elastic constants are represented in the form of the stiffness tensor, *C*, as a 6×6 matrix. The piezoelectric tensor was then the response of polarisation, taken here as a periodically corrected Berry phase, to the applied strain, and the elastic tensor as the response of the stress tensor. The matrix product between the piezoelectric charge tensor, *e*, and the inverse of the elastic tensor (compliance tensor $S = C^{-1}$) gives the piezoelectric strain tensor *d (*in *pC/N* or equivalent converse piezoelectric units *pm/V)*. All piezoelectric responses are described by a third-rank tensor in the form of a 3×6 matrix (see **eq. 1** and the matrices (**eq. 2**)). Crystal dipole moments were computed using the Berry phase approach as implemented in CP2K, following the methodology reported[13].

$$d_{ik} = \frac{e_{ij}}{c_{kj}} \tag{1}$$

$$\begin{bmatrix} d_{11} & d_{12} & d_{13} & d_{14} & d_{15} & d_{16} \\ d_{21} & d_{22} & d_{23} & d_{24} & d_{25} & d_{26} \\ d_{31} & d_{32} & d_{33} & d_{34} & d_{35} & d_{36} \end{bmatrix} \tag{2}$$

For the chiral tetragonal space group systems (glycinium trichloroacetate and guanidinium trichloroacetate) , the finite-difference stress derivative evaluation can under-perturb non-local shear variables, occasionally yielding an unphysical near-zero C_{66} shear component due to directional symmetry-breaking boundaries[14,15]. To satisfy the generalized Born stability criteria and ensure mechanical equilibrium, the independent in-plane shear component was mapped using the standard in-plane isotropic shear approximation ($C_{66} = \frac{C_{11}-C_{12}}{2}$). This correction resolves the numerical symmetry artifact to provide the physically stable compliance ($S = C^{-1}$) and piezoelectric strain matrices presented in the final dataset.

Strain Tensor analysis**:** The piezoelectric strain tensor is represented by a 3×6 matrix in Voigt notation (**eq. 1** and **Scheme S1**), where the coefficients d_{11}, d_{22}, and d_{33} correspond to longitudinal piezoelectricity (polarisation along the same direction as strain), d_{12}, d_{13}, d_{21}, d_{23}, d_{31}, and d_{32} represent transverse piezoelectricity (polarisation perpendicular to the strain), and d_{14}, d_{15}, d_{16}, d_{24}, d_{25}, d_{26}, d_{34}, d_{35}, and d_{36} describe shear piezoelectricity (shear strains inducing

polarisation in different directions). This tensor effectively captures the coupling between mechanical strain and electric polarisation in piezoelectric materials. The number and type of non-zero elements in the elastic and piezoelectric tensors depend on crystal symmetry, with stiffness (C_{ij}) and compliance matrices used to derive directional mechanical properties such as Young's and shear moduli[14]. The longitudinal (C_{11}-C_{33}) and shear (C_{44}-C_{66}) stiffness components define the material's anisotropic response, with the moduli calculated *via* Voigt-Reuss-Hill averaging.

As noted, because the tetragonal point group 4 symmetry dictates strict internal symmetry constraints where $e_{31} = e_{32}$, $C_{11} = C_{22}$, $C_{13} = C_{23}$ and $C_{16} = -C_{26}$, the matrix multiplication rule cancels out any dependence on C_{66} when evaluating the third row (d_{3j}) of the piezoelectric strain tensor. Consequently, while resolving the C_{66} shear modulus to its effective ground-state approximation is mathematically mandatory to fix the mechanical stability criteria and update the Voigt-Reuss-Hill elastic averages, it leaves the longitudinal out-of-plane piezoelectric constants completely invariant (**Table S17**).

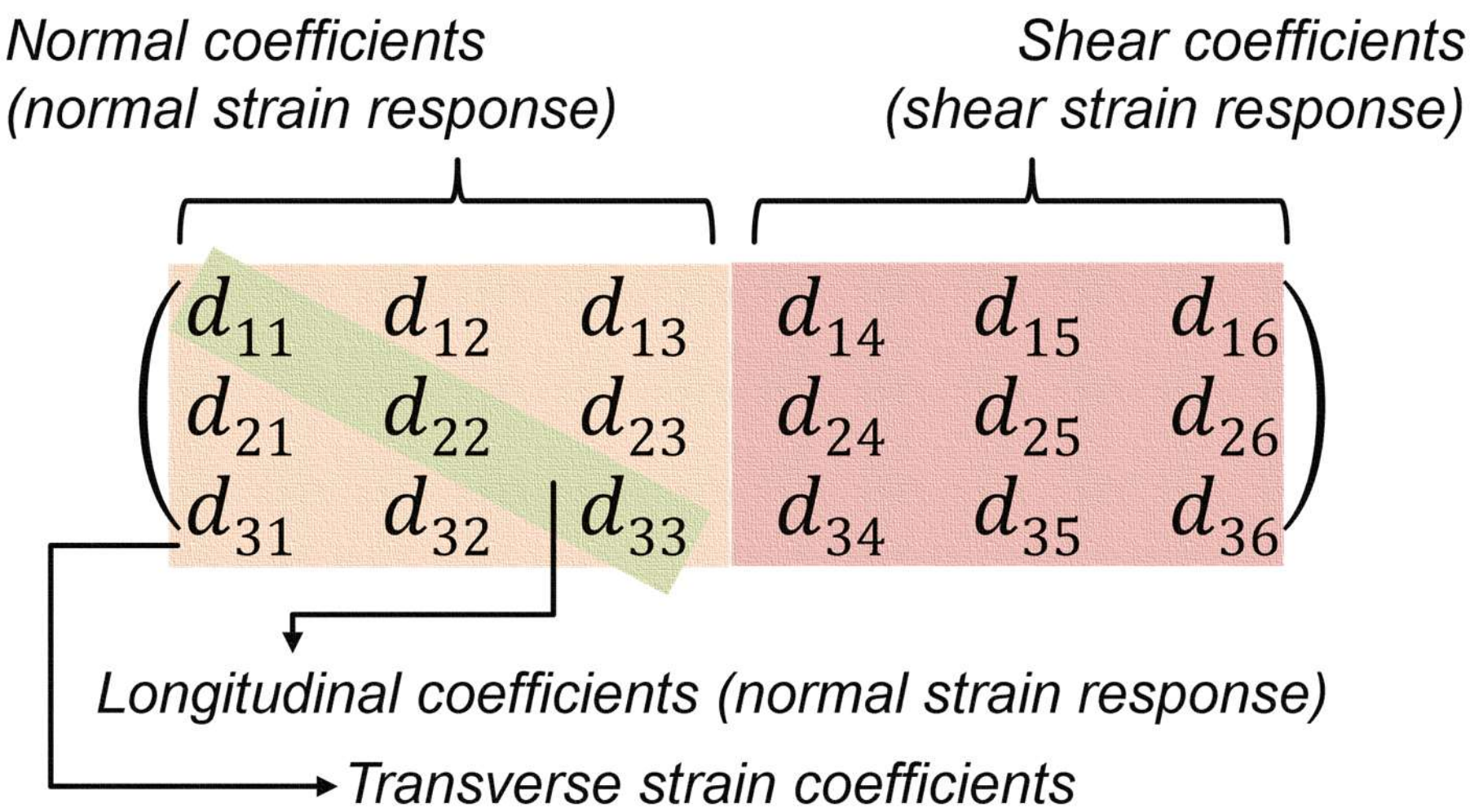

Scheme S1. Piezoelectric strain matrix showing matrix components: normal, longitudinal, transverse, and shear coefficients[4].

***Hirshfeld Surface Analysis**:* The Hirshfeld surface analysis was conducted to visualise intermolecular interactions within the crystal structure. Hirshfeld surfaces were generated using CrystalExplorer software [15]. The d_{norm} was calculated using the van der Waals radii of the atoms, as defined in **eq. 3**:

$$d_{norm} = \frac{d_i - r_i^{vdW}}{r_i^{vdW}} + \frac{d_e - r_e^{vdW}}{r_e^{vdW}} \quad (3)$$

where d_i and d_e are the distances to the nearest atom inside and outside the surface, and r_i^{vdW} and r_e^{vdW} represent the van der Waals radii of the internal and external atoms, respectively. Hirshfeld surface maps were utilised to visualise electrostatic potentials, providing insights into the nature and strength of intermolecular contacts. These analyses are complemented by *energy frameworks* computed using the B3LYP/6-31G(d,p) energy model, which visualise electrostatic and dispersion energies, providing additional insight into directional mechanical and packing stability within the L-histidinium trichloroacetate crystal with a large transverse coupling coefficient of d_{23} = 23.07 pC/N.

S3. Longitudinal piezoelectric response of L-argininium trifluoroacetate, L-argininium bis(trifluoroacetate), and L-prolinium trichloroacetate

L-argininium trifluoroacetate (ADIHOL) demonstrates a macroscopic longitudinal response of d_{33} = 3.5 pC/N and DFT predictions of d_{22} = 4.3 pC/N. L-argininium bis(trifluoroacetate) shows a moderate experimental d_{33} of 4.3 pC/N, with DFT predicting the directional components (d_{22} = 5.9 and 6.4 pC/N for two experimentally reported crystal structures, INEFIQ and INEFIQ01, respectively), reflecting the sensitivity of the computed response to unit-cell orientation and the presence of multiple trihaloacetate units, enhancing overall polarisation. These differences likely arise from factors not captured in the idealised periodic calculations, including grain boundaries, defects, domain orientations, and polycrystalline averaging. The maximum longitudinal piezoelectric response obtained for L-prolinium trichloroacetate formed as prismatic single-crystal type crystals was measured to be 7.80 pC/N (upright) and -7.35 pC/N (inverted).

S4. Wide angle X-ray scattering (WAXS) measurements of L-histidinium trichloroacetate

Wide angle X-ray scattering (WAXS) measurements were performed using Anton Paar SAXSpoint 700, with beam spot of about 500*500 um, on both powdered sample and stand-alone disc of L-histidinium trichloroacetate. The powdered sample of L-histidinium trichloroacetate exhibited Debye rings with equal (albeit low intensity) intensity regardless of azimuthal angle, indicating random orientation (**Figure S4A**). In contrast, the stand-alone element of L-histidinium trichloroacetate displayed bright spots of high intensity at specific azimuthal angles which signify the presence of localised region of preferred crystallite orientation (**Figure S4B**). However, WAXS measurements could not be reliably extended to other trihaloacetates of amino acids investigated in this work due to their surface roughness that introduced significant beam scattering artifacts and inconsistencies in the obtained

intensities. Surface polishing of the samples was not feasible without risking structural damage to the piezoelectric elements of the reported amino acid trihaloacetates. Given these constraints, we employed SEM micrograph-based orientation analysis by using ImageJ software "OrientationJ Measure" plugin as an alternative approach for all the samples investigated in this work, but only locally resolved and image-derived crystallite orientation studies can be obtained using OrientationJ Measure rather than bulk crystallographic orientation.

S5. Temperature dependant effective longitudinal piezoelectric studies of trihaloacetates of amino acids

To evaluate the temperature dependant effective longitudinal piezoelectric response (d_{33}^{eff}) of the fabricated stand-alone elements of trihaloacetates of basic amino acids such as L-histidine and L-lysine, discs of diameter = 3.3 cm were annealed at different temperatures in a convection oven for 24 hrs. **Figure S14** presents the obtained effective longitudinal piezoelectric response (d_{33}^{eff}) plotted against the temperature (50 °C - 100 °C) where minimal compromise in d_{33}^{eff} was attained for both L-histidinium trifluoroacetate and L-lysinium trichloroacetate discs (**Table S22-S25**). We note that this temperature dependant piezoelectric measurements are not substitute for humidity dependant piezoelectric studies, but systematic measurements under varying relative humidity (RH) environments were outside the scope of the available experimental facilities.

S6. Strategies to resolve high impedance of the fabricated piezoelectric amino acid trihaloacetate elements

A few circuit design strategies are provided here as suitable solutions for resolving high impedance issue due to low capacitance of the fabricated piezoelectric amino acid elements. A charge amplifier front end, built around a low-input-bias-current operational amplifier, converts the high impedance signal into usable voltage without loading the source[17]. At lower operational frequencies (~ Hz to few kHz), we advise employing synchronous electric charge extraction (SECE) to enhance efficiencies of above 80% in low powered piezoelectric microelectromechanical systems (MEMS) at similar capacitance and power scales[18]. Also, since the current obtained from these low capacitance piezoelectric devices are in the range of nanoampere to microampere range, an active rectification will aid in minimising this loss in low powered piezoelectric devices[19]. At higher operating frequencies in the range of few hundreds of kHz, a frequency dependant direct inductive (LC) resonant matching is advised[20, 21].

Supplementary Figures

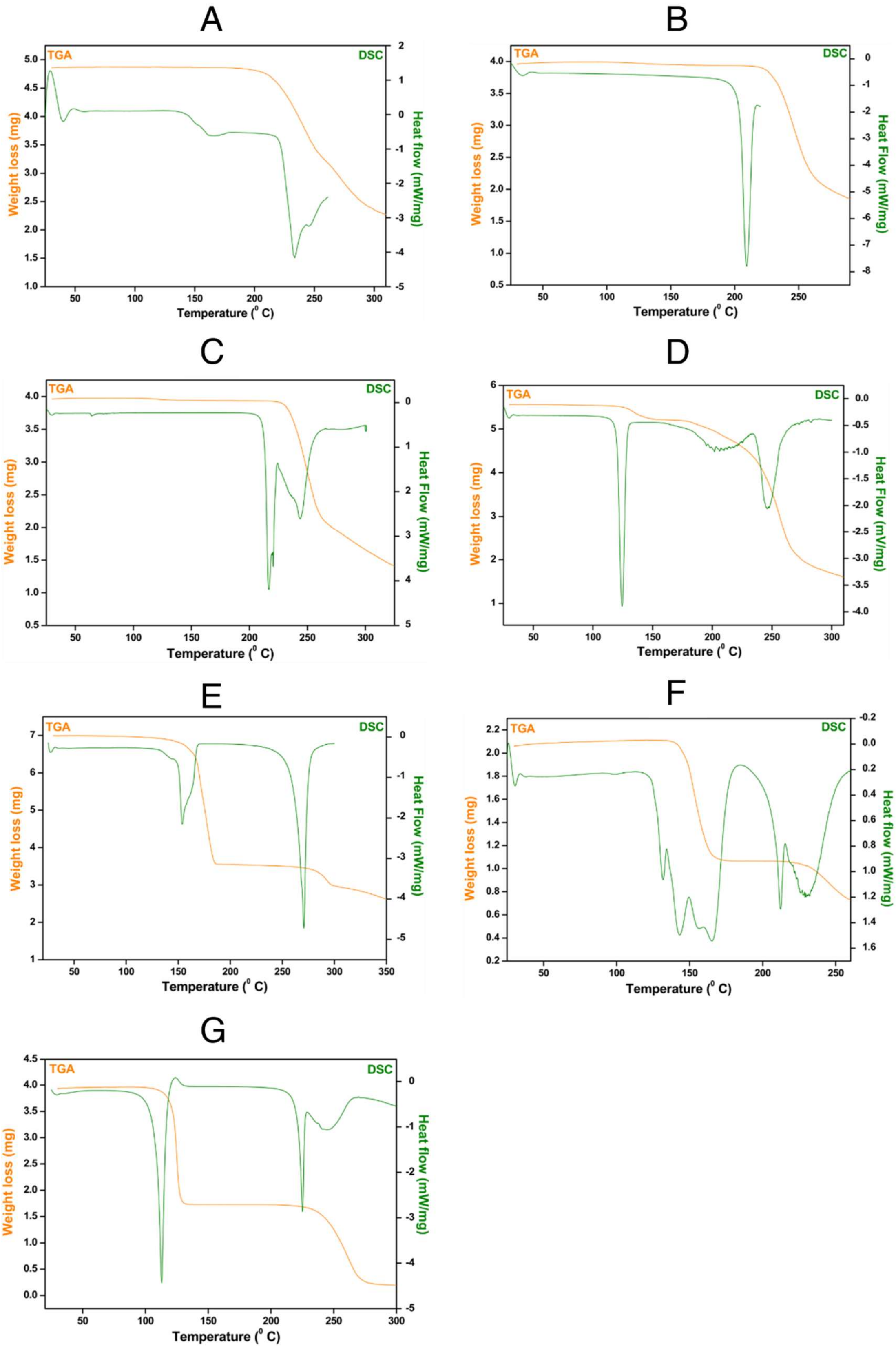

Figure S1. TGA and DSC plots of (A) L-histidinium trifluoroacetate. (B) L-lysinium trifluoroacetate. (C) L-argininium trifluoroacetate. (D) L-argininium bis(trifluoroacetate). (E) L-histidinium trichloroacetate. (F) L-lysinium trichloroacetate. (G) L-prolinium trichloroacetate.

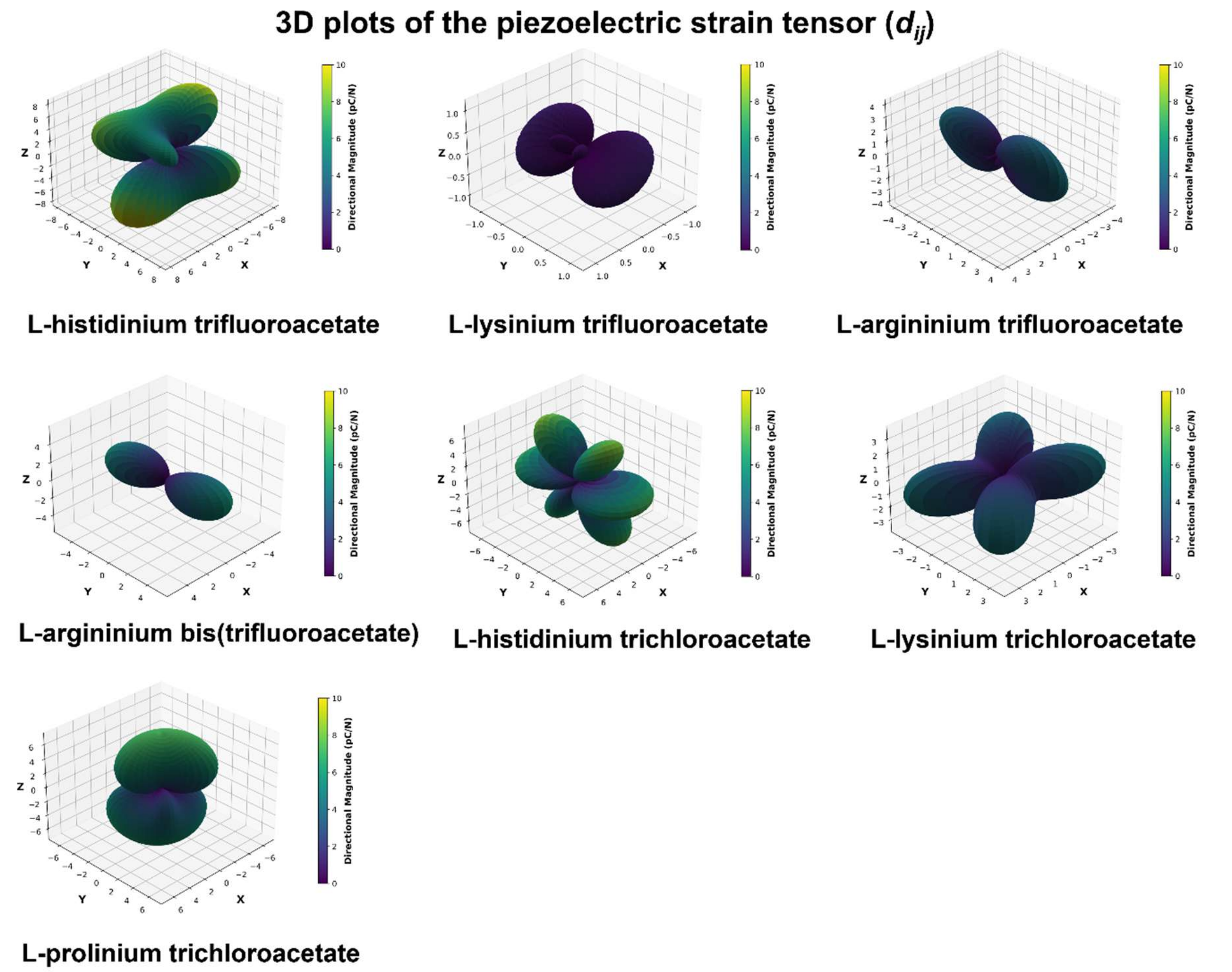

Figure S2. Three-dimensional representations of the piezoelectric strain tensor (d_{ij}, pC/N) depict the directional dependence and magnitude of the predicted electromechanical responses (Scheme S1). The surface contours illustrate anisotropic behaviour arising from crystal symmetry and atomic arrangement, revealing how mechanical deformation induces electric polarisation along distinct crystallographic directions.

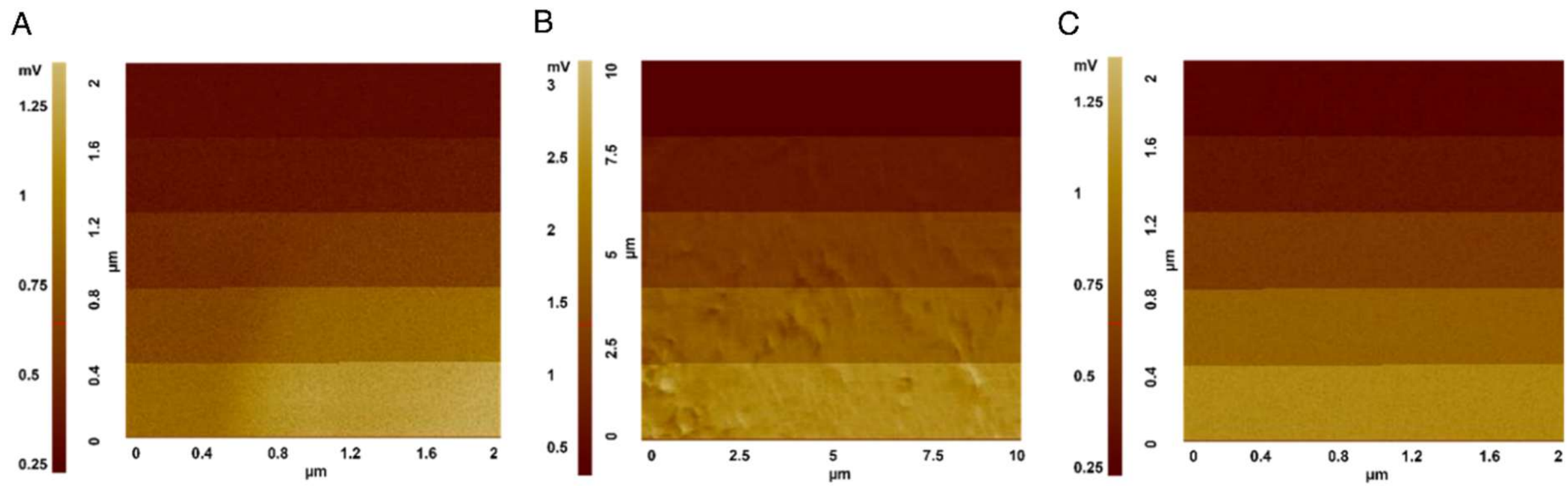

Figure S3. PFM scan showing a staircase effect of response amplitude with increase in the driving voltage of (A) L-histidinium trifluoroacetate. (B) L-histidinium trichloroacetate. (C) L-prolinium trichloroacetate.

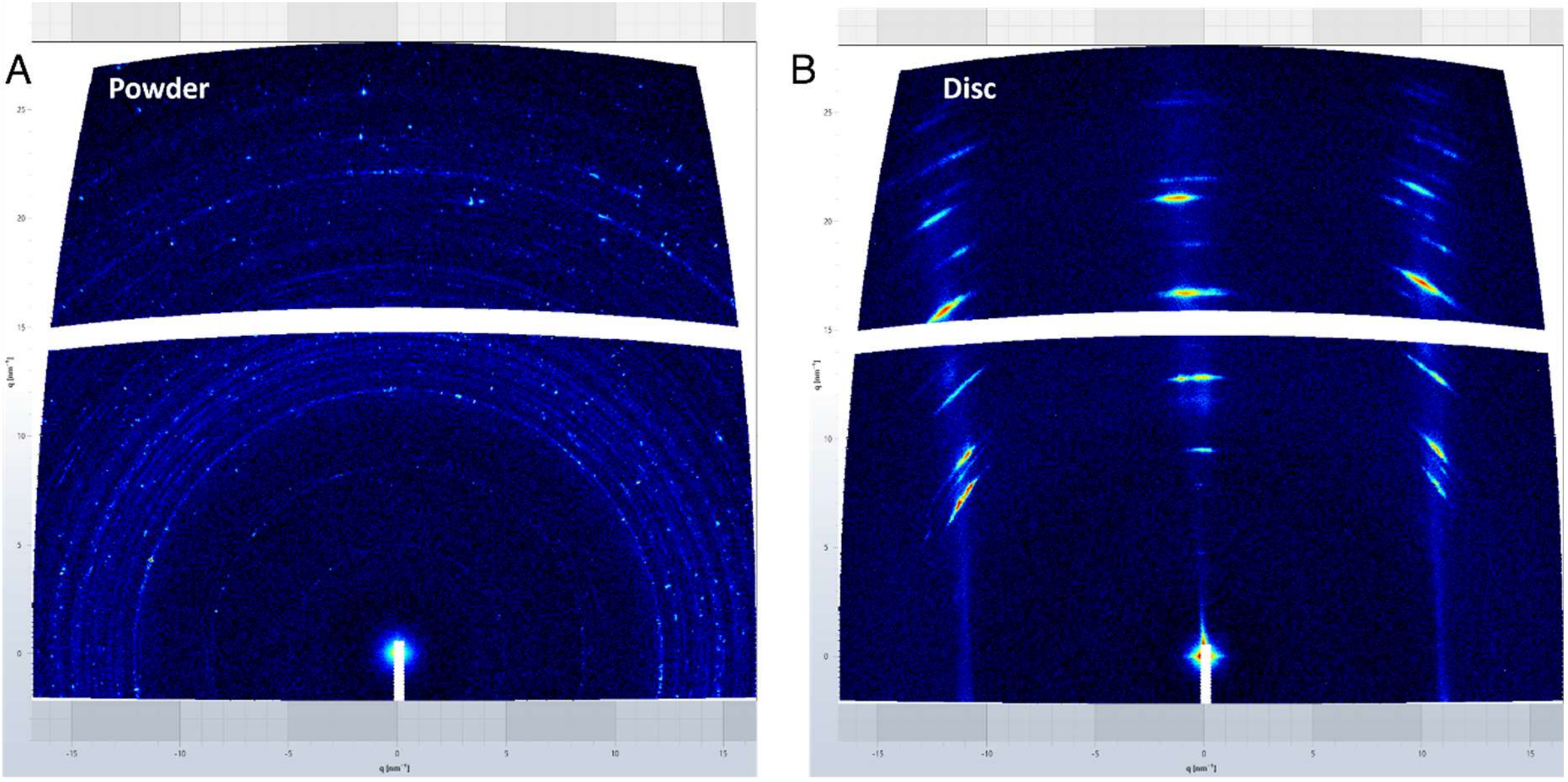

Figure S4. Debye rings using Wide angle X-ray scattering (WAXS) measurements on (A) powdered sample with equal (albeit low intensity) intensity regardless of azimuthal angle, indicating random orientation. (B) stand-alone disc displaying bright spots of high intensity at specific azimuthal angles which signify the presence of localised region of preferred crystallite orientation of L-histidinium trichloroacetate.

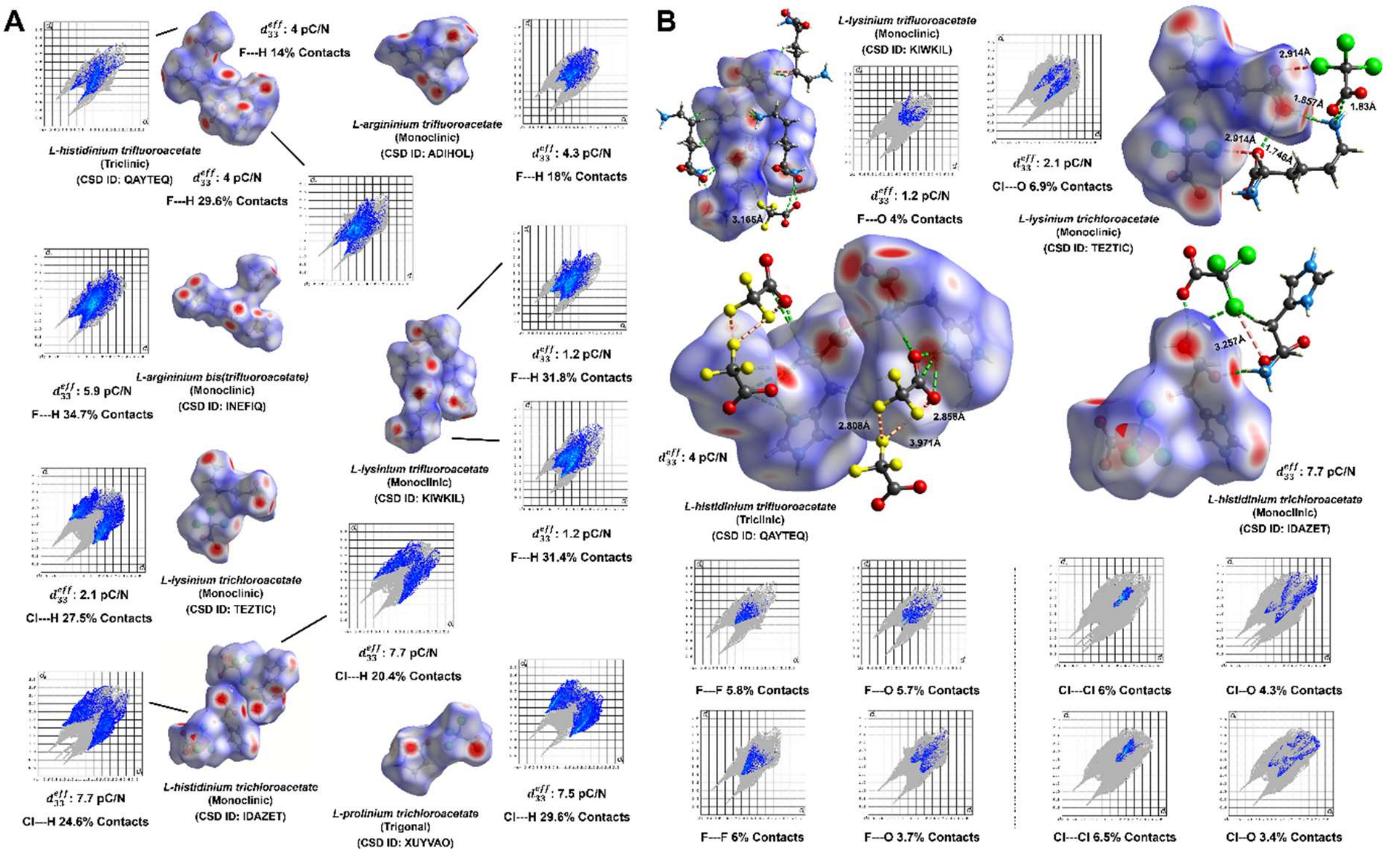

Figure S5. Comparative Hirshfeld surfaces and 2D fingerprint plots of the modelled crystal series mapped onto a standardised d_{norm} scale (-0.5 to 1.5 au). (A) The spatial contact distributions and localised F---H and Cl---H interaction modes across the crystal dataset. (B) The deconstructed 2D fingerprint profiles highlight the structural transition from localised, rigid F---H paths to multi-channel, polarisable heavy-atom networks (Cl---Cl and Cl---O), alongside corresponding longitudinal piezoelectric strain values (d_{33}^{eff}).

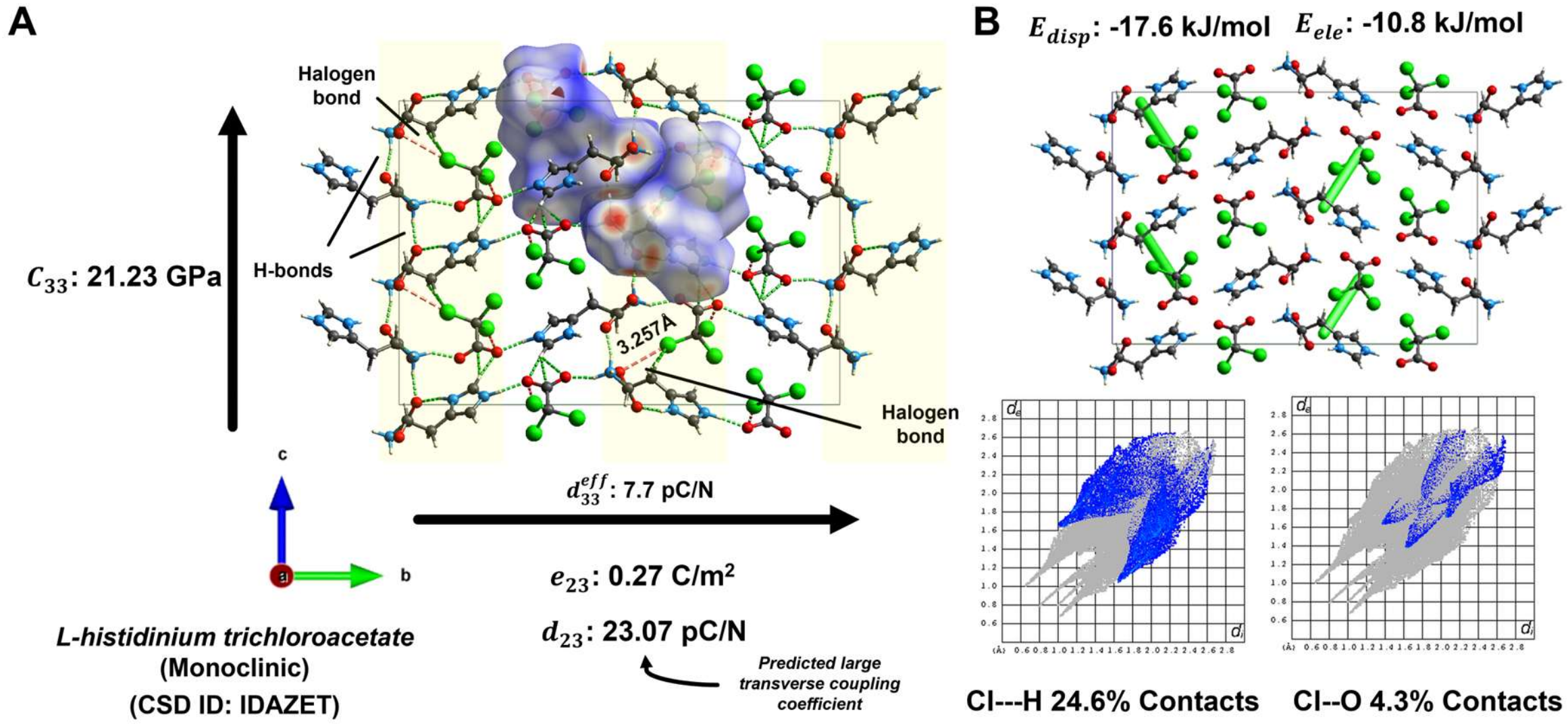

Figure S6. Anisotropic structural coupling, energy frameworks and Hirshfeld surface analysis of L-histidinium trichloroacetate (IDAZET) in the *bc*-plane. (A) Crystallographic packing illustrating mechanical compliance along the stacking *c*-axis (C_{33}) drives column distortion under compression, projecting a transverse polarisation response along the polar *b*-axis (e_{23} = 0.27 C/m², d_{23} = 23.07 pC/N). (B) Predicted dispersion energy frameworks showing dominant non-covalent interactions (E_{disp} = -17.6 kJ/mol) outweighing electrostatic contributions (E_{ele} = -10.8 kJ/mol) along the soft stacking direction. Lower panels show the mapped Hirshfeld surface alongside resolved 2D fingerprint profiles quantifying close Cl---H and Cl---O contacts.

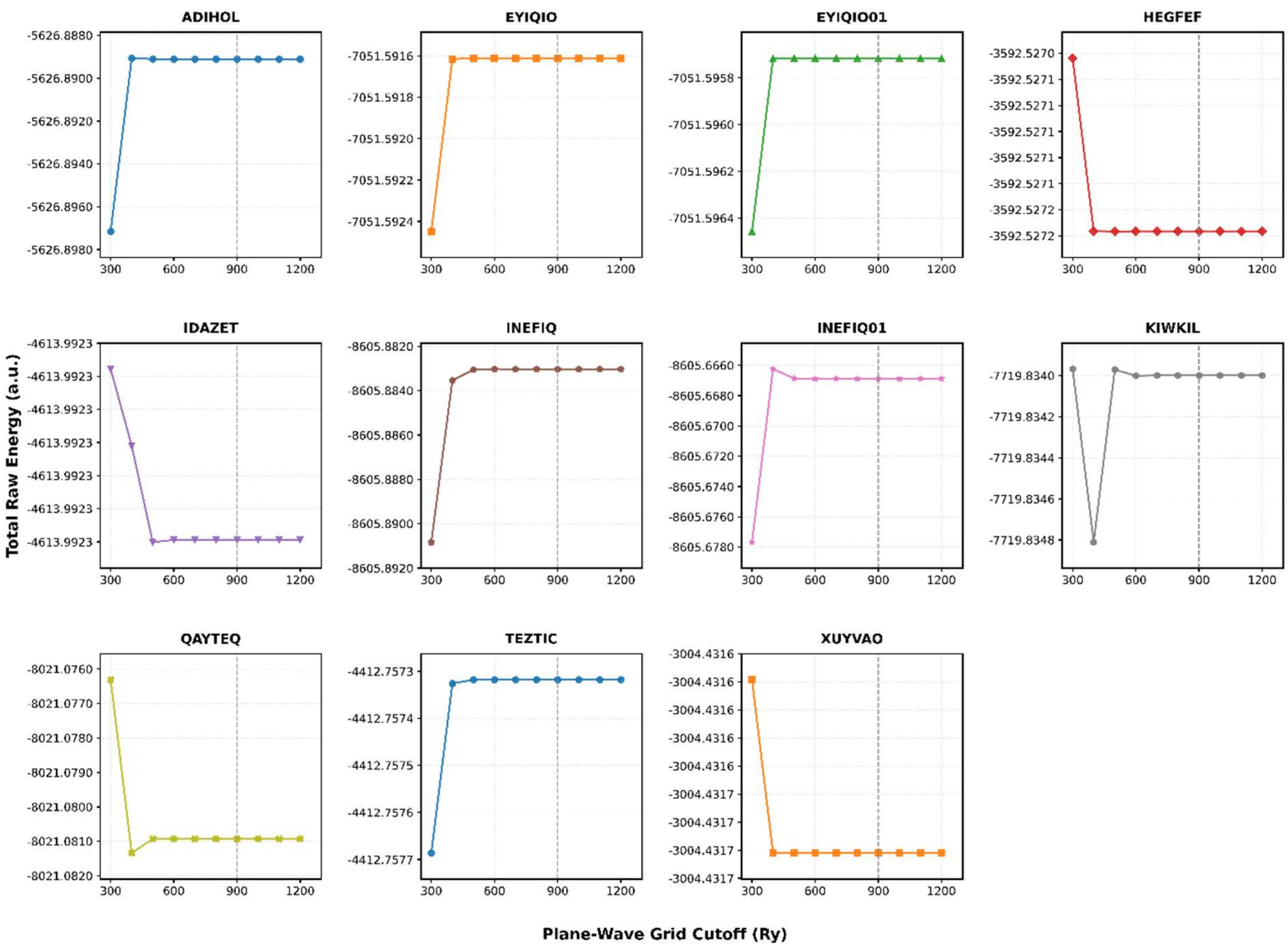

Figure S7. Plane-wave grid cutoff convergence profiles showing total raw energy across the studied crystal systems.

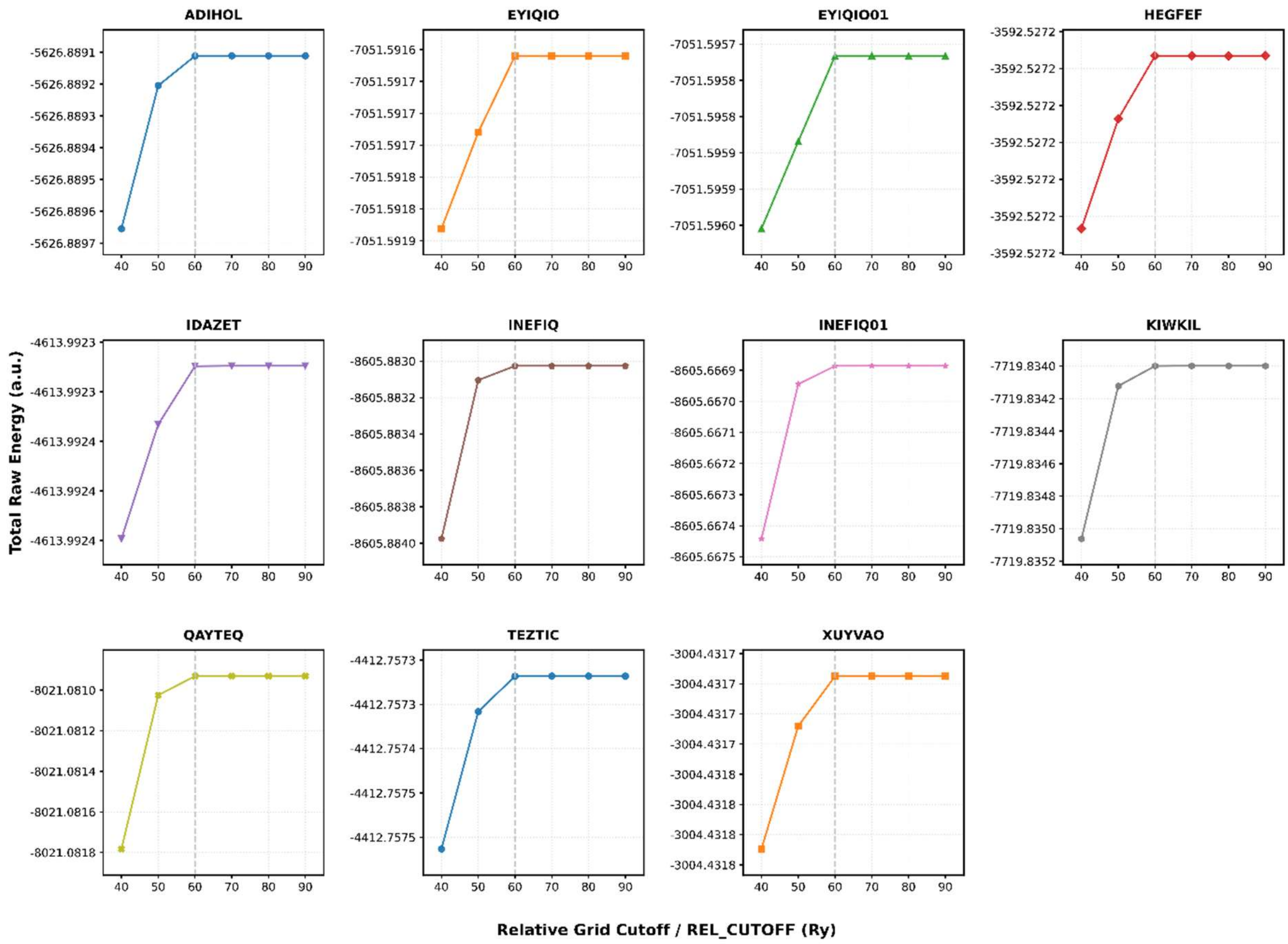

Figure S8. Relative density grid density convergence profiles tracking total raw energy with reference to the production baseline.

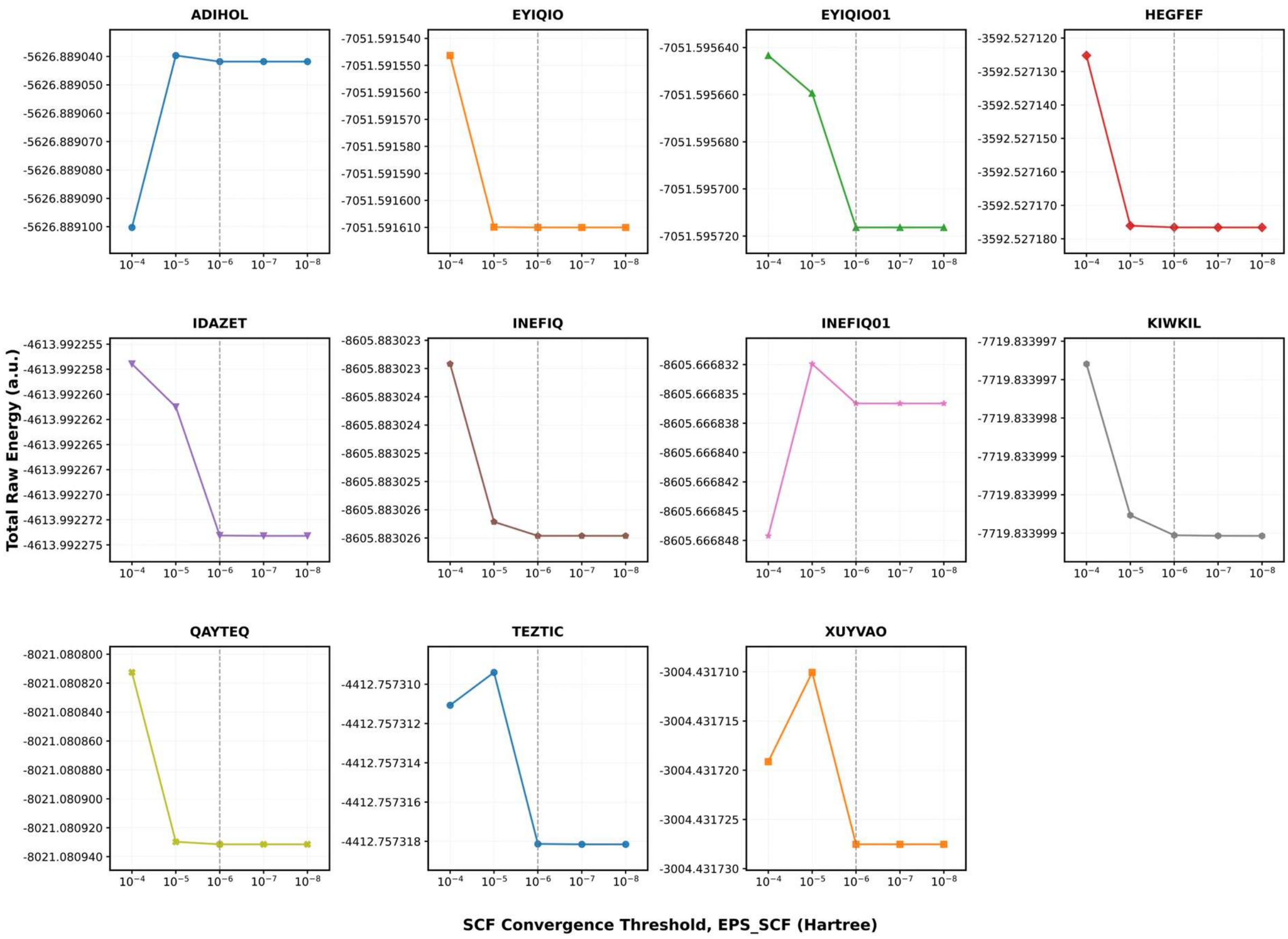

Figure S9. Electronic wavefunction optimisation convergence profiles showing the stability of total energy against tighter electronic thresholds.

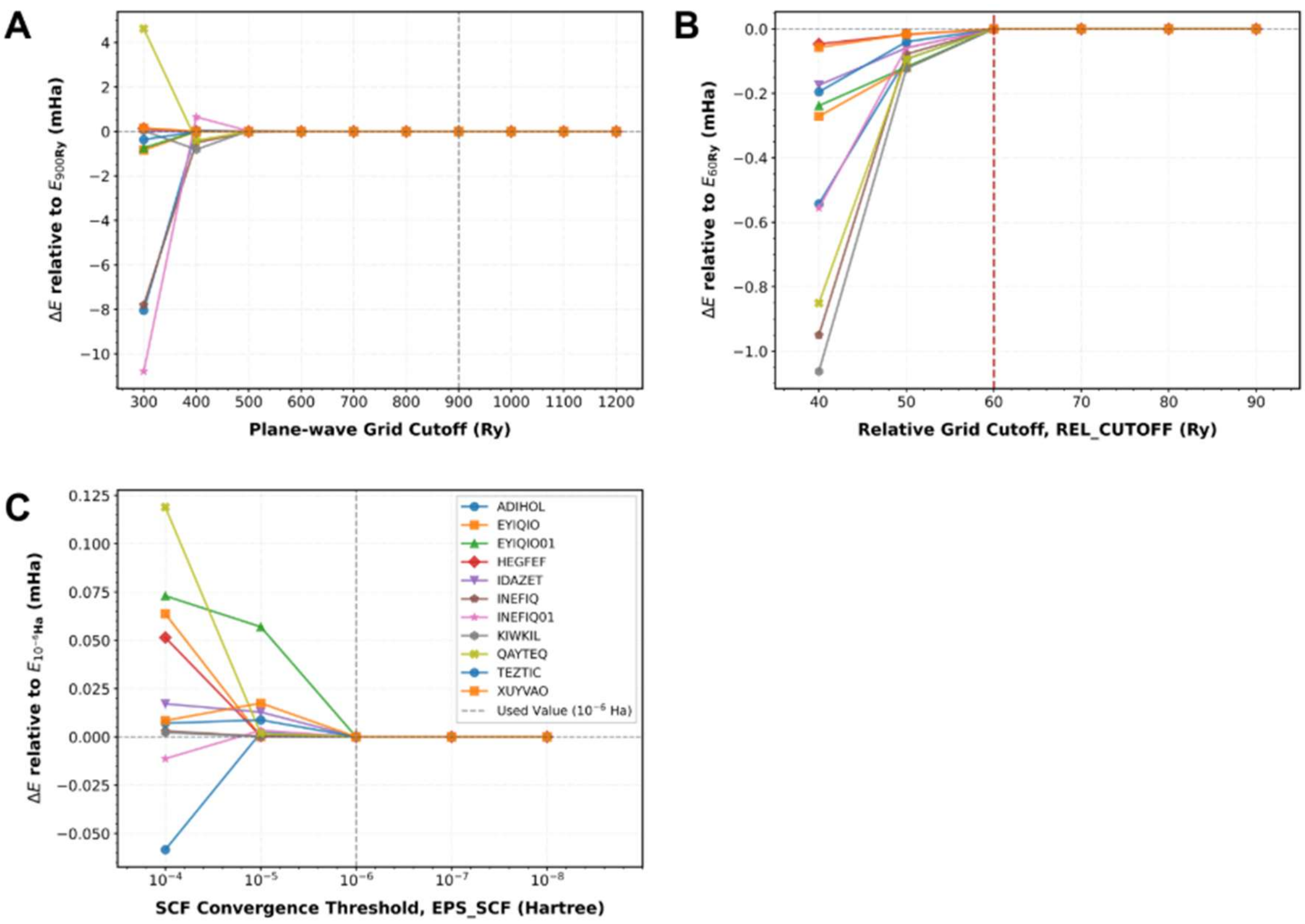

Figure S10. Relative energy convergence profiles across the studied crystal systems as a function of (A) plane-wave grid cutoff. (B) relative grid density. (C) electronic wavefunction optimisation threshold. Panels show the relative energy variations (ΔE) where dashed lines indicate the selected calculation parameters.

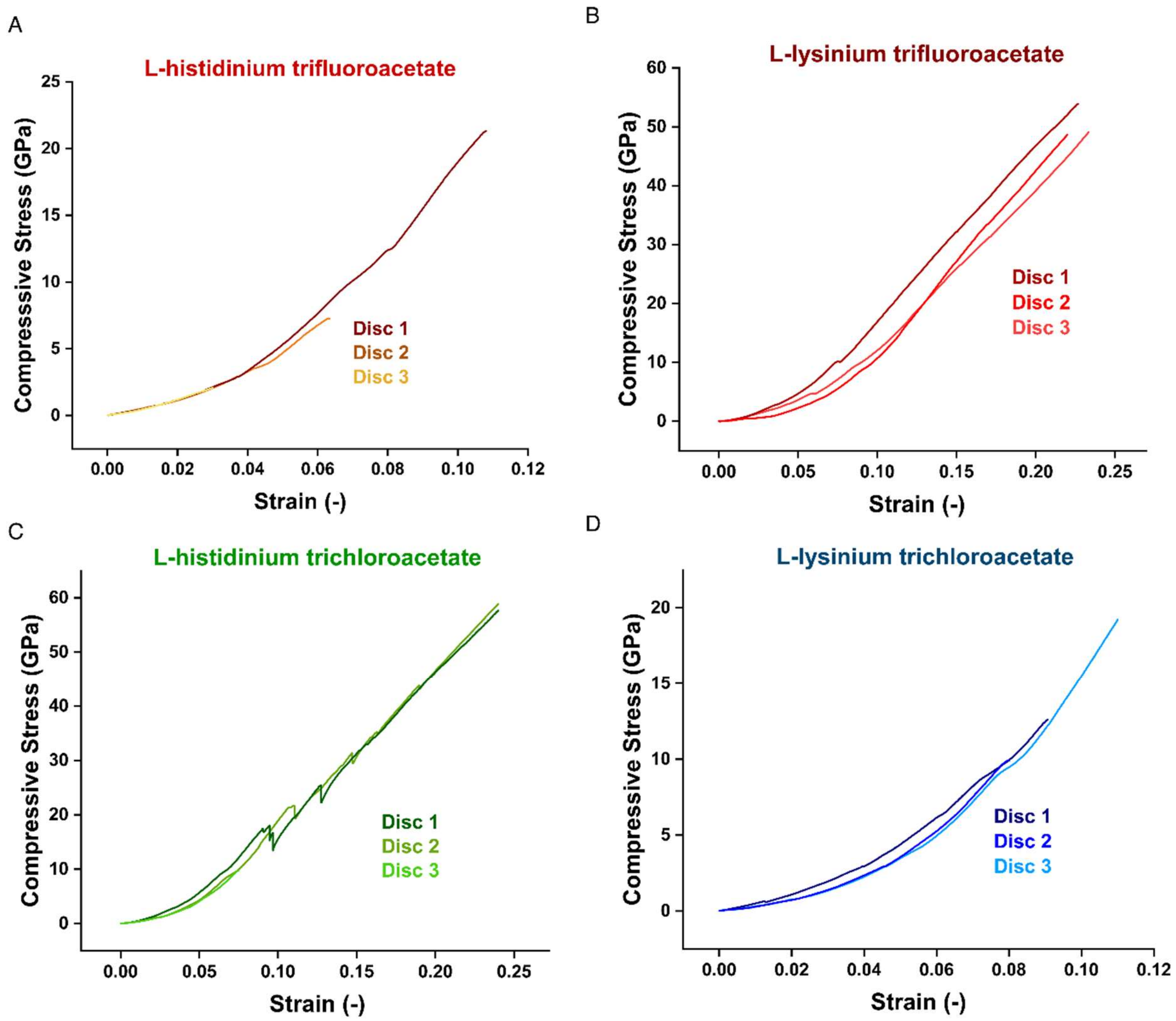

Figure S11. Full stress-strain curves for three discs with diameter = 3.3 cm of (A) L-histidinium trifluoroacetate. (B) L-lysinium trifluoroacetate. (C) L-histidinium trichloroacetate. (D) L-lysinium trichloroacetate.

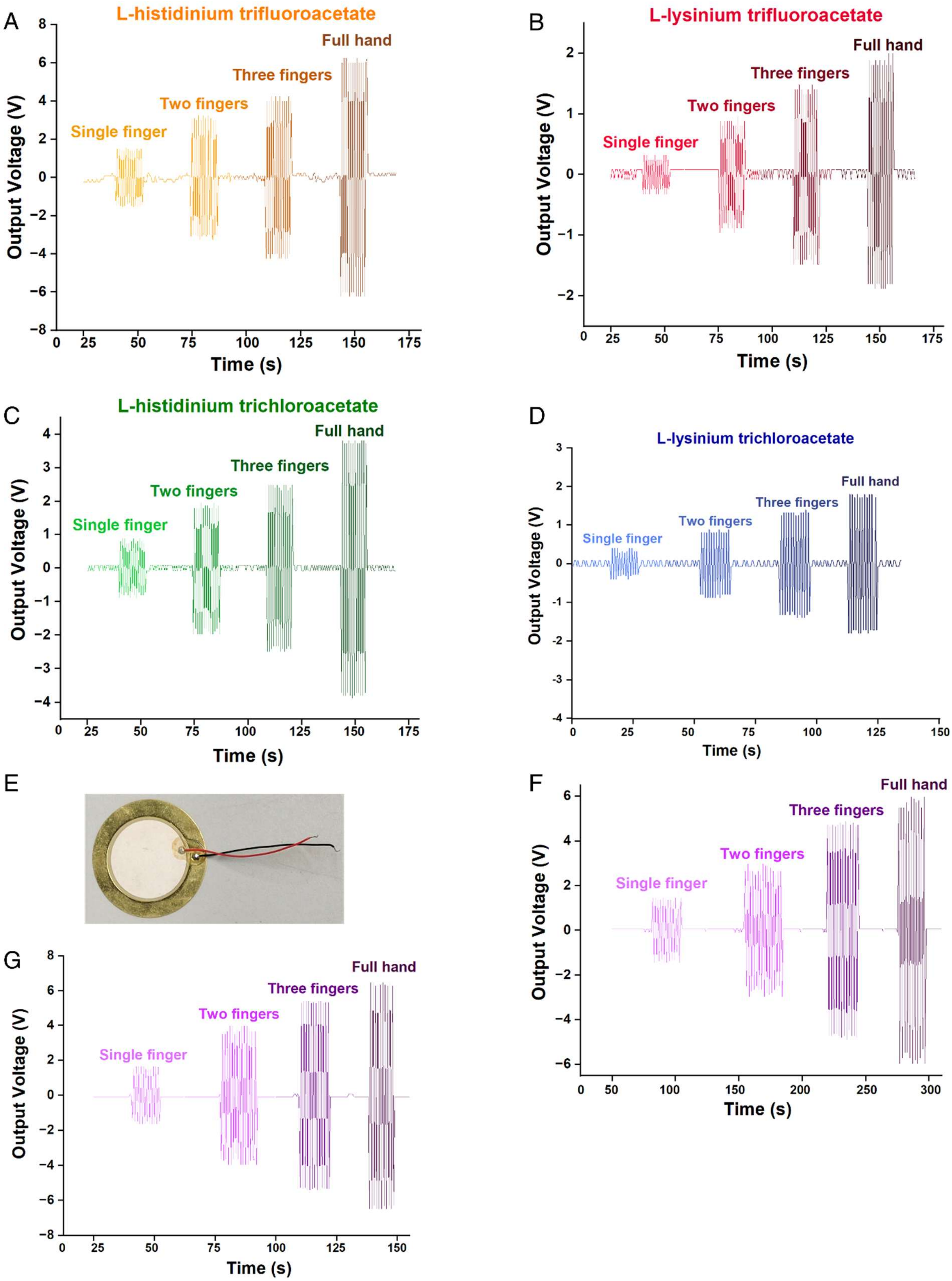

Figure S12. Energy harvesting studies of the amino acid trihaloacetates in reverse connection (A) L-histidinium trifluoroacetate. (B) L-lysinium trifluoroacetate. (C) L-histidinium trichloroacetate. (D) L-lysinium trichloroacetate (E) Photograph of the commercial piezo buzzer of diameter = 3.5 cm from RS Pro (Stock No. 837-7840). Open circuit voltage (OCV) of the commercial piezo buzzer in (F) Forward connection. (G) Reverse connection.

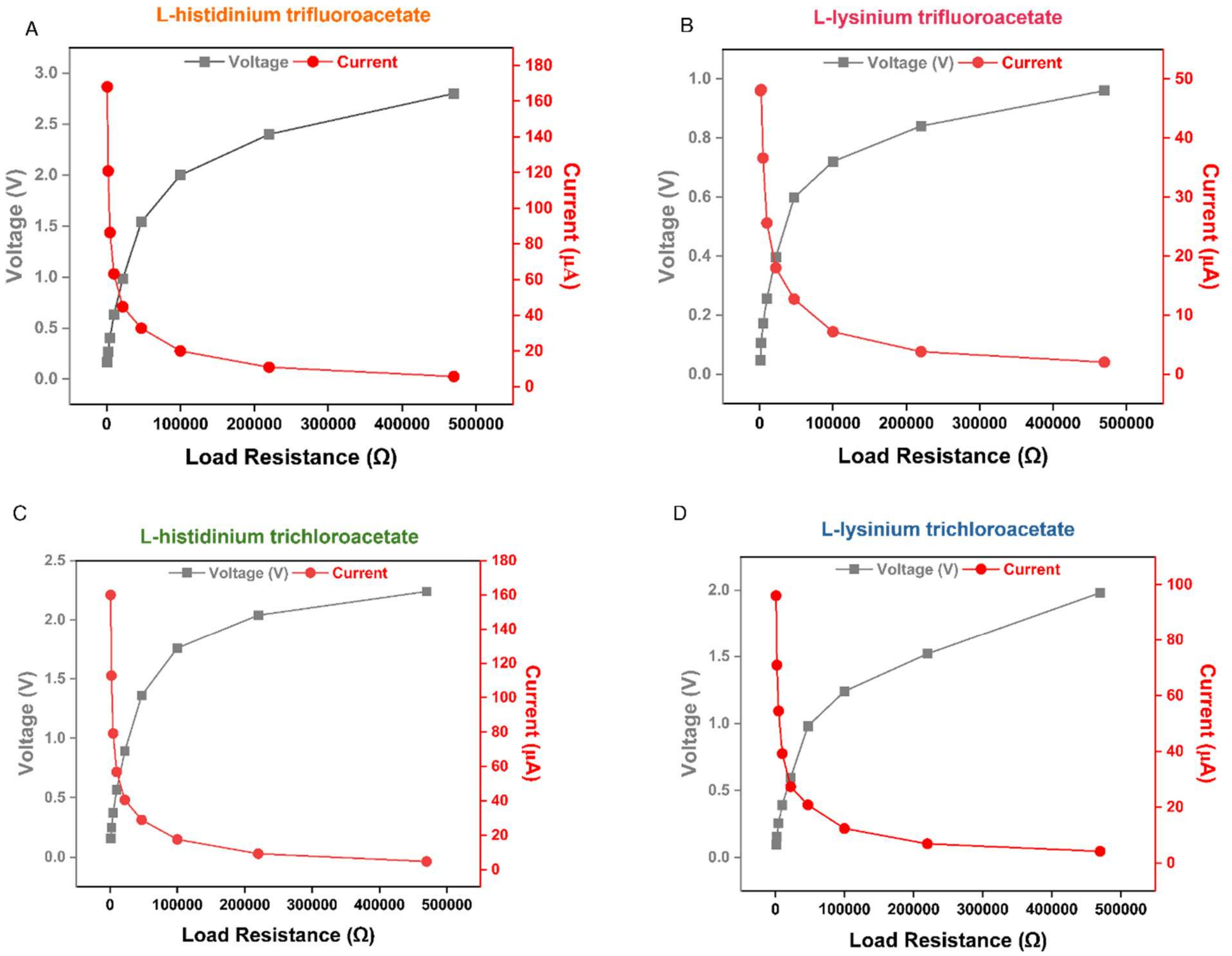

Figure S13. Voltage and current plots of trihaloacetates of amino acids (A) L-histidinium trifluoroacetate. (B) L-lysinium trifluoroacetate. (C) L-histidinium trichloroacetate. (D) L-histidinium trichloroacetate.

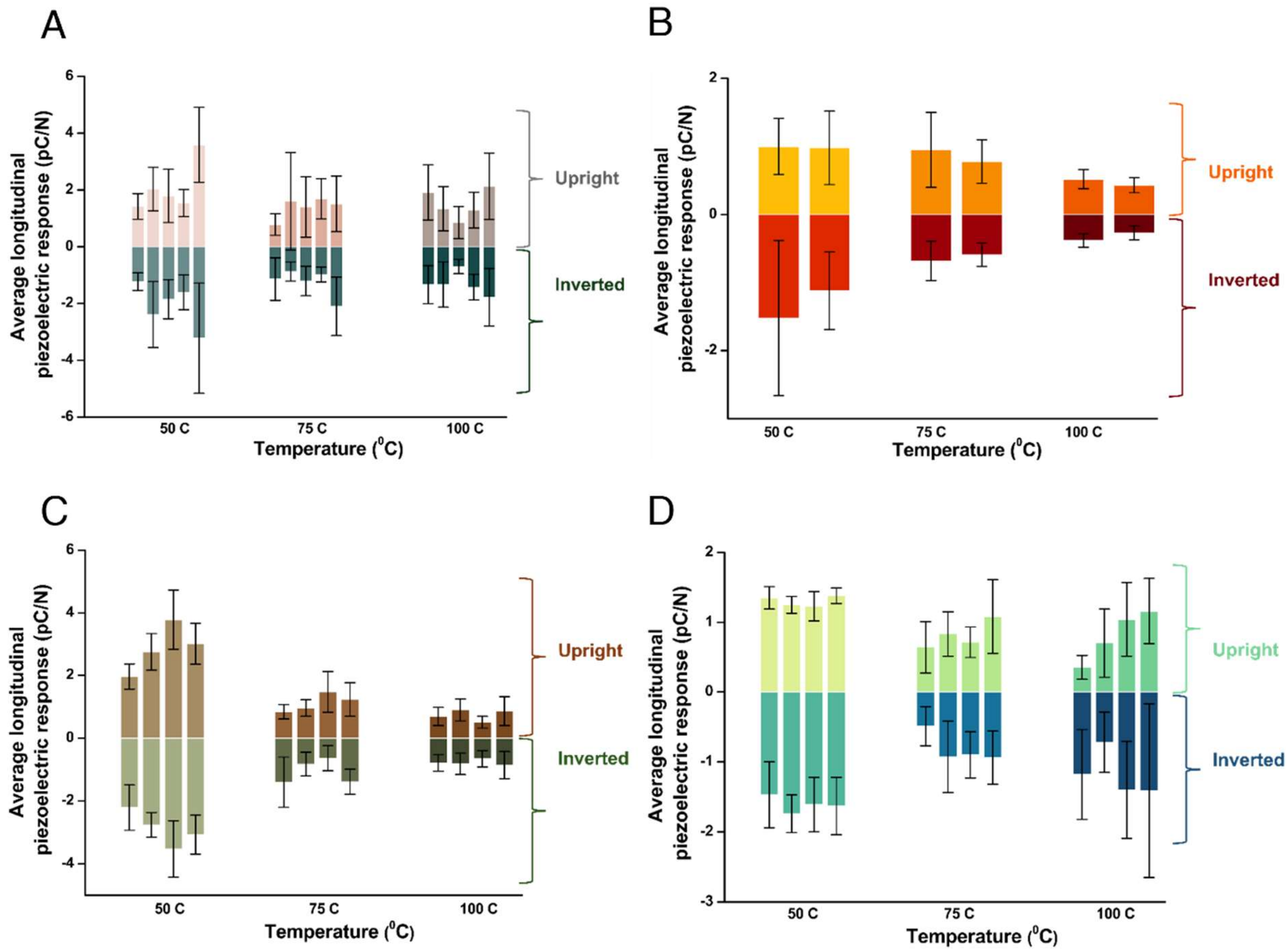

Figure S14. Temperature dependant d_{33}^{eff} studies of the multicomponent salts based on amino acids (A) L-histidinium trifluoroacetate. (B) L-lysinium trifluoroacetate. (C) L-histidinium trichloroacetate. (D) L-lysinium trichloroacetate.

Supplementary Tables

Table S1. Thickness measurements of L-argininium trifluoroacetate discs with a diameter = 3.3 cm using digital vernier callipers. Units were in mm.

Sample	Weight (g)	1	2	3	4	5	Average	Standard deviation
1	1.823	1.15	1.20	1.03	0.98	0.79	1.03	0.14

Table S2. Local piezoelectric measurements of L-argininium trifluoroacetate discs with a diameter = 3.3 cm. Units were pC/N.

Sample	Position	A	B	C	D	E	Avg.	Standard deviation
1	Upright	-3.50	-3.10	-2.23	-3.29	-2.12	-2.85	0.56
	Inverted	2.20	1.61	3.24	1.08	1.24	1.87	0.78

Table S3. Thickness measurements of L-argininium bis(trifluoroacetate) discs with a diameter = 3.3 cm using digital vernier callipers. Units were in mm.

Sample	Weight (g)	1	2	3	4	5	Avg.	Standard deviation
1	2.553	2.50	2.51	2.26	2.70	2.47	2.49	0.14

**2**	2.615	3.04	2.99	2.92	2.96	2.80	2.94	0.08
**3**	2.743	3.19	2.57	2.60	2.60	2.71	2.73	0.23

Table S4. Local piezoelectric measurements of L-argininium bis(trifluoroacetate) discs with a diameter = 3.3 cm. Units were pC/N.

Sample	Position	A	B	C	D	E	Avg.	Standard deviation
**1**	Upright	4.30	0.51	0.74	0.94	2.64	1.83	1.45
	Inverted	-3.50	-0.84	-0.56	-1.26	-1.26	-1.48	1.04
**2**	Upright	3.24	1.80	1.27	0.91	0.93	1.63	0.87
	Inverted	-2.51	-1.35	-1.10	-1.20	-0.99	-1.43	0.55
**3**	Upright	1.74	1.23	1.06	0.69	2.03	1.35	0.48
	Inverted	-2.76	-1.43	-1.57	-1.60	0.48	-1.38	1.04

Table S5. Structural information of the studied multicomponent systems, including crystal system, unit cell content, and space group information.

Crystal	Crystal Name	Crystal System	Z	Z'	Atoms/Unit Cell	Space Group Number	Space Group
**XUYVAO**	*L-prolinium trichloroacetate*	Trigonal	3	1	75	144	$P3_1$
**QAYTEQ**	*L-histidinium trifluoroacetate*	Triclinic	2	2	56	1	P_1
**KIWKIL**	*L-lysinium trifluoroacetate*	Monoclinic	4	2	128	4	$P2_1$
**IDAZET**	*L-histidinium trichloroacetate*	Monoclinic	4	2	112	4	$P2_1$
**INEFIQ01**	*L-argininium bis(trifluoroacetate)*	Monoclinic	2	1	84	4	$P2_1$
**ADIHOL**	*L-argininium trifluoroacetate*	Monoclinic	2	1	68	4	$P2_1$
**INEFIQ**	*L-argininium bis(trifluoroacetate)*	Monoclinic	2	1	84	4	$P2_1$
**TEZTIC**	*L-lysinium trichloroacetate*	Monoclinic	2	1	64	4	$P2_1$

Table S6. Thickness measurements of L-histidinium trifluoroacetate discs with a diameter = 3.3 cm using digital vernier callipers. Units were in mm.

Sample	Weight (g)	1	2	3	4	5	Avg.	Standard deviation
**1**	2.125	2.52	2.61	2.58	2.56	2.52	2.56	0.03
**2**	2.112	2.55	2.73	2.48	2.47	2.47	2.54	0.10
**3**	2.137	2.61	2.62	2.72	2.67	2.71	2.67	0.04
**4**	1.610	1.96	1.91	1.90	1.92	2.12	1.96	0.08
**5**	1.670	2.46	2.24	2.21	2.23	2.25	2.28	0.09

Table S7. Local piezoelectric measurements of L-histidinium trifluoroacetate discs with a diameter = 3.3 cm. Units were pC/N.

Sample	Position	A	B	C	D	E	Avg.	Standard deviation
**1**	Upright	5.00	3.35	2.23	3.59	2.61	3.36	0.96

Sample	Position	A	B	C	D	E	Avg.	Standard deviation
	Inverted	-4.72	-1.60	-2.03	-4.95	-2.93	-3.25	1.37
2	Upright	2.12	4.36	7.19	2.21	2.09	3.59	1.99
	Inverted	-2.97	-4.60	-7.35	-1.87	-1.48	-3.65	2.14
3	Upright	3.55	1.69	4.32	4.70	4.81	3.81	1.15
	Inverted	-3.95	-0.81	-2.66	-3.42	-5.19	-3.21	1.45
4	Upright	2.07	3.55	2.30	3.45	3.98	3.07	0.75
	Inverted	-1.30	-3.51	-1.62	-1.77	-1.46	-1.93	0.80
5	Upright	2.84	1.82	3.19	2.97	1.37	2.44	0.71
	Inverted	-2.69	-2.90	-1.49	-1.46	-1.79	-2.07	0.61

Table S8. Thickness measurements of L-lysinium trifluoroacetate discs with a diameter = 3.3 cm using digital vernier callipers. Units were in mm.

Sample	Weight (g)	1	2	3	4	5	Avg.	Standard Deviation
1	2.920	3.24	3.06	2.64	2.40	2.12	2.69	0.41
2	3.065	3.20	3.07	2.89	2.51	2.24	2.78	0.36
3	3.029	3.16	3.21	3.15	2.99	2.45	2.99	0.28
4	2.888	2.47	1.94	1.87	1.97	1.96	2.04	0.22
5	5.389	5.15	4.80	4.91	4.91	5.29	5.01	0.18
6	5.497	5.03	5.17	5.14	4.98	4.84	5.03	0.12

Table S9. Local piezoelectric measurements of L-lysinium trifluoroacetate discs with a diameter = 3.3 cm. Units were pC/N.

Sample	Position	A	B	C	D	E	Avg.	Standard deviation
1	Upright	0.51	0.25	0.22	0.21	0.46	0.33	0.13
	Inverted	-0.43	-0.30	-0.32	-0.30	-0.51	-0.37	0.08
2	Upright	0.38	0.32	0.30	0.45	0.42	0.37	0.06
	Inverted	-0.21	-0.25	-0.35	-0.32	-0.38	-0.30	0.06
3	Upright	0.89	0.25	0.29	0.39	0.41	0.45	0.23
	Inverted	-0.69	-0.45	-0.15	-0.20	-0.27	-0.35	0.20
4	Upright	0.33	0.18	0.25	0.27	0.48	0.30	0.10
	Inverted	-0.16	-0.35	-0.43	-0.33	-0.24	-0.30	0.09
5	Upright	0.39	0.48	0.30	1.99	1.11	0.85	0.64
	Inverted	-0.41	-0.40	-2.75	-0.44	-0.71	-0.94	0.91
6	Upright	0.99	1.38	1.52	1.48	1.14	1.30	0.20
	Inverted	-1.19	-1.04	-0.88	-1.25	-0.22	-0.92	0.37

Table S10. Thickness measurements of L-histidinium trichloroacetate discs with a diameter = 3.3 cm using digital vernier callipers. Units were in mm.

Sample	Weight (g)	1	2	3	4	5	Avg.	Standard deviation
1	3.095	2.54	2.42	2.68	2.55	2.37	2.51	0.11
2	3.121	2.73	2.96	2.96	2.49	2.42	2.71	0.23
3	2.842	3.07	3.22	3.26	3.29	3.32	3.23	0.09
4	2.829	3.30	3.12	3.06	3.26	3.15	3.18	0.09
5	2.759	2.85	2.59	2.31	2.95	2.74	2.69	0.22
6	2.435	2.03	2.28	2.69	2.53	2.97	2.50	0.32
7	3.731	3.41	3.41	3.44	3.45	3.57	3.46	0.06
8	3.484	3.28	3.45	3.37	3.36	3.33	3.36	0.06
9	3.572	3.18	2.76	2.81	2.66	2.69	2.82	0.19
10	3.789	3.37	3.20	3.09	3.06	2.90	3.12	0.16
11	2.952	2.98	2.74	2.59	3.03	2.99	2.87	0.17

Table S11. Local piezoelectric measurements of L-histidinium trichloroacetate discs with a diameter = 3.3 cm. Units were pC/N.

Sample	Position	A	B	C	D	E	Avg.	Standard deviation
1	Upright	2.80	4.63	3.28	1.46	4.09	3.25	1.10
	Inverted	-3.53	-3.69	-1.19	-1.90	-3.42	-2.75	1.01
2	Upright	2.31	2.80	1.80	2.05	1.92	2.18	0.35
	Inverted	-1.58	-1.03	-2.05	-1.23	-4.29	-2.04	1.18
3	Upright	1.22	2.92	2.01	3.81	5.17	3.03	1.38
	Inverted	-2.79	-1.09	-3.69	-2.25	-1.01	-2.17	1.02
4	Upright	3.62	3.05	2.75	2.71	3.02	3.03	0.33
	Inverted	-5.70	-1.53	-1.15	-2.23	-2.88	-2.70	1.61
5	Upright	1.34	1.25	1.78	1.18	1.29	1.37	0.21
	Inverted	-0.55	-1.65	-1.21	-1.16	-2.09	-1.33	0.52
6	Upright	1.07	1.48	0.87	0.87	1.24	1.11	0.23
	Inverted	-2.92	-1.88	-0.84	-1.23	-2.35	-1.84	0.75
7	Upright	0.80	0.94	2.02	3.41	0.84	1.60	1.01
	Inverted	-4.47	-3.70	-5.04	-4.11	-4.57	-4.38	0.45
8	Upright	1.32	0.88	2.85	3.03	2.45	2.11	0.85
	Inverted	-1.47	-0.88	-3.16	-1.32	-2.42	-1.85	0.83
9	Upright	1.63	1.69	0.90	1.05	2.40	1.53	0.53
	Inverted	-1.12	-0.98	-0.66	-0.94	-1.00	-0.94	0.15
10	Upright	1.38	1.90	2.54	2.63	2.49	2.19	0.48
	Inverted	-1.20	-1.23	-1.39	-0.97	-0.92	-1.14	0.17

Sample	Position	A	B	C	D	E	Avg.	Standard deviation
**11**	Upright	1.20	1.19	1.46	1.86	1.55	1.45	0.25
	Inverted	-0.90	-1.30	-1.37	-2.32	-1.75	-1.53	0.48

Table S12. Thickness measurements of L-lysinium trichloroacetate discs with a diameter = 3.3 cm using digital vernier callipers. Units were in mm.

Sample	Weight (g)	1	2	3	4	5	Avg.	Standard deviation
**1**	2.603	2.72	2.36	1.91	1.93	2.40	2.26	0.31
**2**	2.580	3.22	3.03	3.31	2.66	2.62	2.97	0.28
**3**	2.451	2.44	2.43	2.62	2.22	2.25	2.39	0.15
**4**	2.615	2.92	2.97	2.85	2.97	2.42	2.83	0.21
**5**	2.352	2.74	2.20	2.14	1.91	2.10	2.22	0.28
**6**	2.404	2.51	2.37	2.22	2.06	1.67	2.17	0.29
**7**	2.796	2.66	2.48	2.68	2.67	2.90	2.68	0.13
**8**	3.368	3.30	3.37	3.29	3.05	3.04	3.21	0.14
**9**	3.301	3.33	3.20	3.43	3.42	3.32	3.34	0.08
**10**	3.571	3.07	2.97	2.86	2.76	2.79	2.89	0.12
**11**	3.537	3.33	2.95	2.86	2.82	2.78	2.95	0.20
**12**	3.699	3.60	3.52	3.36	3.20	3.40	3.42	0.14
**13**	3.368	3.29	3.14	3.19	3.28	3.26	3.23	0.06

Table S13. Local piezoelectric measurements of L-lysinium trichloroacetate discs with a diameter = 3.3 cm. Units were pC/N.

Sample	Position	A	B	C	D	E	Avg.	Standard deviation
**1**	Upright	-1.42	-1.07	-1.93	-2.62	-1.14	-1.64	0.58
	Inverted	2.05	1.71	1.85	1.88	1.48	1.79	0.19
**2**	Upright	-4.25	-1.44	-1.71	-3.18	-2.23	-2.56	1.03
	Inverted	2.56	1.22	1.25	3.07	1.54	1.93	0.75
**3**	Upright	-1.14	-0.95	-1.00	-4.44	-4.57	-2.42	1.70
	Inverted	2.01	0.95	2.20	6.04	2.17	2.67	1.74
**4**	Upright	-1.98	-1.43	-0.75	-1.36	-1.01	-1.31	0.42
	Inverted	0.99	0.54	3.15	1.25	0.97	1.38	0.91
**5**	Upright	-2.12	-3.98	-4.18	-4.32	-2.26	-3.37	0.97
	Inverted	1.34	1.27	2.96	1.45	5.97	2.60	1.80
**6**	Upright	-3.79	-1.14	-2.41	-2.24	-2.76	-2.47	0.85
	Inverted	3.31	2.86	5.43	3.83	2.23	3.53	1.09
**7**	Upright	-2.13	-2.61	-3.31	-2.32	-3.54	-2.78	0.55
	Inverted	1.23	1.68	4.57	2.49	2.55	2.50	1.15
**8**	Upright	-0.67	-2.52	-1.52	-1.60	-1.46	-1.55	0.59

	Inverted	0.96	2.34	3.03	1.41	1.22	1.79	0.77
**9**	Upright	-1.03	-3.38	-0.80	-0.93	-0.91	-1.41	0.99
	Inverted	0.93	3.13	0.86	1.41	1.25	1.52	0.83
**10**	Upright	-0.81	-1.06	-2.26	-1.16	-1.40	-1.34	0.50
	Inverted	1.82	0.90	1.13	2.05	1.96	1.57	0.47
**11**	Upright	-4.14	-0.65	-1.21	-1.08	-1.21	-1.66	1.26
	Inverted	3.92	0.99	1.06	0.90	0.61	1.50	1.22
**12**	Upright	-0.77	-1.39	-4.40	-3.81	-0.55	-2.18	1.60
	Inverted	1.48	4.12	4.34	2.05	0.50	2.50	1.50
**13**	Upright	-0.56	-2.61	-1.22	-0.41	-0.76	-1.11	0.80
	Inverted	2.56	2.01	1.11	0.37	1.46	1.50	0.75

Table S14. Quantitative analysis (degree of coherency) of local crystallite orientation performed on SEM micrographs of amino acid trihaloacetates using the OrientationJ Measure plugin available in ImageJ.

No:	L-histidinium trifluoroacetate	L-lysinium trifluoroacetate	L-histidinium trichloroacetate	L-lysinium trichloroacetate
1	0.73	0.65	0.67	0.54
2	0.71	0.52	0.70	0.58
3	0.52	0.61	0.84	0.75
4	0.78	0.52	0.72	0.73
5	0.74	0.66	0.75	0.71
6	0.57	0.51	0.80	0.44
7	0.69	0.51	0.77	0.78
8	0.65	0.52	0.67	0.57
9	0.52	0.53	0.60	0.66
10	0.42	0.56	0.77	0.65
11	0.57	0.54	0.69	0.29
12	0.44	0.55	0.76	0.74
13	0.48	0.55	0.62	0.66
14	0.69	0.66	0.63	0.33
15	0.78	0.64	0.65	0.75
**Average degree of coherency**	**0.62 ± 0.12**	**0.57 ± 0.05**	**0.71 ± 0.07**	**0.61 ± 0.15**

Table S15. Intermolecular contact percentages contributions derived from 2D Hirshfeld fingerprint plots alongside predicted longitudinal piezoelectric coefficients (d_{33}^{eff}) for the modelled crystal series.

Crystal System (CSD ID)	Component/Ion Pair	Halogen---H (%)	Halogen---Halogen (%)	Halogen---O (%)	d_{33}^{eff}(pC/N)
L-lysinium trifluoroacetate (KIWKIL, Z'=2)	Pair 1	31.8	0.6	4	1.2
	Pair 2 (Z'=2)	31.4	0.6	3.1	
L-lysinium trichloroacetate (TEZTIC)	Monoclinic Pair	27.5	2.6	6.9	2.1
L-histidinium trifluoroacetate (QAYTEQ, Z'=2)	Pair 1	29.6	6	3.7	4
	Pair 2	14	5.8	5.7	
L-histidinium trichloroacetate (IDAZET, Z'=2)	Pair 1	20.4	6.5	3.4	7.7

Table S16. Computed piezoelectric coefficients (d_{ij}) and predicted hydrogen-bond counts for the multicomponent systems.

Crystal	Crystal Name	Experimental d_{33} (pC/N)	d_{11} (pC/N)	d_{22} (pC/N)	d_{33} (pC/N)	d_{15} (pC/N)	Max d_{ij} (pC/N)	Number of H-bonds
**XUYVAO**	*L-prolinium trichloroacetate*	7.5	1	0.6	7.5	5	7.5 (d_{33})	4
**QAYTEQ**	*L-histidinium trifluoroacetate*	4	4	3.4	3.9	8.5	8.5 (d_{15})	3
**KIWKIL**	*L-lysinium trifluoroacetate*	1.2	0	1.2	0	0	1.2 (d_{22})	22
**IDAZET**	*L-histidinium trichloroacetate*	7.7	0	7.7	0	0	23 (d_{23})	12
**INEFIQ01**	*L-argininium bis(trifluoroacetate)*	6.4	0	6.4	0	0	6.4 (d_{22})	18
**INEFIQ**	*L-argininium bis(trifluoroacetate)*	5.9	0	5.9	0	0	5.9 (d_{22})	14
**ADIHOL**	*L-argininium trifluoroacetate*	4.3	0	4.3	0	0	4.3 (d_{33})	13
**TEZTIC**	*L-lysinium trichloroacetate*	2.1	0	2.1	0	0	5.9 (d_{16})	5

Table S17. Calculated piezoelectric strain tensor components (d_{ij}, pC/N) for studied amino acid-based multicomponent salts, alongside their corresponding space groups, the full elastic tensor components (C_{ij}, GPa), Piezoelectric Charge Constants (C/m^2), Dielectric Constants, and Piezoelectric Voltage Constants (g_{ij}, mV·m/N). This table provides a complete dataset linking directional mechanical properties to electromechanical performance, enabling detailed analysis of structure-property relationships and anisotropic piezoelectric response in these systems.

Crystal: *L-histidinium trifluoroacetate*, **CSD ID:** QAYTEQ, **Space Group:** P_1

```
Piezoelectric Charge Constants [e] (C/m²)
         1       2       3       4       5       6
1    0.115  -0.005   0.054  -0.056  -0.113   0.029
2    0.009   0.187   0.147  -0.064  -0.035  -0.014
3   -0.058  -0.090  -0.183   0.054   0.008 -0.0349

Elastic Constants (GPa)
           1          2          3          4          5          6
 1     28.342     15.973     18.646     -1.128     -1.999      4.862
 2     15.973     75.082     23.278      0.027     -2.039     15.828
 3     18.646     23.278     46.846     -6.246     -0.818      5.305
 4     -1.128      0.027     -6.246     17.174      2.261     -1.252
 5     -1.999     -2.039     -0.818      2.261     12.727      0.704
 6      4.862     15.828      5.305     -1.252      0.704     12.389

Piezoelectric Strain Constants [d] (pC/N)
           1             2             3             4             5             6
1          4.047        -1.807        -0.325        -1.765        -8.504         3.522
2         -2.578         3.411         2.701        -3.194        -1.555        -5.918
3          0.774         0.179        -3.916         1.629         0.338        -1.532

 GPa   |   Bulk modulus   |   Young modulus  |   Shear modulus  |   Poisson ratio  |
 Voigt |        29.563    |        37.645    |        14.617    |          0.288   |
 Reuss |        20.653    |        28.694    |        11.311    |          0.268   |
 Hill  |        25.108    |        33.180    |        12.964    |          0.280   |

Dielectric Constants
           1             2             3
1          2.346         0.427         0.327
2          0.427         3.532         0.536
3          0.327         0.536         3.717

Piezoelectric Voltage Constants [g] (mV m/N)
           1             2             3             4             5             6
1        163.711       -91.276       -18.412       -46.568      -299.869       176.535
2       -120.175       133.012        98.028       -99.731         7.076      -233.328
3         14.190        11.702      -137.030        63.720        40.497       -64.503
```

Crystal: *L-lysinium trifluoroacetate*, **CSD ID:** KIWKIL, **Space Group:** $P2_1$

Piezoelectric Charge Constants [e] (C/m^2)

	1	2	3	4	5	6
1	0.000	0.000	0.000	0.002	0.000	0.000
2	-0.009	-0.042	-0.001	0.000	0.017	0.000
3	0.000	0.000	0.000	-0.001	0.000	0.007

Elastic Constants (GPa)

	1	2	3	4	5	6
1	**46.209**	13.237	18.606	0.000	-2.902	0.000
2	13.237	**40.784**	14.941	0.000	0.724	0.000
3	18.606	14.941	**36.373**	0.000	-2.410	0.000
4	0.000	0.000	0.000	**16.775**	0.000	0.999
5	-2.902	0.724	-2.410	0.000	**15.741**	0.000
6	0.000	0.000	0.000	0.999	0.000	**13.326**

Piezoelectric Strain Constants [d] (pC/N)

	1	2	3	4	5	6
1	0.000	0.000	0.000	0.161	0.000	0.041
2	0.010	**-1.264**	0.544	0.000	1.233	0.000
3	0.000	0.000	0.000	-0.111	0.000	0.549

GPa	Bulk modulus	Young modulus	Shear modulus	Poisson ratio
Voigt	24.104	35.763	14.274	0.253
Reuss	23.777	34.524	13.722	0.258
Hill	23.941	35.144	13.998	0.255

Dielectric Constants

	1	2	3
1	2.388	0.000	0.025
2	0.000	2.452	0.000
3	0.025	0.000	2.369

Piezoelectric Voltage Constants [g] (mV m/N)

	1	2	3	4	5	6
1	-0.000	0.027	-0.011	7.647	-0.026	1.768
2	0.464	-58.003	24.988	-0.003	56.605	-0.003
3	-0.000	0.004	-0.002	-5.348	-0.004	26.187

Crystal: *L-argininium trifluoroacetate*, **CSD ID:** ADIHOL, **Space Group:** $P2_1$

Piezoelectric Charge Constants [e] (C/m^2)

	1	2	3	4	5	6
1	0.000	0.000	0.000	-0.026	0.000	-0.002
2	0.064	-0.172	-0.069	0.000	-0.019	0.000
3	0.000	0.000	0.000	-0.023	0.000	-0.029

Elastic Constants (GPa)

	1	2	3	4	5	6
1	**42.405**	17.880	12.369	0.000	-5.348	0.000
2	17.880	**50.749**	18.874	0.000	-0.495	0.000
3	12.369	18.874	**44.566**	0.000	9.483	0.000
4	0.000	0.000	0.000	**13.285**	0.000	-4.013
5	-5.348	-0.495	9.483	0.000	**11.491**	0.000
6	0.000	0.000	0.000	-4.013	0.000	**22.926**

Piezoelectric Strain Constants [d] (pC/N)

	1	2	3	4	5	6
1	0.000	0.000	0.000	-2.151	0.000	-0.474
2	3.650	**-4.386**	-0.802	0.000	0.447	0.000
3	0.000	0.000	0.000	-2.265	0.000	-1.670

GPa	Bulk modulus	Young modulus	Shear modulus	Poisson ratio
Voigt	26.219	38.734	15.447	0.254
Reuss	25.540	30.745	11.831	0.299
Hill	25.880	34.803	13.639	0.276

Dielectric Constants

	1	2	3
1	2.141	0.000	0.346
2	0.000	2.397	0.000
3	0.346	0.000	2.665

Piezoelectric Voltage Constants [g] (mV m/N)

	1	2	3	4	5	6
1	-0.014	0.017	0.003	-77.565	-0.002	-7.596
2	163.947	-197.018	-36.028	0.009	20.056	0.002
3	-0.001	0.001	0.000	-101.029	-0.000	-83.505

Crystal: *L-argininium bis(trifluoroacetate)*, **CSD ID:** INEFIQ, **Space Group:** $P2_1$

Piezoelectric Charge Constants [e] (C/m²)

	1	2	3	4	5	6
1	0.000	0.000	0.000	-0.034	0.000	-0.015
2	0.017	-0.166	-0.032	0.000	-0.019	0.000
3	0.000	0.000	0.000	-0.025	0.000	-0.005

Elastic Constants (GPa)

	1	2	3	4	5	6
1	**47.491**	16.488	24.082	0.000	-1.500	0.000
2	16.488	**38.161**	21.043	0.000	4.529	0.000
3	24.082	21.043	**40.818**	0.000	-6.329	0.000
4	0.000	0.000	0.000	**24.560**	0.000	3.545
5	-1.500	4.529	-6.329	0.000	**22.312**	0.000
6	0.000	0.000	0.000	3.545	0.000	**13.609**

Piezoelectric Strain Constants [d] (pC/N)

	1	2	3	4	5	6
1	0.000	0.000	0.000	-1.274	0.000	-0.823
2	1.763	**-5.995**	1.401	0.000	0.872	0.000
3	0.000	0.000	0.000	-1.020	0.000	-0.143

GPa	Bulk modulus	Young modulus	Shear modulus	Poisson ratio
Voigt	27.744	41.144	16.420	0.253
Reuss	27.333	33.552	12.950	0.295
Hill	27.539	**37.407**	14.685	0.274

Dielectric Constants

	1	2	3
1	2.274	0.000	-0.075
2	0.000	2.278	0.000
3	-0.075	0.000	2.330

Piezoelectric Voltage Constants [g] (mV m/N)

	1	2	3	4	5	6
1	0.005	-0.016	0.004	-66.911	0.002	-41.405
2	83.387	-283.651	66.294	-0.009	41.240	-0.003
3	0.009	-0.031	0.007	-54.090	0.005	-9.880

Crystal: *L-histidinium trichloroacetate*, **CSD ID:** IDAZET, **Space Group:** $P2_1$

Piezoelectric Charge Constants [e] (C/m²)

	1	2	3	4	5	6
1	0.000	0.000	0.000	0.006	0.000	-0.000
2	-0.039	-0.038	0.270	0.000	-0.033	0.000
3	0.000	0.000	0.000	0.004	0.000	0.038

Elastic Constants (GPa)

	1	2	3	4	5	6
1	**40.827**	11.858	14.229	0.000	-5.233	0.000
2	11.858	**42.893**	16.165	0.000	-2.124	0.000
3	14.229	16.165	**21.233**	0.000	-3.015	0.000
4	0.000	0.000	0.000	**8.324**	0.000	-0.903
5	-5.233	-2.124	-3.015	0.000	**11.712**	0.000
6	0.000	0.000	0.000	-0.903	0.000	**9.096**

Piezoelectric Strain Constants [d] (pC/N)

	1	2	3	4	5	6
1	0.000	0.000	0.000	0.749	0.000	-0.025
2	-6.936	**-7.749**	23.066	0.000	-1.420	0.000
3	0.000	0.000	0.000	1.036	0.000	4.323

GPa	Bulk modulus	Young modulus	Shear modulus	Poisson ratio
Voigt	21.051	25.915	10.007	0.295
Reuss	18.172	22.384	8.644	0.295
Hill	19.612	24.149	9.326	0.295

Dielectric Constants

	1	2	3
1	2.506	0.000	-0.084
2	0.000	2.823	0.000
3	-0.084	0.000	2.484

Piezoelectric Voltage Constants [g] (mV m/N)

	1	2	3	4	5	6
1	0.000	0.000	-0.000	33.010	0.000	-0.857
2	-217.752	-243.297	724.193	0.000	-44.593	0.002
3	-0.003	-0.003	0.010	47.985	-0.001	199.996

Crystal: *L-lysinium trichloroacetate*, **CSD ID:** TEZTIC, **Space Group:** $P2_1$

Piezoelectric Charge Constants [e] (C/m²)

	1	2	3	4	5	6
1	0.000	0.000	0.000	-0.011	0.000	-0.084
2	-0.016	-0.059	-0.035	0.000	0.023	0.000
3	0.000	0.000	0.000	-0.016	0.000	0.020

Elastic Constants (GPa)

	1	2	3	4	5	6
1	**35.463**	14.441	16.074	0.000	-6.019	0.000
2	14.441	**32.083**	10.096	0.000	1.329	0.000
3	16.074	10.096	**42.127**	0.000	-5.063	0.000
4	0.000	0.000	0.000	**14.251**	0.000	0.810
5	-6.019	1.329	-5.063	0.000	**17.586**	0.000
6	0.000	0.000	0.000	0.810	0.000	**14.161**

Piezoelectric Strain Constants [d] (pC/N)

	1	2	3	4	5	6
1	0.000	0.000	0.000	-0.453	0.000	-5.908
2	0.934	**-2.196**	-0.467	0.000	1.695	0.000
3	0.000	0.000	0.000	-1.255	0.000	1.552

GPa	Bulk modulus	Young modulus	Shear modulus	Poisson ratio
Voigt	21.211	34.030	13.804	0.233
Reuss	20.452	31.502	12.669	0.243
Hill	20.831	32.769	13.237	0.238

Dielectric Constants

	1	2	3
1	2.599	-0.000	0.008
2	-0.000	2.599	0.000
3	0.008	0.000	2.652

Piezoelectric Voltage Constants [g] (mV m/N)

	1	2	3	4	5	6
1	0.005	-0.011	-0.002	-19.568	0.009	-245.041
2	40.283	-94.705	-20.139	-0.008	73.120	-0.023
3	0.004	-0.010	-0.002	-54.753	0.008	64.159

Crystal: *L-prolinium trichloroacetate*, **CSD ID:** XUYVAO, **Space Group:** $P3_1$

Piezoelectric Charge Constants [e] (C/m²)

	1	2	3	4	5	6
1	-0.0121	0.014	0.002	0.001	-0.030	0.007
2	0.0149	-0.034	-0.000	-0.038	0.016	0.021
3	-0.0142	-0.008	-0.175	-0.000	-0.000	0.004

Elastic Constants (GPa)

	1	2	3	4	5	6
1	**16.600**	7.480	5.974	-0.926	0.012	-4.745
2	7.480	**37.028**	9.236	1.621	0.445	-5.880
3	5.974	9.236	**25.977**	0.085	0.044	-1.592
4	-0.926	1.621	0.085	**8.741**	-1.155	0.081
5	0.012	0.445	0.044	-1.155	**6.240**	-1.054
6	-4.745	-5.880	-1.592	0.081	-1.054	**9.175**

Piezoelectric Strain Constants [d] (pC/N)

	1	2	3	4	5	6
1	-1.076	0.680	0.128	-0.695	-5.013	0.153
2	1.925	-0.664	-0.001	-3.762	2.584	3.270
3	1.449	1.510	**-7.571**	-0.061	0.008	0.924

GPa	Bulk modulus	Young modulus	Shear modulus	Poisson ratio
Voigt	13.888	21.438	8.626	0.243
Reuss	10.347	17.896	7.385	0.212
Hill	12.117	**19.681**	8.005	0.229

Dielectric Constants

	1	2	3
1	1.801	-0.329	0.006
2	-0.329	3.378	-0.003
3	0.006	-0.003	3.170

Piezoelectric Voltage Constants [g] (mV m/N)

	1	2	3	4	5	6
1	-60.138	34.462	6.087	-7.620	-240.066	-13.574
2	98.272	-35.625	-1.508	-174.432	153.956	154.498
3	49.554	51.323	-257.781	-2.386	0.750	31.743

Crystal: *Glycinium trichloroacetate*, **CSD ID:** HEGFEF, **Space Group:** $P4_1$

Piezoelectric Charge Constants [e] (C/m^2)

	1	2	3	4	5	6
1	0.000	0.000	0.000	-0.007	-0.009	0.000
2	0.000	0.000	0.000	-0.009	0.007	0.000
3	-0.011	-0.011	-0.070	0.000	0.000	0.000

Elastic Constants (GPa)

	1	2	3	4	5	6
1	**15.763**	7.832	7.200	0.000	0.000	-0.456
2	7.832	**15.763**	7.200	0.000	0.000	0.456
3	7.200	7.200	**47.880**	0.000	0.000	0.000
4	0.000	0.000	0.000	**4.956**	0.000	0.000
5	0.000	0.000	0.000	0.000	**4.956**	0.000
6	-0.456	0.456	0.000	0.000	0.000	**3.966**

Piezoelectric Strain Constants [d] (pC/N)

	1	2	3	4	5	6
1	0.000	0.000	0.000	-1.574	-1.959	0.000
2	0.000	0.000	0.000	-1.959	1.574	0.000
3	-0.060	-0.060	-1.460	0.000	0.000	0.000

GPa	Bulk modulus	Young modulus	Shear modulus	Poisson ratio
Voigt	13.764	17.043	6.587	0.293
Reuss	11.331	13.132	5.024	0.306
Hill	12.547	15.090	5.805	0.299

Dielectric Constants

	1	2	3
1	3.380	0.000	0.000
2	0.000	3.380	0.000
3	0.000	0.000	3.383

Piezoelectric Voltage Constants [g] (mV m/N)

	1	2	3	4	5	6
1	-0.008	-0.008	-0.191	-53.578	-65.870	0.000
2	0.009	0.009	0.219	-64.902	51.526	0.000
3	-1.988	-1.988	-48.578	0.088	-0.492	0.000

Crystal: *Guanidinium trichloroacetate*, **CSD ID:** EYIQIO, **Space Group:** $P4_1$

Piezoelectric Charge Constants [e] (C/m^2)

	1	2	3	4	5	6
1	0.000	0.000	0.000	-0.013	-0.006	0.000
2	0.000	0.000	0.000	-0.006	0.013	0.000
3	0.008	0.008	-0.009	0.000	0.000	0.000

Elastic Constants (GPa)

	1	2	3	4	5	6
1	**19.501**	15.148	9.950	0.000	0.000	0.295
2	15.148	**19.501**	9.950	0.000	0.000	-0.295
3	9.950	9.950	**27.820**	0.000	0.000	0.000
4	0.000	0.000	0.000	**11.724**	0.000	0.000
5	0.000	0.000	0.000	0.000	**11.724**	0.000
6	0.295	-0.295	0.000	0.000	0.000	2.176

Piezoelectric Strain Constants [d] (pC/N)

	1	2	3	4	5	6
1	0.000	0.000	0.000	-1.170	-0.572	0.000
2	0.000	0.000	0.000	-0.572	1.170	0.000
3	0.428	0.428	-0.664	0.000	0.000	0.000

GPa	Bulk modulus	Young modulus	Shear modulus	Poisson ratio
Voigt	15.213	18.754	7.243	0.294
Reuss	15.171	11.230	4.079	0.376
Hill	15.192	15.107	5.661	0.334

Dielectric Constants

	1	2	3
1	2.249	0.000	0.000
2	0.000	2.249	0.000
3	0.000	0.000	2.480

Piezoelectric Voltage Constants [g] (mV m/N)

	1	2	3	4	5	6
1	0.002	0.002	-0.004	-58.685	-28.688	0.000
2	-0.003	-0.003	0.005	-28.694	58.692	0.000
3	19.469	19.469	-30.205	-0.002	-0.012	0.000

Table S18. Dipole moments of relaxed crystal structures calculated using CP2K. Each component (X, Y, Z) and the total dipole are obtained from the Berry phase formalism, which captures the collective electronic polarisation of the unit cell. The values reflect the macroscopic dipole of the crystal and generally differ from a simple sum of individual molecular dipoles due to periodicity, electronic delocalisation, and molecular orientation within the lattice.

Crystal	X	Y	Z	Crystal Dipole (Debye)
**ADIHOL**	-1.03E-10	-11.025384	-2.04E-10	11.03
**HEGFEF**	0.000139258	0.000145005	17.601467	17.6
**IDAZET**	-1.23E-06	-15.773294	2.10E-06	15.77
**INEFIQ**	1.77E-11	-32.869033	9.21E-12	32.87
**INEFIQ01**	-1.02E-10	-36.706538	1.34E-10	36.71
**KIWKIL**	-7.87E-07	10.380964	-2.46E-07	10.38
**QAYTEQ**	-4.2403511	5.2309604	43.43172	43.95
**TEZTIC**	-1.12E-06	-5.0957618	-4.67E-07	5.1
**XUYVAO**	0.413482	-0.12698458	12.733581	12.74

Table S19. Comparison of the Young's modulus (GPa) of various organic piezoelectric and commercial materials.

Material	Class	Young's modulus (GPa)	Method	Reference
trans-4-hydroxy-L-proline	Amino acid (Organic)	12 - 24	Uniaxial compression testing	22
γ-glycine sulfamic acid cocrystal	Amino acid (Organic)	17	Computational	23
Diphenylalanine nanotubes	Peptide (Organic)	19 - 27	AFM nanoindentation	24-26
S-Mand•L-Lys•5H_2O (multicomponent salt)	Amino acid (Organic)	30 (uncoated) 51 (polymer coated)	Uniaxial compression testing	13
BPA/Ac-DL-ala	Amino acid (Organic)	38.8	Nanoindentation	27
γ-glycine	Amino acid (Organic)	28 - 56	Uniaxial compression testing	22, 28
α-glycine	Amino acid (Organic)	44	Nanoindentation	28
Pro-Phe-Phe assemblies	Peptide (Organic)	44	Nanoindentation	29
Dip-Dip	Peptide (Organic)	70	AFM nanoindentation	30
PZT-5H	Ceramics (Commercial)	41.8 – 48.1 (unpoled) 43.4 – 58.2 (poled)	Tensile testing	31

Table S20. Comparison of the piezoelectric coefficients (pC/N or pm/V) and output performance (V) of various organic piezoelectric materials. Output performance is given as either V_{oc} is the output circuit voltage or $V_{p\text{-}p}$ is the peak-to-peak voltage.

Material	Longitudinal piezoelectric coefficient (d_{33})	Output performance	References
L-α-aspartyl-L-phenylalanine methyl ester/PDMS	37.9 pC/N (Computational)	V_{oc} = 0.62 V	32
Porous organic cages (RCC1-Cl /PDMS)	3.5 pC/N (Experimental)	V_{oc} = 4.1 V	33
Porous organic cages ($RCC3\text{-}SO_4$/PDMS)	3.3 pC/N (Experimental)	V_{oc} = 5.4 V	
4-((4-hydroxyphenyl)diazenyl) benzonitrile/PDMS	66.5 pm/V (Experimental)	V_{oc} = 5.7 V	34
DBCz – TCNQ cocrystal/PET	41 pm/V (Experimental)	V_{oc} = 6 V	35
Boron-Nitrogen (B-N adducts) $[RC_6H_5CH(CH_3)NH_2BF_3]$/ITO coated PET	3.46 pC/N (Experimental)	$V_{p\text{-}p}$ = 6.2 V	36
3-nitroaniline/PCL	6.81 pC/N (Experimental)	V_{oc} = 7 V	37
Diphenyl diisopropylamino phosphonium hexafluorophosphate ($DPDP{\cdot}PF_6$/PDMS)	~5 pC/N (Experimental)	$V_{p\text{-}p}$ = 8.5 V	38
Organic phosphazene salt ([PMe]I/TPU)	~0.13 pC/N (Experimental)	V_{oc} = 13.7 V	39
N,*N′*-bis(4 nitrophenyl)methanediamine (BPNA/PVA)	~7 pC/N (Experimental)	$V_{p\text{-}p}$ = 17.2 V	40
[2,2,3,3,4,4-hexafluoropentane-1,5-diol] (HFPD/PVA)	34.3 pC/N (Experimental)	$V_{p\text{-}p}$ = 18 V	41
β-glycine/gelatin composite films	8.6 pC/N (Experimental)	V_{oc} = 21.9 V	42
Gyroid-shaped (p-TEA)(p-TEAH)$\cdot PF_6$/PCL	4 pC/N (Experimental)	V_{oc} = 22.8 V	43
Organoammonium salt (Gyroid-shaped $[Me_3CCH(Me)NH_3][BF_4]$/PCL)	3.7 pC/N (Experimental)	V_{oc} = 41 V	44

Table S21. Calculations of longitudinal piezoelectric voltage constant g_{33} (V m/N) from average capacitance, average thickness (m), and average piezoelectric response (pC/N) of the circular discs of diameter = 3.3 cm of the multicomponent systems.

Sample	Average Capacitance (F)	Electrode Area (m^2)	Average thickness (m)	d_{33} (C/N)	g_{33} (V·m/N)
L-histidinium trifluoroacetate	5.31216E-13	2.50E-05	0.00240	3.04E-12	60E-3
L-lysinium trifluoroacetate	5.93E-13	2.50E-05	0.00343	5.65E-13	7E-3
L-histidinium trichloroacetate	3.47187E-13	2.50E-05	0.00298	2.07E-13	50E-3
L-lysinium trichloroacetate	2.21E-12	2.50E-05	0.00281	2.02E-12	8E-3

Table S22. Temperature dependant longitudinal piezoelectric studies of L-histidinium trifluoroacetate discs annealed for 24 hrs at different temperature ranging from 50 °C to 100 °C.

Amino acid multicomponent system	Sample	Average Upright d_{33}^{eff} (pC/N)	Average Inverted d_{33}^{eff} (pC/N)
L-histidinium trifluoroacetate discs at 50 °C	1	1.42 ± 0.45	-1.23 ± 0.31
	2	2.03 ± 0.77	-2.39 ± 1.16
	3	1.79 ± 0.94	-1.85 ± 0.69
	4	1.54 ± 0.48	-1.61 ± 0.61
	5	3.59 ± 1.32	-3.22 ± 1.94
L-histidinium trifluoroacetate discs at 75 °C	1	0.78 ± 0.38	-1.14 ± 0.75
	2	1.60 ± 1.72	-0.87 ± 0.34
	3	1.40 ± 1.06	-1.21 ± 0.52
	4	1.69 ± 0.71	-0.98 ± 0.26
	5	1.51 ± 0.98	-2.10 ± 1.03
L-histidinium trifluoroacetate discs at 100 °C	1	1.91 ± 0.97	-1.34 ± 0.67
	2	1.34 ± 0.78	-1.33 ± 0.80
	3	0.85 ± 0.57	-0.70 ± 0.25
	4	1.29 ± 0.63	-1.43 ± 0.45
	5	2.31 ± 1.17	-1.78 ± 1.01

Table S23. Temperature dependant longitudinal piezoelectric studies of L-lysinium trifluoroacetate discs annealed for 24 hrs at different temperature ranging from 50 °C to 100 °C.

Amino acid multicomponent system	Sample	Average Upright d_{33}^{eff} (pC/N)	Average Inverted d_{33}^{eff} (pC/N)
L-lysinium trifluoroacetate discs at 50 °C	1	1.00 ± 0.41	-1.52 ± 1.14
	2	0.98 ± 0.54	-1.12 ± 0.57
L-lysinium trifluoroacetate discs at 75 °C	1	0.95 ± 0.55	-0.68 ± 0.29
	2	0.78 ± 0.32	-0.59 ± 0.17
L-lysinium trifluoroacetate discs at 100 °C	1	0.52 ± 0.14	-0.38 ± 0.10
	2	0.43 ± 0.11	-0.27 ± 0.10

Table S24. Temperature dependant longitudinal piezoelectric studies of L-histidinium trichloroacetate discs annealed for 24 hrs at different temperature ranging from 50 °C to 100 °C.

Amino acid multicomponent system	Sample	Average Upright d_{33}^{eff} (pC/N)	Average Inverted d_{33}^{eff} (pC/N)
L-histidinium trichloroacetate discs at 50 °C	1	1.96 ± 0.40	-2.21 ± 0.72
	2	2.75 ± 0.58	-2.76 ± 0.39
	3	3.78 ± 0.95	-3.53 ± 0.90
	4	3.01 ± 0.66	-3.07 ± 0.62
L-histidinium trichloroacetate discs at 75 °C	1	0.84 ± 0.23	-1.40 ± 0.80
	2	0.96 ± 0.26	-0.83 ± 0.38
	3	1.47 ± 0.65	-0.64 ± 0.40
	4	1.23 ± 0.53	-1.39 ± 0.40
L-histidinium trichloroacetate discs at 100 °C	1	0.69 ± 0.29	-0.79 ± 0.26
	2	0.90 ± 0.35	-0.82 ± 0.34
	3	0.51 ± 0.19	-0.66 ± 0.26
	4	0.86 ± 0.46	-0.86 ± 0.43

Table S25. Temperature dependant longitudinal piezoelectric studies of L-lysinium trichloroacetate discs annealed for 24 hrs at different temperature ranging from 50 °C to 100 °C.

**Amino acid multicomponent system**	**Sample**	**Average Upright d_{33}^{eff} (pC/N)**	**Average Inverted d_{33}^{eff} (pC/N)**
L-lysinium trichloroacetate discs at 50 °C	1	1.35 ± 0.16	-1.47 ± 0.47
	2	1.25 ± 0.12	-1.74 ± 0.27
	3	1.23 ± 0.21	-1.61 ± 0.39
	4	1.38 ± 0.11	-1.63 ± 0.41
L-lysinium trichloroacetate discs at 75 °C	1	0.64 ± 0.37	-0.49 ± 0.28
	2	0.83 ± 0.32	-0.93 ± 0.51
	3	0.71 ± 0.22	-0.90 ± 0.33
	4	1.08 ± 0.53	-0.94 ± 0.38
L-lysinium trichloroacetate discs at 100 °C	1	0.35 ± 0.17	-1.18 ± 0.64
	2	0.70 ± 0.49	-0.72 ± 0.43
	3	1.04 ± 0.53	-1.40 ± 0.69
	4	1.16 ± 0.47	-1.41 ± 1.24

References

1. Püspöki Z, Storath M, Sage D, Unser M. Transforms and operators for directional bioimage analysis: a survey. *Focus on bio-image informatics* 2016**:** 69-93.

2. Sage D. Orientationj: A series of imagej plugins for directional image analysis. *Biomedical Image Group at EPFL, Switzerland* 2020.

3. Rezakhaniha R, Agianniotis A, Schrauwen JTC, Griffa A, Sage D, Bouten CV*, et al.* Experimental investigation of collagen waviness and orientation in the arterial adventitia using confocal laser scanning microscopy. *Biomechanics and modeling in mechanobiology* 2012, **11**(3)**:** 461-473.

4. Vishnoi S, Kumari G, Guest R, Cazade P-A, Guerin S. High-Throughput Computational Screening of Small Molecular Crystals for Sustainable Piezoelectric Materials. *Angewandte Chemie International Edition* 2025, **64**(18)**:** e202501232.

5. Hutter J, Iannuzzi M, Schiffmann F, VandeVondele J. cp2k: atomistic simulations of condensed matter systems. *WIREs Computational Molecular Science* 2014, **4**(1)**:** 15-25.

6. VandeVondele J, Hutter J. An efficient orbital transformation method for electronic structure calculations. *The Journal of Chemical Physics* 2003, **118**(10)**:** 4365-4369.

7. VandeVondele J, Hutter J. Gaussian basis sets for accurate calculations on molecular systems in gas and condensed phases. *J Chem Phys* 2007, **127**(11)**:** 114105.

8. Ernzerhof M, Scuseria GE. Assessment of the Perdew–Burke–Ernzerhof exchange-correlation functional. *The Journal of Chemical Physics* 1999, **110**(11)**:** 5029-5036.

9. Perdew JP, Burke K, Ernzerhof M. Generalized Gradient Approximation Made Simple. *Physical Review Letters* 1996, **77**(18)**:** 3865-3868.

10. Grimme S, Ehrlich S, Goerigk L. Effect of the damping function in dispersion corrected density functional theory. *Journal of Computational Chemistry* 2011, **32**(7)**:** 1456-1465.

11. Grimme S, Antony J, Ehrlich S, Krieg H. A consistent and accurate ab initio parametrization of density functional dispersion correction (DFT-D) for the 94 elements H-Pu. *J Chem Phys* 2010, **132**(15)**:** 154104.

12. Štich I, Car R, Parrinello M, Baroni S. Conjugate gradient minimization of the energy functional: A new method for electronic structure calculation. *Physical Review B* 1989, **39**(8)**:** 4997-5004.

13. Bhattacharya S, Cazade P-A, Hari K, Ryan T, Keeney L, O'Mahony C*, et al.* Harvesting of shear piezoelectricity in a molded multicomponent crystal disc. *Applied Materials Today* 2024, **39:** 102344.

14. Nye JF. *Physical properties of crystals: their representation by tensors and matrices*. Oxford university press, 1985.

15. Vishnoi S, Guerin S. Predicting and rationalizing piezoelectricity in racemic bioorganic molecular crystals. *Angewandte Chemie International Edition* 2026, **65**(26)**:** e24346.

16. Spackman PR, Turner MJ, McKinnon JJ, Wolff SK, Grimwood DJ, Jayatilaka D*, et al.* CrystalExplorer: a program for Hirshfeld surface analysis, visualization and quantitative analysis of molecular crystals. *Journal of Applied Crystallography* 2021, **54**(3)**:** 1006-1011.

17. de Brito AJ. Handbook of Operational Amplifier Applications. *Texas Instruments*.

18. Yang Y, Chen Z, Kuai Q, Liang J, Liu J, Zeng X. Circuit techniques for high efficiency piezoelectric energy harvesting. *Micromachines* 2022, **13**(7)**:** 1044.

19. Sun Y, Hieu NH, Jeong C-J, Lee S-G. An integrated high-performance active rectifier for piezoelectric vibration energy harvesting systems. *IEEE Transactions on Power Electronics* 2011, **27**(2)**:** 623-627.

20. Liang J, Liao W-H. Impedance matching for improving piezoelectric energy harvesting systems. Active and Passive Smart Structures and Integrated Systems 2010; 2010: SPIE; 2010. p. 181-192.

21. Feng Y, Zhao Y, Yan H, Cai H. A driving power supply for piezoelectric transducers based on an improved LC matching network. *Sensors* 2023, **23**(12)**:** 5745.

22. Hari K, Ryan T, Bhattacharya S, Guerin S. Molded, Solid-State Biomolecular Assemblies with Programmable Electromechanical Properties. *Physical Review Letters* 2024, **133**(13)**:** 137001.

23. Guerin S, Khorasani S, Gleeson M, O'Donnell J, Sanii R, Zwane R*, et al.* A piezoelectric ionic cocrystal of glycine and sulfamic acid. *Crystal Growth & Design* 2021, **21**(10)**:** 5818-5827.

24. Niu L, Chen X, Allen S, Tendler SJ. Using the bending beam model to estimate the elasticity of diphenylalanine nanotubes. *Langmuir* 2007, **23**(14)**:** 7443-7446.

25. Yan X, Zhu P, Li J. Self-assembly and application of diphenylalanine-based nanostructures. *Chemical Society Reviews* 2010, **39**(6)**:** 1877-1890.

26. Kol N, Adler-Abramovich L, Barlam D, Shneck RZ, Gazit E, Rousso I. Self-assembled peptide nanotubes are uniquely rigid bioinspired supramolecular structures. *Nano letters* 2005, **5**(7)**:** 1343-1346.

27. Ji W, Xue B, Yin Y, Guerin S, Wang Y, Zhang L*, et al.* Modulating the electromechanical response of bio-inspired amino acid-based architectures through supramolecular co-assembly. *Journal of the American Chemical Society* 2022, **144**(40)**:** 18375-18386.

28. Azuri I, Meirzadeh E, Ehre D, Cohen SR, Rappe AM, Lahav M*, et al.* Unusually large Young's moduli of amino acid molecular crystals. *Angewandte Chemie International Edition* 2015, **54**(46)**:** 13566-13570.

29. Bera S, Mondal S, Xue B, Shimon LJ, Cao Y, Gazit E. Rigid helical-like assemblies from a self-aggregating tripeptide. *Nature Materials* 2019, **18**(5)**:** 503-509.

30. Basavalingappa V, Bera S, Xue B, O'Donnell J, Guerin S, Cazade PA*, et al.* Diphenylalanine-Derivative Peptide Assemblies with Increased Aromaticity Exhibit Metal-like Rigidity and High Piezoelectricity. *American Chemical Society Nano* 2020, **14**(6)**:** 7025.

31. Motola Y, Banks-Sills L, Fourman V. A note on tensile testing of poled and unpoled piezoelectric ceramics. *Strain* 2009, **45**(1)**:** 85-92.

32. Yang B, Zhang Y, Liu S, Huo Y, Ji W. Aspartame Peptide-Based Piezoelectric Supramolecular Material Toward Energy Harvesting. *Small* 2026**:** e12690.

33. Ye Y-z, Liu H-y, Gong Y-j, Xu Z-z, Zhao Y, Yu N*, et al.* Design of piezoelectric organic cage salts for energy harvesting. *Chem* 2024, **10**(4)**:** 1118-1131.

34. Ambastha P, Kushwaha V, Magar A, Gupta Y, Parida T, Kanjilal A*, et al.* Flexible organic piezoelectric nanogenerator with high power density and excellent ferroelectric and memristor characteristics. *NPG Asia Materials* 2026, **18**(1)**:** 4.

35. Ma S, Liu Z, Gao C, Lin H, Xiang W, Chen J*, et al.* Polar organic charge-transfer complex of the asymmetrical component for flexible piezoelectric energy harvesting and self-powered wearable sensors. *ACS Applied Materials & Interfaces* 2024, **16**(2)**:** 2583-2592.

36. Mukherjee S, Vaitheeswaran G, Kabra D, Hernandez WI, Garcia-Castro AC, Sharma VB*, et al.* A Chiral BN Adduct as a New Frontier in Ferroelectrics and Piezoelectric Energy Harvesting. 2024.

37. Bernardo CR, Baptista RM, de Matos Gomes E, Lopes PE, Raposo MMM, Costa SP, *et al.* Anisotropic PCL nanofibers embedded with nonlinear nanocrystals as strong generators of polarized second harmonic light and piezoelectric currents. *Nanoscale Advances* 2020, **2**(3)**:** 1206-1213.

38. Vijayakanth T, Srivastava AK, Ram F, Kulkarni P, Shanmuganathan K, Praveenkumar B, *et al.* A flexible composite mechanical energy harvester from a ferroelectric organoamino phosphonium salt. *Angewandte Chemie* 2018, **130**(29)**:** 9192-9196.

39. OGALE S, Guerin S, DESWAL S, Anthopoulos TD, Naphade DR, Cazade P-A, *et al.* Design and Piezoelectric Energy Harvesting Properties of a Ferroelectric Cyclophosphazene Salt. 2023.

40. Bhunia S, Karan SK, Chowdhury R, Ghosh I, Saha S, Das K, *et al.* Mechanically flexible piezoelectric organic single crystals for electrical energy harvesting. *Chem* 2024, **10**(6)**:** 1741-1754.

41. Zhang H-Y, Tang Y-Y, Gu Z-X, Wang P, Chen X-G, Lv H-P, *et al.* Biodegradable ferroelectric molecular crystal with large piezoelectric response. *Science* 2024, **383**(6690)**:** 1492-1498.

42. Sun Y, Tian J, Shi C, Tang C. Bio-piezoelectric β-glycine/gelatin composite films fabricated via synergistic molecular self-assembly and thermally assisted evaporation-induced crystallization. *Acta Biomaterialia* 2026.

43. Sahoo S, Panday R, Kothavade P, Sharma VB, Sowmiyanarayanan A, Praveenkumar B, *et al.* A Highly Electrostrictive Salt Cocrystal and the Piezoelectric Nanogenerator Application of Its 3D-Printed Polymer Composite. *ACS Applied Materials & Interfaces* 2024, **16**(20)**:** 26406-26416.

44. Sahoo S, Kothavade PA, Naphade DR, Torris A, Praveenkumar B, Zaręba JK, *et al.* 3D-printed polymer composite devices based on a ferroelectric chiral ammonium salt for high-performance piezoelectric energy harvesting. *Materials Horizons* 2023, **10**(8)**:** 3153-3161.
